# Unequal Contribution of Widespread and Narrow-Ranged Species to Botanical Diversity Patterns

**DOI:** 10.1371/journal.pone.0169200

**Published:** 2016-12-29

**Authors:** André S. J. van Proosdij, Niels Raes, Jan J. Wieringa, Marc S. M. Sosef

**Affiliations:** 1 Biosystematics Group, Wageningen University, Wageningen, the Netherlands; 2 Naturalis Biodiversity Center, Leiden, the Netherlands; 3 Botanic Garden Meise, Meise, Belgium; Chinese Academy of Forestry, CHINA

## Abstract

In conservation studies, solely widespread species are often used as indicators of diversity patterns, but narrow-ranged species can show different patterns. Here, we assess how well subsets of narrow-ranged, widespread or randomly selected plant species represent patterns of species richness and weighted endemism in Gabon, tropical Africa. Specifically, we assess the effect of using different definitions of widespread and narrow-ranged and of the information content of the subsets. Finally, we test if narrow-ranged species are overrepresented in species-rich areas. Based on distribution models of Gabonese plant species, we defined sequential subsets from narrow-ranged-to-widespread, widespread-to-narrow-ranged, and 100 randomly arranged species sequences using the range sizes of species in tropical Africa and within Gabon. Along these sequences, correlations between subsets and the total species richness and total weighted endemism patterns were computed. Random species subsets best represent the total species richness pattern, whereas subsets of narrow-ranged species best represent the total weighted endemism pattern. For species ordered according to their range sizes in tropical Africa, subsets of narrow-ranged species represented the total species richness pattern better than widespread species subsets did. However, the opposite was true when range sizes were truncated by the Gabonese national country borders. Correcting for the information content of the subset results in a skew of the sequential correlations, its direction depending on the range-size frequency distribution. Finally, we find a strong, positive, non-linear relation between weighted endemism and total species richness. Observed differences in the contribution of narrow-ranged, widespread and randomly selected species to species richness and weighted endemism patterns can be explained by the range-size frequency distribution and the use of different definitions of widespread or narrow-ranged. We call for a reconsideration of the use of widespread species as an indicator of diversity patterns, and advocate using the full ranges of species when assessing diversity patterns.

## Introduction

The current biodiversity crisis and limited availability of resources forces governments and NGOs to define conservation priorities [1]. Commonly, highly biodiverse regions (harbouring many species), centres of endemism (harbouring many narrow-ranged species), and crisis ecoregions (regions under threat of habitat conversion and climate change) are identified as priority areas for conservation [2–4]. Unfortunately, for many parts of the world, especially the tropics, little is known about the spatial distribution of most individual species or of the spatial distribution of diversity; a phenomenon known as the Wallacean shortfall [5]. Most species are narrow-ranged, resulting in a right-skewed range-size frequency distribution [6,7]. Several studies have shown that species richness patterns based on narrow-ranged species differ from those based on widespread species and that most patterning in species richness is caused by a comparatively small subset of widespread species [8–11]. Generally, the distribution of narrow-ranged species appears less correlated with climatic variables, but more strongly correlated with topographic and historical factors [8,9]. Therefore, using a subset of relatively common, widespread species as an indicator of species richness may well yield inappropriate conservation priorities for rare, narrow-ranged species.

Consideration of endemism has also been suggested as a replacement for assessment of total species richness in the context of identifying conservation priorities [2,12]. Levels of endemism have been calculated in various ways including measures that weigh each species according to its rarity [13,14]. Several studies have shown a positive, non-linear relationship between the number of narrow-ranged species and the total number of species in an area, resulting in species-rich areas having a higher proportion of narrow-ranged species than average [15,16]. However, studies on vertebrates have shown that centres of endemism are not necessarily congruent with centres of species richness [17–21].

The contribution of each species to the pattern of species richness depends on the individual prevalence of species [10,22,23], with prevalence defined as the fraction of the study area where the species occurs [24]. A species present in 50% of the study area has the highest contribution to the richness pattern, whereas species present in 10% or 90% have an equally lower contribution of information to the pattern. This effect is known as the information content of a set of species and is defined as Σ(p*(1-*p*)) with *p* being the fraction of presence cells of each species [10]. The difference between species richness patterns based on subsets of widespread and narrow-ranged species is only partly explained by differences in information content of these subsets [9–11,25]. Often, when assessing richness patterns, the range sizes or prevalences are calculated for areas defined by political boundaries, thus not encompassing the full ranges of species. This logically leads to patterns only applicable at a local scale, though these may be important for political reasons. However, those interested in global diversity patterns need to take into account the full ranges of species [9], which is what we aim for in our present study of Gabonese plant species.

For most species, preserved collections are not adequate reflections of species distribution patterns. By contrast, species Distribution Models (SDM) offer a solution as these predict the spatial distribution of species by linking a limited number of observations to environmental data with high spatial resolution [26]. Typically, the constantly growing body of digitized presence-only specimen data from natural history collections are used as observations [27]. Diversity patterns can be inferred by stacking SDMs that are converted into binary presence/absence maps [16,28]. This method offers unique opportunities to assess congruence between diversity patterns based on different subsets of species.

Here, using SDMs of plant species from Gabon, central Africa, we infer patterns of species richness and weighted endemism for Gabon. More specifically, we address the following questions: 1) Do diversity patterns based on subsets of narrow-ranged or widespread plant species differ from those based on random subsets? 2) Are these differences still apparent when corrected for the information content of each subset? 3) Are these differences sensitive to the extent of the study area in which the range sizes are defined, here Gabon versus tropical Africa as a whole? 4) Are narrow-ranged species overrepresented in species-rich areas?

## Materials and Methods

### Study area

We selected Gabon to serve as a case study. Gabon is a highly biodiverse country in the Lower Guinean phytogeographical region [29,30] with around 80% of its 267,667 km^2^ covered by lowland rain forest and the remaining 20% mainly by savannahs and urban areas (S1 Fig). It hosts an estimated number of 7000–7500 vascular plants species [31], of which 5323 have been recorded so far. Of these, 13% are endemic or near endemic to Gabon and many more are native only in the Lower Guinean biogeographic region [31], showing the importance of the contribution of narrow-ranged species to diversity patterns. In contrast to most other species-rich, tropical African countries, the botanical diversity of Gabon is well-documented with > 95% of the known herbarium collections digitally available through the Naturalis Biodiversity Center database. This renders Gabon an excellent study area to address the research questions formulated above. We defined our African study area from 15°N to 19°S and from 17.5°W to 43°E, encompassing the known range of the majority of Gabonese plant species and covering 180,399 raster cells at 5 arc-minute spatial resolution (excluding oceans and large water bodies).

### Species distribution data

To avoid the exclusion of species known to occur in neighbouring countries and possibly also to be found in Gabon, but not yet collected there, we selected all plant species recorded at least once from Gabon including a buffer area of five degrees (approx. 600 km). Species known to only occur in cultivation in Gabon were excluded. Subspecific taxa were combined in the germane species. From the species list so compiled, we used all available herbarium specimen data from Gabon and other tropical African countries to avoid modelling truncated niches of species [32] and to make use of all available data for model training [33]. Records comprising doubtful identifications as well as duplicate records from the same raster cell were excluded. Only records with latitude/longitude data accurate to at least five arc-minute spatial resolution were used.

### Environmental data and two model training areas

We used WorldClim temperature data [34], CHIRPS precipitation data [35,36], and quantitative soil data from the Harmonized World Soil Database [37]. Environmental data layers were cropped to the extent of the study area (hereafter ‘African training area’) and, where necessary, aggregated to five arc-minute spatial resolution. As a measure of topographic heterogeneity we used the standard deviation of altitude based on the 90 m SRTM altitude data (<srtm.csi.cgiar.org>) within each five arc-minute raster cell. Out of the 39 original variables we selected those correlated with Spearman’s |*rho*| < 0.7 [38], to avoid overfitting of models due to multi-collinearity, resulting in 15 selected variables (S1 Table and S1 Text). We adjusted the extent of the training area of species with a prevalence < 0.1 or > 0.9 to avoid statistical artefacts in modelling these species [24]. The prevalence of species was estimated by using the fraction of raster cells where the species was predicted as present in tropical Africa based on a thresholded SDM [39]. For species with a predicted prevalence < 0.1 in the African training area, we used the smaller training area of Gabon including a buffer area of five degrees (hereafter ‘Gabonese training area’) resulting in 18,144 5-arc minute raster cells and using the same selected environmental variables. No species had a prevalence > 0.9.

### Model building

SDMs were generated using MaxEnt [40], which has shown to outperform other methods when using presence-only data like ours, even when applied to small data sets [41]. We modified the MaxEnt default settings by allowing only linear and quadratic features for all sample sizes, and excluding hinge, product and threshold features to prevent over-parameterization of the models [42]. To compensate for a potential collecting bias in our specimen data, possibly resulting in an ecological bias [43,44], we applied the same bias to the background data used to train the models by means of target background sampling [45]. Consequently, pseudo-absences were selected from raster cells with at least one herbarium record. The logistic MaxEnt output for each species was converted into a binary presence/absence map by applying the ‘ten percentile training presence’ threshold. This threshold forces 10% of the training records to fall outside the predicted suitable area, which is thought to allow for 10% of the records to contain identification, georeferencing or other errors without serious consequences for the model [42,46]. A Multivariate Environmental Similarity Surface analysis [47] showed considerable areas with negative MESS values for models trained on the Gabonese training area (S2 Fig), which is why SDMs trained on the Gabonese training area were projected on the larger tropical African area without extrapolation to environmental conditions not present in the smaller training area.

### Model evaluation

Models were evaluated using two criteria. First, each model was tested against a bias-corrected null model following Raes & ter Steege [48] and accepted if its AUC value ranked > 95 when grouped with the 99 null model AUC values. This implies that the model performed significantly better than random expectation (*p* < 0.05). Second, from the significant SDMs, a model was accepted when the number of unique training records equalled or exceeded the minimum number of records required to generate models significantly better than random expectation. This minimum number of records increases with increasing prevalence of the species [33]. Following the procedure of van Proosdij *et al*. [33], we identified the following required minimum numbers of records for species of different prevalence classes for the models trained on the African training area and between brackets the minimum numbers for the Gabonese training area: 7 (5) for prevalence < 0.1, 7 (8) for prevalence 0.1–0.2, 9 (10) for prevalence 0.2–0.3, 12 (11) for prevalence 0.3–0.4, 12 (14) for prevalence 0.4–0.5, and 15 (17) for prevalence > 0.5.

### Patterns of species richness and weighted endemism

Three types of diversity patterns were computed by stacking the selected thresholded SDMs. Firstly, total species richness was computed by summing the number of species predicted to be present in each raster cell. Secondly, weighted endemism was computed following Crisp *et al*. [13] and Wieringa & Poorter [14] by summing up the rarity values of the species present in a unit or raster cell, with rarity value defined as the inverse of the number of presence cells. Finally, residuals of weighted endemism were defined as the weighted endemism relative to the species richness of the raster cell [16], also termed corrected weighted endemism [49] (hereafter called ‘residual weighted endemism’). We computed the residual weighted endemism values by first fitting a curve to the values of weighted endemism plotted against total species richness. Akaike Information Criterion was used to select the best polynomial curve. Then, relative residuals were computed by taking for each cell the difference between the actual weighted endemism value and the fitted value, relative to the fitted value. The resulting three diversity patterns were cropped to the national borders of Gabon.

### Species sequences and correlation with species richness and weighted endemism

Species with accepted SDMs were ranked according to their predicted prevalence in tropical Africa. We generated one narrow-ranged to widespread sequence, one widespread to narrow-ranged sequence, and 100 random sequences [10,22,50]. This procedure was repeated by ranking the species according to their prevalence within Gabon. For subsets of *n* species, with increasing values of *n* along the sequences, species richness maps (‘n_richness’) and weighted endemism maps (‘n_weighted_endemism’) were generated. Along the sequences we computed the Pearson correlation of n_richness with the total species richness pattern and of n_weighted_endemism with the total weighted endemism pattern, all cropped to the national borders of Gabon. Resulting Pearson’s *r* values of the subsets along the sequences were plotted against the number of species as well as against the information content of the subsets. The information content of a subset was computed by summing the information contents of the species in the subset. All analysis were performed in *R*, using functions provided in the *R* script available as Supporting Information (S2 Text).

## Results

**Fig 1:**
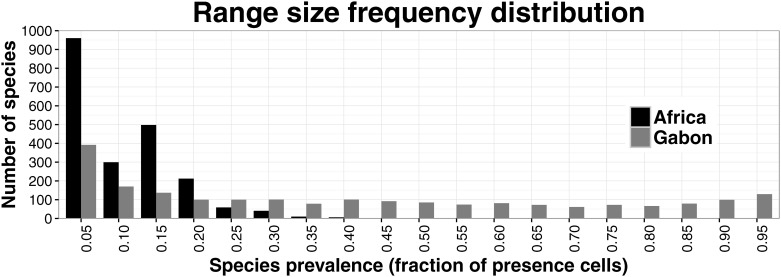
Range size frequency distribution of Gabonese plant species. The range size frequency distribution is shown for the Gabonese plant species with accepted SDMs. Range size or prevalence is defined as the fraction of raster cells where the species is predicted present in tropical Africa (black) and Gabon (grey) respectively.

In total, our dataset contains 5323 species from Gabon and an additional 3361 from the five degrees buffer zone. A total of 317,582 herbarium specimen records related to these 8684 species were aggregated in our dataset and used for model-building. 3572 species did not have sufficient records to model a reliable SDM; for another 2628, their SDMs did not pass the null model test, while 395 of the remaining SDMs predicted the species to be absent from Gabon (although present in the buffer zone). In total, SDMs of 2089 species were used for further analyses including one liverwort species, 22 moss species, eight clubmoss species, 63 fern species, one gymnosperm species and 1994 angiosperm species (S2 Table, SDMs available from the Dryad Digital Repository: http://dx.doi.org/10.5061/dryad.v4f53). When trained on tropical Africa, SDMs of 1306 species resulted in a predicted prevalence < 0.1, and hence their SDMs were rerun using the smaller Gabonese training area. Of these new SDMs, 624 also had a predicted prevalence of < 0.1 in the Gabonese training area, which we regard as acceptable given the scope of this study. The range size frequency distribution based on the predicted prevalences for both tropical Africa and Gabon is strongly right-skewed towards narrow-ranged species (Fig 1). It is to be noted that for range sizes based on tropical Africa, the apparent peak at a prevalence of 0.10–0.15 is actually caused by the exclusion of many species with a prevalence < 0.10. These excluded species are recorded from the five degrees buffer zone but are predicted to be absent from Gabon or have too few records inside the Gabonese training area to generate a significant SDM (S3 Fig).

**Fig 2:**
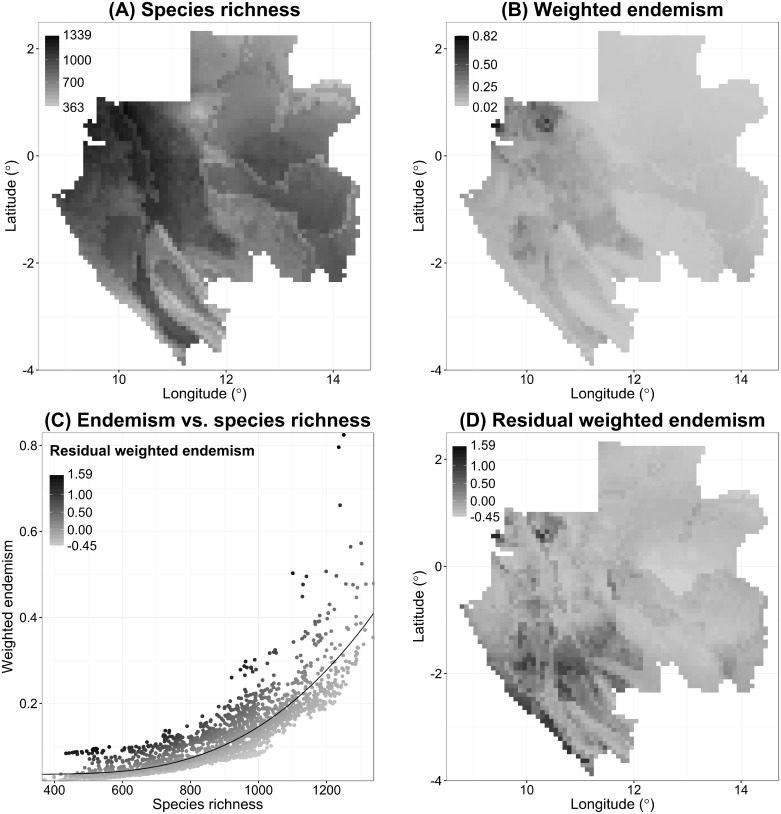
Botanical diversity patterns for Gabon based on 2089 species. The following diversity patterns are shown based on thresholded SDMs of 2089 Gabonese plant species: (A) total species richness; (B) weighted endemism; (C) weighted endemism (y-axis) plotted against total species richness (x-axis) with shades of grey indicating values of residual weighted endemism and the black curve representing a fourth-order polynomial function; (D) residual weighted endemism.

The highest species richness in Gabon is predicted for north-western Gabon (foothills of the Crystal Mountains and the vicinity of Libreville), as well as hills in central and western Gabon (Doudou Mountains and western parts of Chaillu Massif) (Fig 2A). Areas with high values of weighted endemism are largely congruent with centres of species richness with maximum values in the Crystal Mountains and the vicinity of Libreville (Fig 2B). Species richness and weighted endemism show a strong positive, non-linear relation, best represented by a fourth-order polynomial function (Fig 2C, *Y* = 2.583e-04*X– 6.645e-07*X^2^ + 6.379e-10*X^3^–8.581e-14*X^4^, adjusted R^2^ = 0.92, *p* < 0.001). Fig 2D shows high positive values of residual weighted endemism in the two aforementioned centres of endemism and in the coastal region south of one degree south latitude, meaning that in those areas more narrow-ranged species are present than would be expected from the species richness.

**Fig 3:**
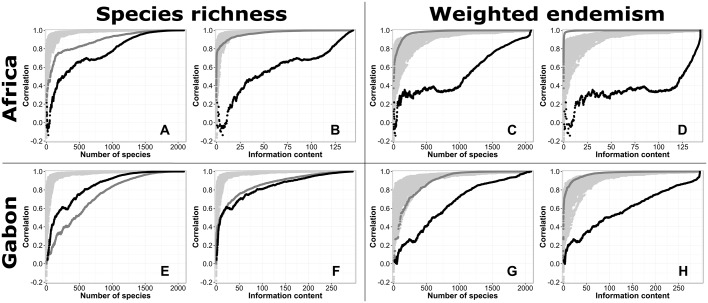
Correlations between subset and total diversity patterns. Correlations are presented between species richness patterns based on subsets of n Gabonese plant species (n_richness) and total Gabonese species richness (A,B,E,F), as well as between weighted endemism patterns based on subsets (n_weighted_endemism) and total weighted endemism (C,D,G,H). Subsets were composed along the narrow-ranged to widespread sequence (dark grey lines), widespread to narrow-ranged sequence (black lines), and 100 random sequences (light grey lines). Defining the species sequences was done on the prevalences of species in either tropical Africa (A-D), or Gabon (E-H). Correlations are plotted against the number of species (A,C,E,G) or the information content of the subset (B,D,F,H).

Along each of the sequences based on the prevalence of species in tropical Africa, correlation values of n_richness patterns to total richness pattern increase, but they do so more rapidly for the narrow-ranged-to-widespread sequence (Fig 3A, Kolmogorov-Smirnov test: D = 0.37, *p* < 0.001). A correlation of *r* = 0.7 is achieved with the 5% most narrow-ranged species, versus the 35% most widespread species. However, these subsets are both outperformed by random subsets. When corrected for the information content of the subsets, the narrow-ranged-to-widespread sequence performs as well as the random sequences, while the performance of the widespread-to-narrow-ranged sequence decreases (Fig 3B).

By contrast, along each of the sequences based on the prevalence within Gabon, correlation values increase more rapidly for the widespread-to-narrow-ranged sequence (Fig 3E, Kolmogorov-Smirnov test: D = 0.18, *p* < 0.001). Here, a correlation of 0.7 is achieved with the 20% most widespread species versus the 35% most narrow-ranged ones. Here too, both are outperformed by random subsets. Correcting for the information content of the subsets results in narrow-ranged species slightly outperforming widespread ones (Fig 3F).

Using sequences based on the prevalence in tropical Africa, we found, as was to be expected, patterns of n_weighted_endemism based on narrow-ranged species to be more strongly correlated with the total weighted endemism pattern than were patterns based on widespread species (Fig 3C, Kolmogorov-Smirnov test: D = 0.90, *p* < 0.001), even outperforming random subsets. When corrected for the information content, narrow-ranged species remain more strongly correlated with weighted endemism, whereas the correlation of widespread species to weighted endemism decreases (Fig 3D). For the sequences based on prevalence within Gabon, patterns of n_weighted_endemism based on narrow-ranged species are more strongly correlated with the total weighted endemism pattern than are patterns based on widespread species (Fig 3G, Kolmogorov-Smirnov test: D = 0.54, *p* < 0.001), but are outperformed by random subsets when these contain less than 25% of the species. Correcting for the information content of the subsets results in subsets of narrow-ranged species showing the strongest correlation with the total weighted endemism pattern (Fig 3H).

## Discussion

### Diversity patterns in Gabon

The inferred pattern of plant species richness with centres of diversity in the vicinity of Libreville as well as the mountains of central and western Gabon confirms previous findings based on legumes [51] or endemic species [52]. These centres of species richness and of weighted endemism coincide with the hypothesised Last Glacial Maxima forest refugia in the Crystal Mountains, western parts of the Chaillu Massif and the Doudou Mountains [53,54]. The high levels of residual weighted endemism in the coastal region south of one degree south latitude illustrates the uniqueness of this relatively species-poor area that is floristically not related to other parts of central Africa and contains a comparatively high number of endemic species [55,56].

### Widespread versus narrow-ranged

Our results confirm that richness patterns based on narrow-ranged species differ from those based on widespread species [15,17,23]. However, the correlation of each of these patterns with the total species richness pattern depends on the extent of the study area used to define the prevalence of species. When prevalence was defined for tropical Africa, we found patterns of narrow-ranged species in Gabon to be more strongly correlated with the pattern of total species richness. This contradicts the results of previous studies which found patterns of widespread species being more strongly correlated with total species richness patterns [8–10,22,25,57]. In addition to the unique suite of species and habitats in each study area, four other matters need further consideration so as to put our results into perspective.

Firstly, the range size frequency distribution of the species influences the sequential correlations and depends on the study area and species group. Our data set is strongly right-skewed and thus similar to the dataset of Uruguayan plants used by Perez-Quesada & Brazeiro [57], whose results are in line with ours. By contrast, Kreft *et al*. [9] found patterns of widespread species that were more strongly correlated with the total species richness pattern using a Neotropical palm data set with an approximately normally distributed range size frequency. The work of Lennon *et al*. [10] then, on birds from Scotland, the united Kingdom as a whole, and South Africa, presents results similar to those of Kreft *et al*. for Scottish and British birds, but contrasting results for South African birds. The sequential correlation of their South African bird data set plotted against the information content of the subsets is higher for narrow-ranged species than for widespread species. From their three data sets, the South African birds data set is the most strongly right-skewed [10]. Based on these and our results from different study areas and different species groups, we conclude that strongly right-skewed range size frequency distributions result in stronger correlations between narrow-ranged species subsets and the total species richness pattern.

The second matter is the range size or prevalence criterion that is applied to define the species sequences, a matter to which little attention has been paid up to now. Most studies order species based on their prevalence in the study area alone, which can be much smaller than the full range size of the species [10,22,23,25,50,57], with few positive exceptions [9]. For example, widespread African species are sometimes rare in Gabon and Gabonese endemics sometimes have a large prevalence within the country. We assessed both of these by ordering species based on their prevalence in both tropical Africa and in Gabon and found contradicting results. We conclude that for a correct comparison of aspects of narrow-ranged and widespread species, species should be ordered according to their entire range size.

The third matter to consider is that 6595 species (76%) were excluded from our analysis as their models did not meet the criteria of model accuracy, or the species were recorded only from the five degrees buffer zone but not predicted to be present in Gabon. Little can be said with confidence on the overall distribution of these excluded species, but since 3572 were excluded because of insufficient records, we expect the majority to be narrow-ranged. In general, we expect that if these apparently rare species could be included in the analysis, this would result in an even larger difference between diversity patterns based on narrow-ranged species versus those based on widespread species.

Thirdly, our results are based on the use of SDMs, which usually do not take into account biotic interactions, historical constraints, and dispersal limitations [58]. Therefore, the actual prevalence of species limited in their distribution by such factors, may well be (much) smaller than predicted here, resulting in an even more skewed range size frequency distribution. Ignoring dispersal limitations might also affect the calculated species composition of ecologically isolated areas.

### Random subsets

We found species richness patterns based on random subsets of species to be more strongly correlated with the total species richness pattern than were patterns based on either narrow-ranged or widespread species alone. Some studies report a stronger correlation with the total species richness pattern for widespread species subsets over random subsets [9,22], but others show contradictory results [23]. With respect to the correlation of subsets with weighted endemism, we found, as expected, random subsets being outperformed by those of narrow-ranged species when species are ordered according to their full range size. However, when ordered on prevalence within Gabon, again, random species subsets better represent the total weighted endemism pattern. Comparing the sequential correlation curves of our study with those reported by others cited above, we see strong similarities between the curves of random species subsets and large differences between the sequential correlation curves of widespread and of narrow-ranged species subsets. These differences can be explained by the matters addressed above: the range size frequency distribution of the assessed species and the applied criterion to define species sequences from narrow-ranged to widespread and vice versa.

### Information content

Correcting for the information content of the subsets influences the sequential correlation curves. The magnitude of this correction depends on the information content of the species included in the subset with species present in 50% of the study area (prevalence = 0.5) contributing the largest amount of information [10]. Here, the prevalence in Africa of all but a few species is < 0.5 and hence the group of species with the largest information content consists of those species with the largest prevalence (prev. 0.3–0.5). Correcting for the information content in our study resulted in a skew to the right for the narrow-ranged-to-widespread sequence and a skew to the left for the widespread-to-narrow-ranged sequence (Fig 3B and 3D). The skew is less strong for the curves based on Gabonese prevalences, which contain many species with a prevalence value > 0.5. The sequential correlation curves of random subsets did not change when corrected for information content. The differences in skew found by us and by others [9,10] can be explained by the specific range size frequency distributions.

### Overrepresentation of narrow-ranged species in species-rich areas

In Gabon, narrow-ranged plant species are overrepresented in species-rich areas resulting in a strong, positive, non-linear relation. Therefore, estimating total plant species richness in Gabon based on the number of widespread plant species in an area will result in an underestimate of species richness in Gabon’s centres of diversity. Our results thus confirm similar findings for African birds [15], North American vertebrates and invertebrates [59], and vascular plants from the United Kingdom [23] and Borneo [16]. By contrast, other studies have shown that centres of species richness and centres of endemism are not congruent [18,20,21] or only partially so [17]. These seemingly contradictory results underline the difficulties of identifying universal estimators for patterns of species richness and weighted endemism, but can be explained by some factors that are often ignored, including differences in the suite of species and habitat types present in the study areas, differences in applied spatial resolution and differences in the extent of the study areas [9,60,61]. In addition, concordance of the species richness pattern and endemism pattern is low when only the few most species-rich and most rare—species-rich-cells are compared, but is high when correlation is computed over all cells [59]. Others have found a small overlap between the most species-rich cells with those containing the most rare species [18,20], as well as a weak, or no, correlation between patterns of total species richness and endemism when this is computed over all cells [18,21]. Furthermore, congruence is higher when endemism is defined as weighted endemism including all species [59], as we report here.

### Implications for conservation

Setting priorities in conservation is topical, especially for the Tropics, that harbour by far the most species, but face the highest extinction risks [62]. If one aims to identify the most species-rich areas using small subsets of species, random subsets of species best represent these areas given that the range size or prevalence of the targeted species is defined over their entire range. However, if one aims to identify areas containing the highest endemicity values, and applying the same range size criterion, subsets of narrow-ranged species are to be preferred. Both criteria may ignore areas with high values of residual weighted endemism, thus harbouring only few species but a disproportionally high number of species not present elsewhere, as we have demonstrated here for the coastal zone of Gabon and has also been shown for other areas, including e.g. Borneo [16]. These areas deserve priority for conservation too, as they contain disproportionally many species not present elsewhere.

## Conclusions

For Gabon we have shown that patterns of plant species richness based on subsets of narrow-ranged species differ substantially from those based on subsets of widespread species. If species are ordered according to their full range size, subsets of narrow-ranged species represent the total species richness pattern better, but both are outperformed by random subsets. However, if ordered on range sizes truncated by the country borders of Gabon, subsets of narrow-ranged species are outperformed by subsets of widespread species. This difference in the ordering of species from narrow-ranged to widespread, in concert with the unique range size frequency distribution, suite of species and habitats present in a study area, influences the correlation of subsets of species with patterns of total species richness and weighted endemism. Correcting for the unequal information content of subsets of narrow-ranged and widespread species influences the sequential correlation with diversity patterns, and the exact effect of this correction depends on the range size frequency distribution of the species.

In Gabon, narrow-ranged plant species are overrepresented in species-rich areas. Omitting narrow-ranged species from diversity assessments will result in an underestimate of species richness in species-rich areas. In addition, some centres of residual weighted endemism contain few species in total but a disproportionally high number of narrow-ranged species and hence can be overlooked too when narrow-ranged species are omitted from diversity assessments. We call for a reconsideration of the use of richness patterns based on a selection of widespread species as a measure of total species richness, as this is not universally applicable to all taxonomic groups or study areas. Secondly, we argue for an analysis of the range size frequency distribution of the species and always to use the full ranges of species when assessing diversity patterns and correlations with possible explanatory environmental variables.

## Supporting Information

S1 Fig Map of Gabon. Altitude is shown in meters (Worldclim data, Hijmans *et al*., 2005), Gabonese country borders in black and Crystal Mountains (CRM), Chaillu Massif (CHM) and Doudou Mountains (DOM) are indicated by red polygons. Libreville, the capital of Gabon is indicated on the map.
(DOCX)

S2 Fig Multivariate Environmental Similarity Surface (MESS) analysis. A MESS analysis for models trained on the smaller Gabonese training area projected to the larger tropical African area shows considerable areas with negative MESS values meaning that one or more environmental variables have values outside the range present in the training data (Elith et al., 2010).
(DOCX)

S3 Fig Comparison of range size frequency distributions. The range size frequency distribution of the species with accepted SDMs is shown, with range size or prevalence of the species defined as the fraction of raster cells where the species is predicted to be present in tropical Africa. In black the original RSFD values based on Species Distribution Models trained on either tropical Africa or Gabon and including only species with accepted SDMs that are predicted to be present in Gabon (same as in main text Fig 1). In grey the RSFD values based on SDMs which are all trained on tropical Africa and including all species with accepted SDMs, thus including those species recorded from the five degree buffer zone but predicted to be absent for Gabon itself.
(DOCX)

S1 Table Selected environmental parameters. Variables are selected based on a Spearman’s |*rho*| < 0.7. Correlated variables are given for each selected variable.
(DOCX)

S2 Table List of species predicted present in Gabon. For each of the 2089 species that are predicted present in Gabon based on significant Species Distribution Models, the scientific name, family name and higher taxonomic rank are given.
(DOCX)

S1 Text *R* script for data preparation. (R)

S2 Text *R* script for the analysis and plotting of the results. (R)
