# Supplementary material for: Unequal Contribution of Widespread and Narrow-Ranged Species to Botanical Diversity Patterns
Source: PLoS One. 2016 Dec 29;11(12):e0169200. doi: 10.1371/journal.pone.0169200 (PMC5199077; doi:10.1371/journal.pone.0169200)
Supplement: S1 Fig — Altitude is shown in meters (Worldclim data, Hijmans et al., 2005), Gabonese country borders in black and Crystal Mountains (CRM), Chaillu Massif (CHM) and Doudou Mountains (DOM) are indicated by red polygons. Libreville, the capital of Gabon is indicated on the map. (DOCX) [file pone.0169200.s001.docx]

***van Proosdij, A.S.J., Raes, N., Wieringa, J.J. and Sosef, M.S.M. 2016.***

***Title: Unequal contribution of widespread and narrow-ranged species to botanical diversity patterns.***

***Journal: Plos One.***

***Corresponding author: André S.J. van Proosdij,*** [***andrevanproosdij@hotmail.com***](mailto:andrevanproosdij@hotmail.com)

***S1 Fig. Map of Gabon.*** *Altitude is shown in meters (Worldclim data, Hijmans et al., 2005), Gabonese country borders in black and Crystal Mountains (CRM), Chaillu Massif (CHM) and Doudou Mountains (DOM) are indicated by red polygons. Libreville, the capital of Gabon is indicated on the map.*

*
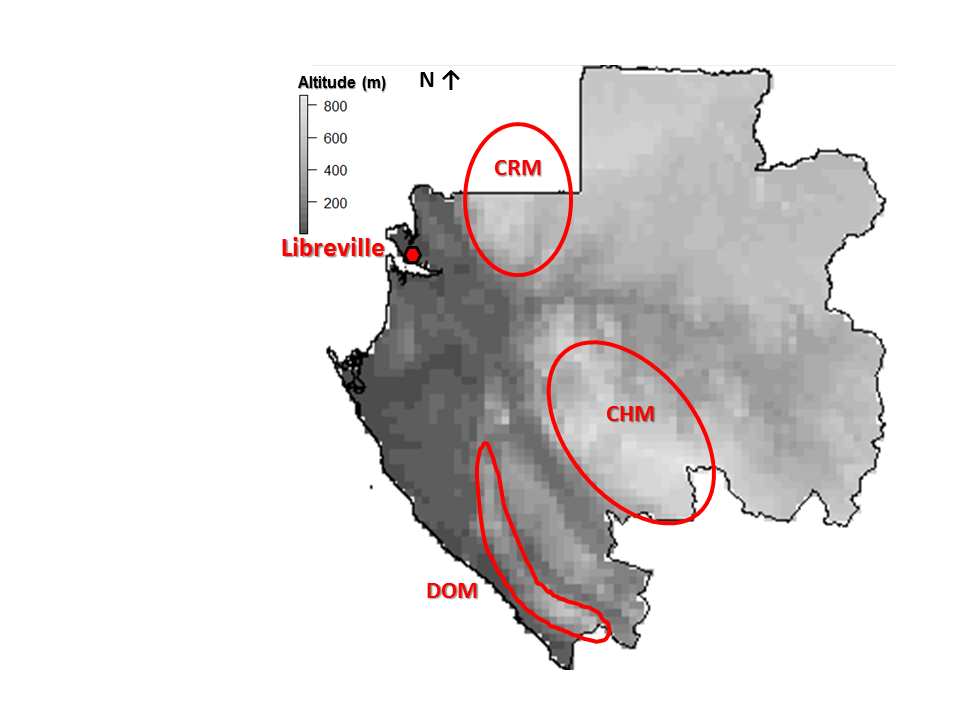
*

References

- *Hijmans, R.J., S.E. Cameron, J.L. Parra, P.G. Jones and A. Jarvis, 2005. Very high resolution interpolated climate surfaces for global land areas. International Journal of Climatology 25: 1965-1978.*
