# Supplementary material for: Unequal Contribution of Widespread and Narrow-Ranged Species to Botanical Diversity Patterns
Source: PLoS One. 2016 Dec 29;11(12):e0169200. doi: 10.1371/journal.pone.0169200 (PMC5199077; doi:10.1371/journal.pone.0169200)
Supplement: S2 Fig — A MESS analysis for models trained on the smaller Gabonese training area projected to the larger tropical African area shows considerable areas with negative MESS values meaning that one or more environmental variables have values outside the range present in the training data (Elith et al., 2010). (DOCX) [file pone.0169200.s002.docx]

***van Proosdij, A.S.J., Raes, N., Wieringa, J.J. and Sosef, M.S.M. 2016.***

***Title: Unequal contribution of widespread and narrow-ranged species to botanical diversity patterns.***

***Journal: Plos One.***

***Corresponding author: André S.J. van Proosdij,*** [***andrevanproosdij@hotmail.com***](mailto:andrevanproosdij@hotmail.com)

***S2 Fig. Multivariate Environmental Similarity Surface (MESS) analysis.*** *A MESS analysis for models trained on the smaller Gabonese training area projected to the larger tropical African area shows considerable areas with negative MESS values meaning that one or more environmental variables have values outside the range present in the training data (Elith et al., 2010).*

*
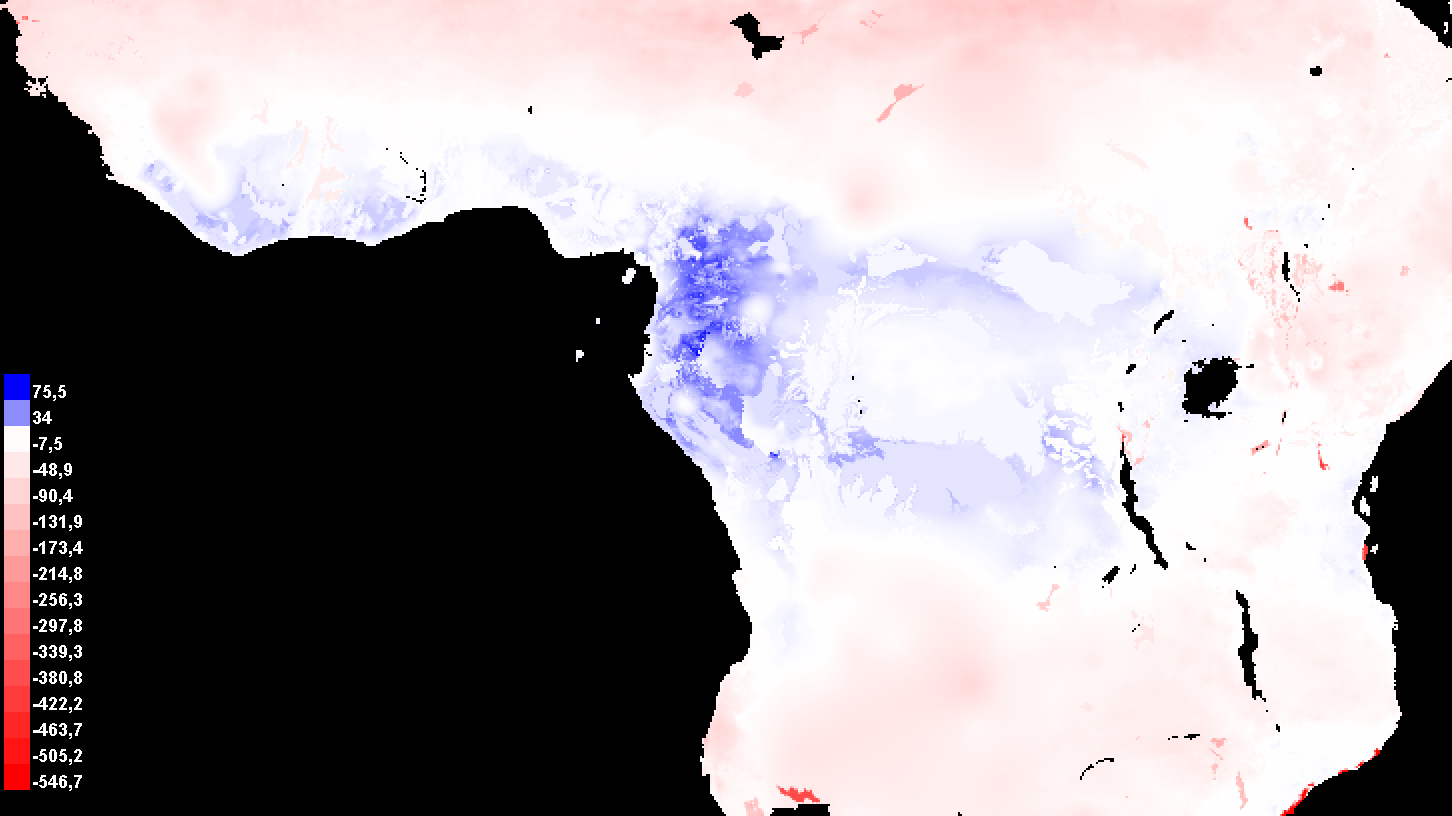
*

## References

- *Elith, J., Kearney, M. & Phillips, S. (2010) The art of modelling range-shifting species. Methods in Ecology and Evolution,* ***1****, 330-342.*
