# Supplementary material for: Unequal Contribution of Widespread and Narrow-Ranged Species to Botanical Diversity Patterns
Source: PLoS One. 2016 Dec 29;11(12):e0169200. doi: 10.1371/journal.pone.0169200 (PMC5199077; doi:10.1371/journal.pone.0169200)
Supplement: S3 Fig — The range size frequency distribution of the species with accepted SDMs is shown, with range size or prevalence of the species defined as the fraction of raster cells where the species is predicted to be present in tropical Africa. In black the original RSFD values based on Species Distribution Models trained on either tropical Africa or Gabon and including only species with accepted SDMs that are predicted to be present in Gabon (same as in main text Fig 1). In grey the RSFD values based on SDMs which are all trained on tropical Africa and including all species with accepted SDMs, thus including those species recorded from the five degree buffer zone but predicted to be absent for Gabon itself. (DOCX) [file pone.0169200.s003.docx]

***van Proosdij, A.S.J., Raes, N., Wieringa, J.J. and Sosef, M.S.M. 2016.***

***Title: Unequal contribution of widespread and narrow-ranged species to botanical diversity patterns.***

***Journal: Plos One.***

***Corresponding author: André S.J. van Proosdij,*** [***andrevanproosdij@hotmail.com***](mailto:andrevanproosdij@hotmail.com)

***S3 Fig. Comparison of range size frequency distributions.*** *The range size frequency distribution of the species with accepted SDMs is shown, with range size or prevalence of the species defined as the fraction of raster cells where the species is predicted to be present in tropical Africa. In black the original RSFD values based on Species Distribution Models trained on either tropical Africa or Gabon and including only species with accepted SDMs that are predicted to be present in Gabon (same as in main text Figure 1). In grey the RSFD values based on SDMs which are all trained on tropical Africa and including all species with accepted SDMs, thus including those species recorded from the five degree buffer zone but predicted to be absent for Gabon itself.*

**
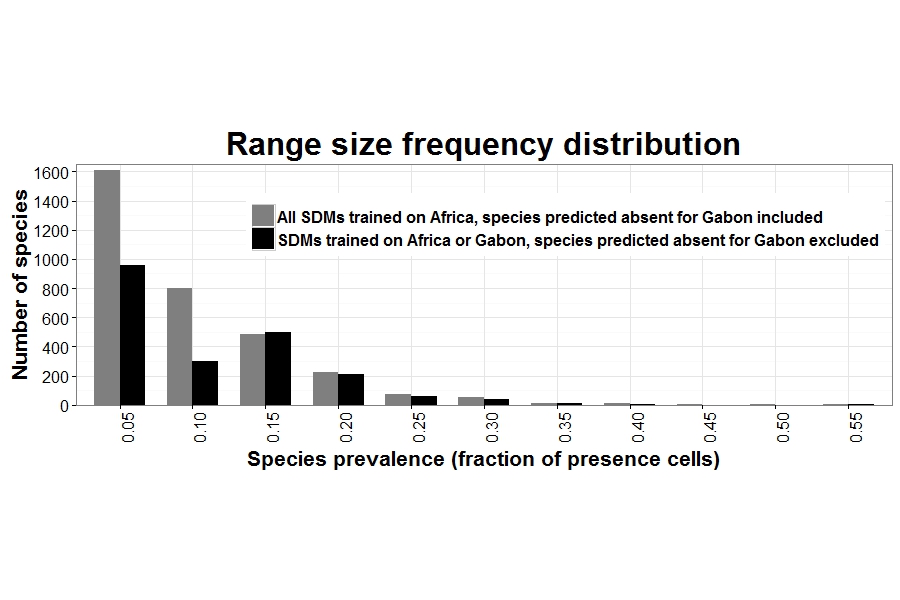
**
