# Supplementary material for: Unequal Contribution of Widespread and Narrow-Ranged Species to Botanical Diversity Patterns
Source: PLoS One. 2016 Dec 29;11(12):e0169200. doi: 10.1371/journal.pone.0169200 (PMC5199077; doi:10.1371/journal.pone.0169200)
Supplement: S1 Table — Variables are selected based on a Spearman’s |rho| < 0.7. Correlated variables are given for each selected variable. (DOCX) [file pone.0169200.s004.docx]

***van Proosdij, A.S.J., Raes, N., Wieringa, J.J. and Sosef, M.S.M. 2016.***

***Title: Unequal contribution of widespread and narrow-ranged species to botanical diversity patterns.***

***Journal: Plos One.***

***Corresponding author: André S.J. van Proosdij,*** [***andrevanproosdij@hotmail.com***](mailto:andrevanproosdij@hotmail.com)

***S1 Table. Selected environmental parameters.*** *Variables are selected based on a Spearman’s |*rho*| < 0.7. Correlated variables are given for each selected variable.*

| **Selected climatic and altitude variables** | **Correlated variables** |
| --- | --- |
| BIO02: Mean Diurnal Temperature Range | - Temperature Annual Range (Max Temperature of Warmest Month - Min Temperature of Coldest Month BIO07) |
| BIO12: Annual Precipitation | - Precipitation of Wettest Month (BIO13) - Precipitation Seasonality (BIO15) - Precipitation of Wettest Quarter (BIO16) - Precipitation of Driest Quarter (BIO17) - Potential Evapotranspiration ratio (PET) |
| BIO15: Precipitation Seasonality | - Isothermality (BIO03) - Temperature Seasonality (BIO04) - Temperature Annual Range (Max Temperature of Warmest Month - Min Temperature of Coldest Month BIO07) - Precipitation of Driest Month (BIO14) - Precipitation of Driest Quarter (BIO17) - Precipitation of Coldest Quarter (BIO19) |
| BIO18: Precipitation of Warmest Quarter | - Max Temperature of Warmest Month (BIO05) - Annual Precipitation (BIO12) |
| ALT: Altitude | - Annual Mean Temperature (BIO01) - Min Temperature of Coldest Month (BIO06) - Mean Temperature of Wettest Quarter (BIO08) - Mean Temperature of Driest Quarter (BIO09) - Mean Temperature of Warmest Quarter (BIO10) - Mean Temperature of Coldest Quarter (BIO11) |
| DEM-SD: Standard Deviation of Elevation | - Elevation Range (DEM-MAXMIN) |
| **Selected soil variables** | **Correlated variables** |
| AWC_CLASS: Available Water Storage Capacity |  |
| DRAINAGE: Drainage Class |  |
| REF_DEPTH: Reference Depth of the soil |  |
| T_BULK_DENSITY: Topsoil Bulk Density |  |
| T_ESP: Topsoil Sodicity |  |
| T_GRAVEL: Topsoil Gravel Content |  |
| T_OC: Topsoil Organic Carbon |  |
| T_PH_H2O: Topsoil pH | - Topsoil Base Saturation (T-BS) - Topsoil Calcium Carbonate (T_CACO3) - Topsoil Total Exchangeable Bases (T_TEB) |
| T_SAND: Topsoil Sand Fraction | - Topsoil Cation Exchange Capacity entire soil (T-CEC-SOIL) - Topsoil Clay Fraction (T-CLAY) - Topsoil Silt Fraction (T-SILT) - Topsoil Texture (T-TEXTURE) |
