# Supplementary material for: Unequal Contribution of Widespread and Narrow-Ranged Species to Botanical Diversity Patterns
Source: PLoS One. 2016 Dec 29;11(12):e0169200. doi: 10.1371/journal.pone.0169200 (PMC5199077; doi:10.1371/journal.pone.0169200)
Supplement: S2 Table — For each of the 2089 species that are predicted present in Gabon based on significant Species Distribution Models, the scientific name, family name and higher taxonomic rank are given. (DOCX) [file pone.0169200.s005.docx]

***van Proosdij, A.S.J., Raes, N., Wieringa, J.J. and Sosef, M.S.M. 2016.***

***Title: Unequal contribution of widespread and narrow-ranged species to botanical diversity patterns.***

***Journal: Plos One.***

***Corresponding author: André S.J. van Proosdij,*** [***andrevanproosdij@hotmail.com***](mailto:andrevanproosdij@hotmail.com)

***S2 Table. List of species predicted present in Gabon.*** *For each of the 2089 species that are predicted present in Gabon based on significant Species Distribution Models, the scientific name, family name and higher taxonomic rank are given.*

| **Species** | **Family** | **Class** |
| --- | --- | --- |
| Rhacopilopsis trinitensis (Müll.Hal.) E.Britton & Dixon | Bryophyta | Musci |
| Leucomium strumosum (Hornsch.) Mitt. | Musci | Musci |
| Pelekium gratum (P.Beauv.) Touw | Musci | Musci |
| Pelekium involvens (Hedw.) Touw | Musci | Musci |
| Callicostella brevipes (Broth.) Broth. | Musci-Calymperaceae | Musci |
| Callicostella chevalieri Broth. | Musci-Calymperaceae | Musci |
| Calymperes erosum Müll.Hal. | Musci-Calymperaceae | Musci |
| Calymperes lonchophyllum Schwägr. | Musci-Calymperaceae | Musci |
| Leucophanes molleri Müll.Hal. | Musci-Calymperaceae | Musci |
| Octoblepharum albidum Hedw. | Musci-Calymperaceae | Musci |
| Syrrhopodon armatus Mitt. | Musci-Calymperaceae | Musci |
| Fissidens marthae Cardot | Musci-Fissidentaceae | Musci |
| Fissidens pellucidus Hornsch. | Musci-Fissidentaceae | Musci |
| Fissidens porrectus Mitt. | Musci-Fissidentaceae | Musci |
| Fissidens ramulosus Mitt. | Musci-Fissidentaceae | Musci |
| Fissidens sciophyllus Mitt. | Musci-Fissidentaceae | Musci |
| Cyclodictyon krebedjense Broth. | Musci-Hookeriaceae | Musci |
| Isopterygium gabonense Broth. & P.de la Varde | Musci-Hypnaceae | Musci |
| Vesicularia oreadelphus (Broth.) Broth. | Musci-Hypnaceae | Musci |
| Pinnatella minuta (Mitt.) Broth. | Musci-Neckeraceae | Musci |
| Taxithelium planum (Brid.) Mitt. | Musci-Sematophyllaceae | Musci |
| Taxithelium ramivagum Broth. | Musci-Sematophyllaceae | Musci |
| Lepidozia succida Mitt. | Hepaticae | Marchantiomorpha |
| Huperzia dacrydioides (Baker) Pic.Serm. | Lycopodiaceae | Lycopsida |
| Huperzia ophioglossoides (Lam.) Rothm. | Lycopodiaceae | Lycopsida |
| Selaginella cathedrifolia Spring | Selaginellaceae | Lycopsida |
| Selaginella grallipes Alston | Selaginellaceae | Lycopsida |
| Selaginella kraussiana (Kunze) A.Braun | Selaginellaceae | Lycopsida |
| Selaginella myosurus (Sw.) Alston | Selaginellaceae | Lycopsida |
| Selaginella versicolor Spring | Selaginellaceae | Lycopsida |
| Selaginella vogelii Spring | Selaginellaceae | Lycopsida |
| Adiantum philippense L. | Adiantaceae | Pteropsida |
| Pellaea dura (Willd.) Hook. | Adiantaceae | Pteropsida |
| Arthropteris cameroonensis Alston | Arthropteridaceae | Pteropsida |
| Arthropteris monocarpa (Cordem.) C.Chr. | Arthropteridaceae | Pteropsida |
| Asplenium africanum Desv. | Aspleniaceae | Pteropsida |
| Asplenium anisophyllum Kunze | Aspleniaceae | Pteropsida |
| Asplenium annetii (Jeanp.) Alston | Aspleniaceae | Pteropsida |
| Asplenium barteri Hook. | Aspleniaceae | Pteropsida |
| Asplenium dregeanum Kunze | Aspleniaceae | Pteropsida |
| Asplenium formosum Willd. | Aspleniaceae | Pteropsida |
| Asplenium hallei Tardieu | Aspleniaceae | Pteropsida |
| Asplenium hemitomum Hieron. | Aspleniaceae | Pteropsida |
| Asplenium jaundeense Hieron. | Aspleniaceae | Pteropsida |
| Asplenium macrophlebium Baker | Aspleniaceae | Pteropsida |
| Asplenium paucijugum Ballard | Aspleniaceae | Pteropsida |
| Asplenium sandersonii Hook. | Aspleniaceae | Pteropsida |
| Asplenium staudtii Hieron. | Aspleniaceae | Pteropsida |
| Asplenium theciferum (Kunth) Mett. | Aspleniaceae | Pteropsida |
| Asplenium unilaterale Lam. | Aspleniaceae | Pteropsida |
| Alsophila camerooniana (Hook.) R.M.Tryon | Cyatheaceae | Pteropsida |
| Cyathea manniana Hook. | Cyatheaceae | Pteropsida |
| Humata repens (L.f.) J.Small ex Diels | Davalliaceae | Pteropsida |
| Blotiella crenata (Alston) Schelpe | Dennstaedtiaceae | Pteropsida |
| Blotiella mannii (Baker) Pic.Serm. | Dennstaedtiaceae | Pteropsida |
| Dryopteris concolor (Langsd. & Fisch.) Kuhn | Dryopteridaceae | Pteropsida |
| Dryopteris inaequalis (Schltdl.) Kuntze | Dryopteridaceae | Pteropsida |
| Dryopteris kilemensis (Kuhn) Kuntze | Dryopteridaceae | Pteropsida |
| Megalastrum lanuginosum (Willd. ex Kaulf.) Holttum | Dryopteridaceae | Pteropsida |
| Tectaria angelicifolia (Schumach.) Copel. | Dryopteridaceae | Pteropsida |
| Tectaria barteri (J.Sm.) C.Chr. | Dryopteridaceae | Pteropsida |
| Triplophyllum buchholzii (Kuhn) Holttum | Dryopteridaceae | Pteropsida |
| Triplophyllum dimidiatum (Mett. ex Kuhn) Holttum | Dryopteridaceae | Pteropsida |
| Triplophyllum jenseniae (C.Chr.) Holttum | Dryopteridaceae | Pteropsida |
| Triplophyllum protensum (Afzel. ex Sw.) Holttum | Dryopteridaceae | Pteropsida |
| Triplophyllum varians (T.Moore) Holttum | Dryopteridaceae | Pteropsida |
| Cochlidium serrulatum (Sw.) L.E.Bishop | Grammitidaceae | Pteropsida |
| Grammitis nigrocincta Alston | Grammitidaceae | Pteropsida |
| Abrodictyum cupressoides (Desv.) Ebihara & Dubuisson | Hymenophyllaceae | Pteropsida |
| Didymoglossum erosum (Willd.) J.P.Roux | Hymenophyllaceae | Pteropsida |
| Hymenophyllum hirsutum (L.) Sw. | Hymenophyllaceae | Pteropsida |
| Hymenophyllum kuhnii C.Chr. | Hymenophyllaceae | Pteropsida |
| Hymenophyllum triangulare Baker | Hymenophyllaceae | Pteropsida |
| Trichomanes crispiforme Alston | Hymenophyllaceae | Pteropsida |
| Bolbitis gaboonensis (Hook.) Alston | Lomariopsidaceae | Pteropsida |
| Elaphoglossum isabelense Brause | Lomariopsidaceae | Pteropsida |
| Lomariopsis guineensis (Underw.) Alston | Lomariopsidaceae | Pteropsida |
| Lomariopsis hederacea Alston | Lomariopsidaceae | Pteropsida |
| Loxogramme abyssinica (Baker) M.G.Price | Loxogrammaceae | Pteropsida |
| Marattia fraxinea Sm. | Marattiaceae | Pteropsida |
| Nephrolepis biserrata (Sw.) Schott | Oleandraceae | Pteropsida |
| Oleandra distenta Kunze | Oleandraceae | Pteropsida |
| Osmunda regalis L. | Osmundaceae | Pteropsida |
| Drynaria laurentii (Christ) Hieron. | Polypodiaceae | Pteropsida |
| Drynaria volkensii Hieron. | Polypodiaceae | Pteropsida |
| Microgramma lycopodioides (L.) Copel. | Polypodiaceae | Pteropsida |
| Microsorum punctatum (L.) Copel. | Polypodiaceae | Pteropsida |
| Phymatosorus scolopendria (Burm.f.) Pic.Serm. | Polypodiaceae | Pteropsida |
| Pyrrosia schimperiana (Mett. ex Kuhn) Alston | Polypodiaceae | Pteropsida |
| Acrostichum aureum L. | Pteridaceae | Pteropsida |
| Pteris manniana Mett. ex Kuhn | Pteridaceae | Pteropsida |
| Pteris muricella Fée | Pteridaceae | Pteropsida |
| Amauropelta bergiana (Schltdl.) Holttum | Thelypteridaceae | Pteropsida |
| Pneumatopteris afra (Christ) Holttum | Thelypteridaceae | Pteropsida |
| Gnetum africanum Welw. | Gnetaceae | Pinopsida |
| Acanthus latisepalus C.B.Clarke | Acanthaceae | Magnoliopsida |
| Acanthus montanus (Nees) T.Anderson | Acanthaceae | Magnoliopsida |
| Adhatoda buchholzii (Lindau) S.Moore | Acanthaceae | Magnoliopsida |
| Adhatoda tristis Nees | Acanthaceae | Magnoliopsida |
| Afrofittonia silvestris Lindau | Acanthaceae | Magnoliopsida |
| Anisosepalum alboviolaceum (Benoist) E.Hossain | Acanthaceae | Magnoliopsida |
| Anisotes macrophyllus (Lindau) Heine | Acanthaceae | Magnoliopsida |
| Ascotheca paucinervia (T.Anderson ex C.B.Clarke) Heine | Acanthaceae | Magnoliopsida |
| Asystasia leptostachya Lindau | Acanthaceae | Magnoliopsida |
| Asystasia lindauiana Hutch. & Dalziel | Acanthaceae | Magnoliopsida |
| Asystasia macrophylla (T.Anderson) Benth. ex Lindau | Acanthaceae | Magnoliopsida |
| Asystasia vogeliana Benth. | Acanthaceae | Magnoliopsida |
| Avicennia germinans (L.) L. | Acanthaceae | Magnoliopsida |
| Brachystephanus jaundensis Lindau | Acanthaceae | Magnoliopsida |
| Brillantaisia debilis Burkill | Acanthaceae | Magnoliopsida |
| Brillantaisia lamium (Nees) Benth. | Acanthaceae | Magnoliopsida |
| Brillantaisia owariensis P.Beauv. | Acanthaceae | Magnoliopsida |
| Brillantaisia vogeliana (Nees) Benth. | Acanthaceae | Magnoliopsida |
| Dischistocalyx grandifolius C.B.Clarke | Acanthaceae | Magnoliopsida |
| Dischistocalyx hirsutus C.B.Clarke | Acanthaceae | Magnoliopsida |
| Dischistocalyx strobilinus C.B.Clarke | Acanthaceae | Magnoliopsida |
| Elytraria marginata Vahl | Acanthaceae | Magnoliopsida |
| Filetia africana Lindau | Acanthaceae | Magnoliopsida |
| Justicia extensa T.Anderson | Acanthaceae | Magnoliopsida |
| Justicia laxa T.Anderson | Acanthaceae | Magnoliopsida |
| Justicia paxiana Lindau | Acanthaceae | Magnoliopsida |
| Justicia preussii (Lindau) C.B.Clarke | Acanthaceae | Magnoliopsida |
| Justicia tigrina Heine | Acanthaceae | Magnoliopsida |
| Lankesteria brevior C.B.Clarke | Acanthaceae | Magnoliopsida |
| Lankesteria elegans (P.Beauv.) T.Anderson | Acanthaceae | Magnoliopsida |
| Mendoncia gilgiana (Lindau) Benoist | Acanthaceae | Magnoliopsida |
| Mendoncia lindaviana (Gilg) Benoist | Acanthaceae | Magnoliopsida |
| Monechma depauperatum (T.Anderson) C.B.Clarke | Acanthaceae | Magnoliopsida |
| Phaulopsis angolana S.Moore | Acanthaceae | Magnoliopsida |
| Physacanthus batanganus (J.Braun & K.Schum.) Lindau | Acanthaceae | Magnoliopsida |
| Physacanthus cylindricus C.B.Clarke | Acanthaceae | Magnoliopsida |
| Pseuderanthemum ludovicianum (Büttner) Lindau | Acanthaceae | Magnoliopsida |
| Pseuderanthemum tunicatum (Afzel.) Milne-Redh. | Acanthaceae | Magnoliopsida |
| Ruellia primuloides (T.Anderson ex Benth.) Heine | Acanthaceae | Magnoliopsida |
| Rungia buettneri Lindau | Acanthaceae | Magnoliopsida |
| Rungia camerunensis Champl. | Acanthaceae | Magnoliopsida |
| Saintpauliopsis lebrunii Staner | Acanthaceae | Magnoliopsida |
| Schaueria populifolia C.B.Clarke | Acanthaceae | Magnoliopsida |
| Staurogyne bicolor (Mildbr.) Champl. | Acanthaceae | Magnoliopsida |
| Staurogyne letestuana Benoist | Acanthaceae | Magnoliopsida |
| Stenandrium gabonicum (Benoist) Vollesen | Acanthaceae | Magnoliopsida |
| Stenandrium talbotii (S.Moore) Vollesen | Acanthaceae | Magnoliopsida |
| Thunbergia alata Bojer ex Sims | Acanthaceae | Magnoliopsida |
| Whitfieldia brazzae (Baill.) C.B.Clarke | Acanthaceae | Magnoliopsida |
| Whitfieldia elongata (P.Beauv.) De Wild. & T.Durand | Acanthaceae | Magnoliopsida |
| Whitfieldia le-testui Benoist | Acanthaceae | Magnoliopsida |
| Whitfieldia preussii (Lindau) C.B.Clarke | Acanthaceae | Magnoliopsida |
| Sesuvium portulacastrum (L.) L. | Aizoaceae | Magnoliopsida |
| Alangium chinense (Lour.) Harms | Alangiaceae | Magnoliopsida |
| Limnophyton fluitans Graebn. | Alismataceae | Magnoliopsida |
| Alternanthera maritima (Mart.) A.St.-Hil. | Amaranthaceae | Magnoliopsida |
| Blutaparon vermiculare (L.) Mears | Amaranthaceae | Magnoliopsida |
| Pandiaka angustifolia (Vahl) Hepper | Amaranthaceae | Magnoliopsida |
| Sericostachys scandens Gilg & Lopr. | Amaranthaceae | Magnoliopsida |
| Scadoxus cinnabarinus (Decne.) Friis & Nordal | Amaryllidaceae | Magnoliopsida |
| Fegimanra africana (Oliv.) Pierre | Anacardiaceae | Magnoliopsida |
| Haematostaphis barteri Hook.f. | Anacardiaceae | Magnoliopsida |
| Ozoroa pulcherrima (Schweinf.) R.Fern. & A.Fern. | Anacardiaceae | Magnoliopsida |
| Sorindeia africana (Engl.) Van der Veken | Anacardiaceae | Magnoliopsida |
| Sorindeia gabonensis Bourobou & Breteler | Anacardiaceae | Magnoliopsida |
| Trichoscypha bijuga Engl. | Anacardiaceae | Magnoliopsida |
| Trichoscypha imbricata Engl. | Anacardiaceae | Magnoliopsida |
| Trichoscypha laxiflora Engl. | Anacardiaceae | Magnoliopsida |
| Trichoscypha mannii Hook.f. | Anacardiaceae | Magnoliopsida |
| Ancistrocladus ealaensis J.Léonard | Ancistrocladaceae | Magnoliopsida |
| Anisophyllea polyneura Floret | Anisophylleaceae | Magnoliopsida |
| Anisophyllea purpurascens Hutch. & Dalziel | Anisophylleaceae | Magnoliopsida |
| Anisophyllea quangensis Engl. ex Henriq. | Anisophylleaceae | Magnoliopsida |
| Annickia lebrunii (Robyns & Ghesq.) Setten & Maas | Annonaceae | Magnoliopsida |
| Annickia pilosa (Exell) Setten & Maas | Annonaceae | Magnoliopsida |
| Annona glabra L. | Annonaceae | Magnoliopsida |
| Anonidium floribundum Pellegr. | Annonaceae | Magnoliopsida |
| Anonidium mannii (Oliv.) Engl. & Diels | Annonaceae | Magnoliopsida |
| Artabotrys congolensis De Wild. & T.Durand | Annonaceae | Magnoliopsida |
| Artabotrys pierreanus Engl. & Diels | Annonaceae | Magnoliopsida |
| Artabotrys thomsonii Oliv. | Annonaceae | Magnoliopsida |
| Boutiquea platypetala (Engl. & Diels) Le Thomas | Annonaceae | Magnoliopsida |
| Cleistopholis glauca Pierre ex Engl. & Diels | Annonaceae | Magnoliopsida |
| Cleistopholis patens (Benth.) Engl. & Diels | Annonaceae | Magnoliopsida |
| Duguetia confinis (Engl. & Diels) Chatrou | Annonaceae | Magnoliopsida |
| Duguetia staudtii (Engl. & Diels) Chatrou | Annonaceae | Magnoliopsida |
| Friesodielsia enghiana (Diels) Verdc. | Annonaceae | Magnoliopsida |
| Friesodielsia montana (Engl. & Diels) Steenis | Annonaceae | Magnoliopsida |
| Greenwayodendron suaveolens (Engl. & Diels) Verdc. | Annonaceae | Magnoliopsida |
| Isolona campanulata Engl. & Diels | Annonaceae | Magnoliopsida |
| Isolona hexaloba (Pierre) Engl. | Annonaceae | Magnoliopsida |
| Isolona pilosa Diels | Annonaceae | Magnoliopsida |
| Isolona thonneri (De Wild. & T.Durand) Engl. & Diels | Annonaceae | Magnoliopsida |
| Isolona zenkeri Engl. | Annonaceae | Magnoliopsida |
| Meiocarpidium lepidotum (Oliv.) Engl. & Diels | Annonaceae | Magnoliopsida |
| Monanthotaxis cauliflora (Chipp) Verdc. | Annonaceae | Magnoliopsida |
| Monanthotaxis congoensis Baill. | Annonaceae | Magnoliopsida |
| Monanthotaxis diclina (Sprague) Verdc. | Annonaceae | Magnoliopsida |
| Monanthotaxis ferruginea (Oliv.) Verdc. | Annonaceae | Magnoliopsida |
| Monanthotaxis glomerulata (Le Thomas) Verdc. | Annonaceae | Magnoliopsida |
| Monanthotaxis klainei (Pierre ex Engl. & Diels) Verdc. | Annonaceae | Magnoliopsida |
| Monanthotaxis le-testui Pellegr. | Annonaceae | Magnoliopsida |
| Monanthotaxis lucidula (Oliv.) Verdc. | Annonaceae | Magnoliopsida |
| Monanthotaxis poggei Engl. & Diels | Annonaceae | Magnoliopsida |
| Monodora angolensis Welw. | Annonaceae | Magnoliopsida |
| Monodora crispata Engl. | Annonaceae | Magnoliopsida |
| Monodora laurentii De Wild. | Annonaceae | Magnoliopsida |
| Monodora tenuifolia Benth. | Annonaceae | Magnoliopsida |
| Monodora undulata (P.Beauv.) Couvreur | Annonaceae | Magnoliopsida |
| Neostenanthera gabonensis (Engl. & Diels) Exell | Annonaceae | Magnoliopsida |
| Neostenanthera robsonii Le Thomas | Annonaceae | Magnoliopsida |
| Piptostigma longipilosum Engl. | Annonaceae | Magnoliopsida |
| Piptostigma macrophyllum Ghogue, Sonké & Couvreur | Annonaceae | Magnoliopsida |
| Piptostigma multinervium Engl. & Diels | Annonaceae | Magnoliopsida |
| Polyceratocarpus parviflorus (Baker f.) Ghesq. | Annonaceae | Magnoliopsida |
| Pseudartabotrys le-testui Pellegr. | Annonaceae | Magnoliopsida |
| Uvaria clavata Pierre ex Engl. & Diels | Annonaceae | Magnoliopsida |
| Uvaria comperei Le Thomas | Annonaceae | Magnoliopsida |
| Uvaria klaineana Engl. & Diels | Annonaceae | Magnoliopsida |
| Uvaria klainei Pierre ex Engl. & Diels | Annonaceae | Magnoliopsida |
| Uvaria versicolor Pierre ex Engl. & Diels | Annonaceae | Magnoliopsida |
| Uvariastrum germainii Boutique | Annonaceae | Magnoliopsida |
| Uvariastrum pierreanum Engl. & Diels | Annonaceae | Magnoliopsida |
| Uvariastrum zenkeri Engl. & Diels | Annonaceae | Magnoliopsida |
| Uvariodendron calophyllum R.E.Fr. | Annonaceae | Magnoliopsida |
| Uvariodendron connivens (Benth.) R.E.Fr. | Annonaceae | Magnoliopsida |
| Uvariopsis korupensis Gereau & Kenfack | Annonaceae | Magnoliopsida |
| Uvariopsis submontana Kenfack, Gosline & Gereau | Annonaceae | Magnoliopsida |
| Xylopia aethiopica (Dunal) A.Rich. | Annonaceae | Magnoliopsida |
| Xylopia africana (Benth.) Oliv. | Annonaceae | Magnoliopsida |
| Xylopia aurantiiodora De Wild. & T.Durand | Annonaceae | Magnoliopsida |
| Xylopia le-testui Pellegr. | Annonaceae | Magnoliopsida |
| Alafia barteri Oliv. | Apocynaceae | Magnoliopsida |
| Alstonia congensis Engl. | Apocynaceae | Magnoliopsida |
| Ancylobotrys scandens (Schumach. & Thonn.) Pichon | Apocynaceae | Magnoliopsida |
| Baissea gracillima (K.Schum.) Hua | Apocynaceae | Magnoliopsida |
| Baissea leonensis Benth. | Apocynaceae | Magnoliopsida |
| Baissea multiflora A.DC. | Apocynaceae | Magnoliopsida |
| Baissea subrufa Stapf | Apocynaceae | Magnoliopsida |
| Callichilia barteri (Hook.f.) Stapf | Apocynaceae | Magnoliopsida |
| Callichilia bequaertii De Wild. | Apocynaceae | Magnoliopsida |
| Callichilia inaequalis Stapf | Apocynaceae | Magnoliopsida |
| Calocrater preussii K.Schum. | Apocynaceae | Magnoliopsida |
| Crioceras dipladeniiflorus (Stapf) K.Schum. | Apocynaceae | Magnoliopsida |
| Cylindropsis parvifolia Pierre | Apocynaceae | Magnoliopsida |
| Dictyophleba ochracea (K.Schum. ex Hallier f.) Pichon | Apocynaceae | Magnoliopsida |
| Dictyophleba stipulosa (S.Moore ex Wernham) Pichon | Apocynaceae | Magnoliopsida |
| Farquharia elliptica Stapf | Apocynaceae | Magnoliopsida |
| Funtumia africana (Benth.) Stapf | Apocynaceae | Magnoliopsida |
| Funtumia elastica (P.Preuss) Stapf | Apocynaceae | Magnoliopsida |
| Hunteria ballayi Hua | Apocynaceae | Magnoliopsida |
| Hunteria oxyantha Omino | Apocynaceae | Magnoliopsida |
| Hunteria umbellata (K.Schum.) Hallier f. | Apocynaceae | Magnoliopsida |
| Landolphia breviloba J.G.M.Pers. | Apocynaceae | Magnoliopsida |
| Landolphia bruneelii (De Wild.) Pichon | Apocynaceae | Magnoliopsida |
| Landolphia buchananii (Hallier f.) Stapf | Apocynaceae | Magnoliopsida |
| Landolphia dewevrei Stapf | Apocynaceae | Magnoliopsida |
| Landolphia foretiana (Pierre ex Jum.) Pichon | Apocynaceae | Magnoliopsida |
| Landolphia incerta (K.Schum.) J.G.M.Pers. | Apocynaceae | Magnoliopsida |
| Landolphia lecomtei Dewèvre | Apocynaceae | Magnoliopsida |
| Landolphia leptantha (K.Schum.) J.G.M.Pers. | Apocynaceae | Magnoliopsida |
| Landolphia ligustrifolia (Stapf) Pichon | Apocynaceae | Magnoliopsida |
| Landolphia mannii Dyer | Apocynaceae | Magnoliopsida |
| Landolphia reticulata Hallier f. | Apocynaceae | Magnoliopsida |
| Malouetia bequaertiana Woodson | Apocynaceae | Magnoliopsida |
| Motandra lujaei De Wild. & T.Durand | Apocynaceae | Magnoliopsida |
| Oncinotis glabrata (Baill.) Stapf ex Hiern | Apocynaceae | Magnoliopsida |
| Oncinotis pontyi Dubard | Apocynaceae | Magnoliopsida |
| Picralima nitida (Stapf) T.Durand & H.Durand | Apocynaceae | Magnoliopsida |
| Pleiocarpa bicarpellata Stapf | Apocynaceae | Magnoliopsida |
| Pleiocarpa brevistyla Omino | Apocynaceae | Magnoliopsida |
| Pleiocarpa mutica Benth. | Apocynaceae | Magnoliopsida |
| Pleiocarpa rostrata Benth. | Apocynaceae | Magnoliopsida |
| Rauvolfia letouzeyi Leeuwenb. | Apocynaceae | Magnoliopsida |
| Rauvolfia mannii Stapf | Apocynaceae | Magnoliopsida |
| Rauvolfia vomitoria Afzel. | Apocynaceae | Magnoliopsida |
| Strophanthus gratus (Wall. & Hook.) Baill. | Apocynaceae | Magnoliopsida |
| Strophanthus thollonii Franch. | Apocynaceae | Magnoliopsida |
| Tabernaemontana contorta Stapf | Apocynaceae | Magnoliopsida |
| Tabernaemontana crassa Benth. | Apocynaceae | Magnoliopsida |
| Tabernaemontana eglandulosa Stapf | Apocynaceae | Magnoliopsida |
| Tabernaemontana hallei (Boiteau) Leeuwenb. | Apocynaceae | Magnoliopsida |
| Tabernaemontana inconspicua Stapf | Apocynaceae | Magnoliopsida |
| Tabernaemontana letestui (Pellegr.) Pichon | Apocynaceae | Magnoliopsida |
| Tabernaemontana penduliflora K.Schum. | Apocynaceae | Magnoliopsida |
| Tabernanthe iboga Baill. | Apocynaceae | Magnoliopsida |
| Vahadenia laurentii (De Wild.) Stapf | Apocynaceae | Magnoliopsida |
| Voacanga bracteata Stapf | Apocynaceae | Magnoliopsida |
| Anubias barteri Schott | Araceae | Magnoliopsida |
| Cercestis kamerunianus (Engl.) N.E.Br. | Araceae | Magnoliopsida |
| Culcasia lancifolia N.E.Br. | Araceae | Magnoliopsida |
| Culcasia mannii (Hook.f.) Engl. | Araceae | Magnoliopsida |
| Culcasia panduriformis Engl. & K.Krause | Araceae | Magnoliopsida |
| Culcasia parviflora N.E.Br. | Araceae | Magnoliopsida |
| Culcasia rotundifolia Bogner | Araceae | Magnoliopsida |
| Culcasia saxatilis A.Chev. | Araceae | Magnoliopsida |
| Culcasia seretii De Wild. | Araceae | Magnoliopsida |
| Culcasia striolata Engl. | Araceae | Magnoliopsida |
| Culcasia tenuifolia Engl. | Araceae | Magnoliopsida |
| Lasimorpha senegalensis Schott | Araceae | Magnoliopsida |
| Nephthytis afzelii Schott | Araceae | Magnoliopsida |
| Nephthytis poissonii (Engl.) N.E.Br. | Araceae | Magnoliopsida |
| Pseudohydrosme gabunensis Engl. | Araceae | Magnoliopsida |
| Rhaphidophora africana N.E.Br. | Araceae | Magnoliopsida |
| Stylochaeton zenkeri Engl. | Araceae | Magnoliopsida |
| Cynanchum adalinae (K.Schum.) K.Schum. | Asclepiadaceae | Magnoliopsida |
| Epistemma rupestre H.Huber | Asclepiadaceae | Magnoliopsida |
| Parquetina nigrescens (Afzel.) Bullock | Asclepiadaceae | Magnoliopsida |
| Secamone afzelii (Schult.) K.Schum. | Asclepiadaceae | Magnoliopsida |
| Tylophora congolana (Baill.) Bullock | Asclepiadaceae | Magnoliopsida |
| Xysmalobium holubii Scott-Elliot | Asclepiadaceae | Magnoliopsida |
| Chlorophytum alismifolium Baker | Asparagaceae | Magnoliopsida |
| Chlorophytum blepharophyllum Schweinf. ex Baker | Asparagaceae | Magnoliopsida |
| Chlorophytum orchidastrum Lindl. | Asparagaceae | Magnoliopsida |
| Chlorophytum petrophilum K.Krause | Asparagaceae | Magnoliopsida |
| Chlorophytum sparsiflorum Baker | Asparagaceae | Magnoliopsida |
| Dracaena acutissima Hua | Asparagaceae | Magnoliopsida |
| Dracaena aubryana Brongn. ex E.Morren | Asparagaceae | Magnoliopsida |
| Dracaena bicolor Hook. | Asparagaceae | Magnoliopsida |
| Dracaena braunii Engl. | Asparagaceae | Magnoliopsida |
| Dracaena camerooniana Baker | Asparagaceae | Magnoliopsida |
| Dracaena deremensis Engl. | Asparagaceae | Magnoliopsida |
| Dracaena fragrans (L.) Ker Gawl. | Asparagaceae | Magnoliopsida |
| Dracaena glomerata Baker | Asparagaceae | Magnoliopsida |
| Dracaena goldieana Hort. ex Mast. & Moore | Asparagaceae | Magnoliopsida |
| Dracaena haemanthioides Bos ined. | Asparagaceae | Magnoliopsida |
| Dracaena humilis Baker | Asparagaceae | Magnoliopsida |
| Dracaena laxissima Engl. | Asparagaceae | Magnoliopsida |
| Dracaena marina Bos ined. | Asparagaceae | Magnoliopsida |
| Dracaena mildbraedii K.Krause | Asparagaceae | Magnoliopsida |
| Dracaena phanerophlebia Baker | Asparagaceae | Magnoliopsida |
| Dracaena phrynioides Hook. | Asparagaceae | Magnoliopsida |
| Dracaena poggei Engl. | Asparagaceae | Magnoliopsida |
| Dracaena tholloniana Hua | Asparagaceae | Magnoliopsida |
| Dracaena viridiflora Engl. & K.Krause | Asparagaceae | Magnoliopsida |
| Aloe buettneri A.Berger | Asphodelaceae | Magnoliopsida |
| Thonningia sanguinea Vahl | Balanophoraceae | Magnoliopsida |
| Impatiens bipindensis Gilg | Balsaminaceae | Magnoliopsida |
| Impatiens filicornu Hook.f. | Balsaminaceae | Magnoliopsida |
| Impatiens gossweileri G.M.Schulze | Balsaminaceae | Magnoliopsida |
| Impatiens hians Hook.f. | Balsaminaceae | Magnoliopsida |
| Impatiens mackeyana Hook.f. | Balsaminaceae | Magnoliopsida |
| Impatiens macroptera Hook.f. | Balsaminaceae | Magnoliopsida |
| Impatiens mannii Hook.f. | Balsaminaceae | Magnoliopsida |
| Impatiens niamniamensis Gilg | Balsaminaceae | Magnoliopsida |
| Impatiens oumina N.Hallé | Balsaminaceae | Magnoliopsida |
| Impatiens palpebrata Hook.f. | Balsaminaceae | Magnoliopsida |
| Impatiens pseudomacroptera Grey-Wilson | Balsaminaceae | Magnoliopsida |
| Begonia adpressa Sosef | Begoniaceae | Magnoliopsida |
| Begonia ampla Hook.f. | Begoniaceae | Magnoliopsida |
| Begonia anisosepala Hook.f. | Begoniaceae | Magnoliopsida |
| Begonia aspleniifolia Hook.f. | Begoniaceae | Magnoliopsida |
| Begonia atroglandulosa Sosef | Begoniaceae | Magnoliopsida |
| Begonia auriculata Hook.f. | Begoniaceae | Magnoliopsida |
| Begonia bonus-henricus J.J.de Wilde | Begoniaceae | Magnoliopsida |
| Begonia ciliobracteata Warb. | Begoniaceae | Magnoliopsida |
| Begonia clypeifolia Hook.f. | Begoniaceae | Magnoliopsida |
| Begonia elaeagnifolia Hook.f. | Begoniaceae | Magnoliopsida |
| Begonia elatostemmoides Hook.f. | Begoniaceae | Magnoliopsida |
| Begonia eminii Warb. | Begoniaceae | Magnoliopsida |
| Begonia erectocaulis Sosef | Begoniaceae | Magnoliopsida |
| Begonia erectotricha Sosef | Begoniaceae | Magnoliopsida |
| Begonia ferramica N.Hallé | Begoniaceae | Magnoliopsida |
| Begonia fusialata Warb. | Begoniaceae | Magnoliopsida |
| Begonia gabonensis J.J.de Wilde | Begoniaceae | Magnoliopsida |
| Begonia gossweileri Irmsch. | Begoniaceae | Magnoliopsida |
| Begonia heterochroma Sosef | Begoniaceae | Magnoliopsida |
| Begonia hirsutula Hook.f. | Begoniaceae | Magnoliopsida |
| Begonia iucunda Irmsch. | Begoniaceae | Magnoliopsida |
| Begonia karperi J.C.Arends | Begoniaceae | Magnoliopsida |
| Begonia kisuluana Büttner | Begoniaceae | Magnoliopsida |
| Begonia komoensis Irmsch. | Begoniaceae | Magnoliopsida |
| Begonia lacunosa Warb. | Begoniaceae | Magnoliopsida |
| Begonia laporteifolia Warb. | Begoniaceae | Magnoliopsida |
| Begonia letestui J.J.de Wilde | Begoniaceae | Magnoliopsida |
| Begonia letouzeyi Sosef | Begoniaceae | Magnoliopsida |
| Begonia longipetiolata Gilg | Begoniaceae | Magnoliopsida |
| Begonia loranthoides Hook.f. | Begoniaceae | Magnoliopsida |
| Begonia macrocarpa Warb. | Begoniaceae | Magnoliopsida |
| Begonia mannii Hook. | Begoniaceae | Magnoliopsida |
| Begonia mildbraedii Gilg | Begoniaceae | Magnoliopsida |
| Begonia minutifolia N.Hallé | Begoniaceae | Magnoliopsida |
| Begonia oxyloba Welw. ex Hook.f. | Begoniaceae | Magnoliopsida |
| Begonia peperomioides Hook.f. | Begoniaceae | Magnoliopsida |
| Begonia poculifera Hook.f. | Begoniaceae | Magnoliopsida |
| Begonia potamophila Gilg | Begoniaceae | Magnoliopsida |
| Begonia prismatocarpa Hook. | Begoniaceae | Magnoliopsida |
| Begonia quadrialata Warb. | Begoniaceae | Magnoliopsida |
| Begonia sciaphila Gilg ex Engl. | Begoniaceae | Magnoliopsida |
| Begonia scutifolia Hook.f. | Begoniaceae | Magnoliopsida |
| Begonia scutulum Hook.f. | Begoniaceae | Magnoliopsida |
| Begonia sessilifolia Hook.f. | Begoniaceae | Magnoliopsida |
| Begonia squamulosa Hook.f. | Begoniaceae | Magnoliopsida |
| Begonia staudtii Gilg | Begoniaceae | Magnoliopsida |
| Begonia subscutata De Wild. | Begoniaceae | Magnoliopsida |
| Begonia susaniae Sosef | Begoniaceae | Magnoliopsida |
| Begonia vankerckhovenii De Wild. | Begoniaceae | Magnoliopsida |
| Begonia vittariifolia N.Hallé | Begoniaceae | Magnoliopsida |
| Begonia zenkeriana L.B.Sm. & Wassh. | Begoniaceae | Magnoliopsida |
| Cordia aurantiaca Baker | Boraginaceae | Magnoliopsida |
| Cordia sinensis Lam. | Boraginaceae | Magnoliopsida |
| Cynoglossum lanceolatum Forssk. | Boraginaceae | Magnoliopsida |
| Burmannia madagascariensis Mart. | Burmanniaceae | Magnoliopsida |
| Gymnosiphon constrictus Maas & H.Maas | Burmanniaceae | Magnoliopsida |
| Gymnosiphon longistylus (Benth.) Hutch. | Burmanniaceae | Magnoliopsida |
| Aucoumea klaineana Pierre | Burseraceae | Magnoliopsida |
| Dacryodes buettneri (Engl.) H.J.Lam | Burseraceae | Magnoliopsida |
| Dacryodes edulis (G.Don) H.J.Lam | Burseraceae | Magnoliopsida |
| Dacryodes igaganga Aubrév. & Pellegr. | Burseraceae | Magnoliopsida |
| Dacryodes klaineana (Pierre) H.J.Lam | Burseraceae | Magnoliopsida |
| Dacryodes le-testui (Pellegr.) H.J.Lam | Burseraceae | Magnoliopsida |
| Dacryodes macrophylla (Oliv.) H.J.Lam | Burseraceae | Magnoliopsida |
| Dacryodes normandii Aubrév. & Pellegr. | Burseraceae | Magnoliopsida |
| Santiria trimera (Oliv.) Aubrév. | Burseraceae | Magnoliopsida |
| Rhipsalis baccifera (J.S.Muell.) Stearn | Cactaceae | Magnoliopsida |
| Dielsantha galeopsoides (Engl. & Diels) E.Wimm. | Campanulaceae | Magnoliopsida |
| Buchholzia coriacea Engl. | Capparaceae | Magnoliopsida |
| Ritchiea aprevaliana (De Wild. & T.Durand) R.Wilczek | Capparaceae | Magnoliopsida |
| Ritchiea simplicifolia Oliv. | Capparaceae | Magnoliopsida |
| Leptaulus congolanus (Baill.) Lobr.-Callen & Villiers | Cardiopteridaceae | Magnoliopsida |
| Leptaulus daphnoides Benth. | Cardiopteridaceae | Magnoliopsida |
| Leptaulus grandifolius Engl. | Cardiopteridaceae | Magnoliopsida |
| Leptaulus holstii (Engl.) Engl. | Cardiopteridaceae | Magnoliopsida |
| Drymaria cordata (L.) Willd. ex Roem. & Schult. | Caryophyllaceae | Magnoliopsida |
| Casuarina equisetifolia L. | Casuarinaceae | Magnoliopsida |
| Cuervea macrophylla (Vahl) R.Wilczek ex N.Hallé | Celastraceae | Magnoliopsida |
| Hippocratea myriantha Oliv. | Celastraceae | Magnoliopsida |
| Loeseneriella apiculata (Welw. ex Oliv.) N.Hallé & R.Wilczek | Celastraceae | Magnoliopsida |
| Loeseneriella clematoides (Loes.) R.Wilczek ex N.Hallé | Celastraceae | Magnoliopsida |
| Loeseneriella crenata (Klotzsch) R.Wilczek ex N.Hallé | Celastraceae | Magnoliopsida |
| Maytenus acuminata (L.f.) Loes. | Celastraceae | Magnoliopsida |
| Maytenus undata (Thunb.) Blakelock | Celastraceae | Magnoliopsida |
| Salacia alata De Wild. | Celastraceae | Magnoliopsida |
| Salacia cornifolia Hook.f. | Celastraceae | Magnoliopsida |
| Salacia diplasia N.Hallé | Celastraceae | Magnoliopsida |
| Salacia erecta (G.Don) Walp. | Celastraceae | Magnoliopsida |
| Salacia hallei Jongkind | Celastraceae | Magnoliopsida |
| Salacia klainei Pierre ex R.Wilczek | Celastraceae | Magnoliopsida |
| Salacia lehmbachii Loes. | Celastraceae | Magnoliopsida |
| Salacia letouzeyana N.Hallé | Celastraceae | Magnoliopsida |
| Salacia loloensis Loes. | Celastraceae | Magnoliopsida |
| Salacia longipes (Oliv.) N.Hallé | Celastraceae | Magnoliopsida |
| Salacia mannii Oliv. | Celastraceae | Magnoliopsida |
| Salacia mayumbensis Exell & Mendonça | Celastraceae | Magnoliopsida |
| Salacia pynaertii De Wild. | Celastraceae | Magnoliopsida |
| Salacia regeliana J.Braun & K.Schum. | Celastraceae | Magnoliopsida |
| Salacia staudtiana Loes. | Celastraceae | Magnoliopsida |
| Salacia togoica Loes. | Celastraceae | Magnoliopsida |
| Salacia whytei Loes. | Celastraceae | Magnoliopsida |
| Simirestis klaineana N.Hallé | Celastraceae | Magnoliopsida |
| Thyrsosalacia nematobrachion Loes. | Celastraceae | Magnoliopsida |
| Wilczekra gabonica Breteler | Celastraceae | Magnoliopsida |
| Centroplacus glaucinus Pierre | Centroplacaceae | Magnoliopsida |
| Afrolicania elaeosperma Mildbr. | Chrysobalanaceae | Magnoliopsida |
| Chrysobalanus icaco L. | Chrysobalanaceae | Magnoliopsida |
| Dactyladenia barteri (Hook.f. ex Oliv.) Prance & F.White | Chrysobalanaceae | Magnoliopsida |
| Dactyladenia bellayana (Baill.) Prance & F.White | Chrysobalanaceae | Magnoliopsida |
| Dactyladenia campestris (Engl.) Prance & F.White | Chrysobalanaceae | Magnoliopsida |
| Dactyladenia chevalieri (De Wild.) Prance & F.White | Chrysobalanaceae | Magnoliopsida |
| Dactyladenia floretii Breteler | Chrysobalanaceae | Magnoliopsida |
| Dactyladenia icondere (Baill.) Prance & F.White | Chrysobalanaceae | Magnoliopsida |
| Dactyladenia jongkindii Breteler | Chrysobalanaceae | Magnoliopsida |
| Dactyladenia pallescens (Baill.) Prance & F.White | Chrysobalanaceae | Magnoliopsida |
| Magnistipula bimarsupiata Letouzey | Chrysobalanaceae | Magnoliopsida |
| Magnistipula butayei De Wild. | Chrysobalanaceae | Magnoliopsida |
| Magnistipula sp.n. aff. bimarsupiata Breteler | Chrysobalanaceae | Magnoliopsida |
| Maranthes chrysophylla (Oliv.) Prance ex F.White | Chrysobalanaceae | Magnoliopsida |
| Maranthes gabunensis (Engl.) Prance | Chrysobalanaceae | Magnoliopsida |
| Maranthes glabra (Oliv.) Prance | Chrysobalanaceae | Magnoliopsida |
| Parinari capensis Harv. | Chrysobalanaceae | Magnoliopsida |
| Combretum aphanopetalum Engl. & Diels | Combretaceae | Magnoliopsida |
| Combretum carringtonianum Exell & J.G.García | Combretaceae | Magnoliopsida |
| Combretum clarense Jongkind | Combretaceae | Magnoliopsida |
| Combretum comosum G.Don | Combretaceae | Magnoliopsida |
| Combretum conchipetalum Engl. & Diels | Combretaceae | Magnoliopsida |
| Combretum cuspidatum Planch. ex Benth. | Combretaceae | Magnoliopsida |
| Combretum demeusei De Wild. | Combretaceae | Magnoliopsida |
| Combretum esteriense Jongkind | Combretaceae | Magnoliopsida |
| Combretum exellii Jongkind | Combretaceae | Magnoliopsida |
| Combretum hensii Engl. & Diels | Combretaceae | Magnoliopsida |
| Combretum indicum (L.) DeFilipps | Combretaceae | Magnoliopsida |
| Combretum inflatum Jongkind | Combretaceae | Magnoliopsida |
| Combretum latialatum Engl. | Combretaceae | Magnoliopsida |
| Combretum mannii Engl. & Diels | Combretaceae | Magnoliopsida |
| Combretum oudenhovenii Jongkind | Combretaceae | Magnoliopsida |
| Combretum oyemense Exell | Combretaceae | Magnoliopsida |
| Combretum paradoxum Welw. ex M.A.Lawson | Combretaceae | Magnoliopsida |
| Combretum pecoense Exell | Combretaceae | Magnoliopsida |
| Combretum platypterum (Welw.) Hutch. & Dalziel | Combretaceae | Magnoliopsida |
| Combretum psidioides Welw. | Combretaceae | Magnoliopsida |
| Combretum rabiense Jongkind | Combretaceae | Magnoliopsida |
| Combretum sericeum G.Don | Combretaceae | Magnoliopsida |
| Combretum sordidum Exell | Combretaceae | Magnoliopsida |
| Conocarpus erectus L. | Combretaceae | Magnoliopsida |
| Laguncularia racemosa (L.) C.F.Gaertn. | Combretaceae | Magnoliopsida |
| Terminalia catappa L. | Combretaceae | Magnoliopsida |
| Terminalia ivorensis A.Chev. | Combretaceae | Magnoliopsida |
| Terminalia laxiflora Engl. | Combretaceae | Magnoliopsida |
| Amischotolype tenuis (C.B.Clarke) R.S.Rao | Commelinaceae | Magnoliopsida |
| Aneilema beniniense (P.Beauv.) Kunth | Commelinaceae | Magnoliopsida |
| Aneilema dispermum Brenan | Commelinaceae | Magnoliopsida |
| Buforrestia mannii C.B.Clarke | Commelinaceae | Magnoliopsida |
| Buforrestia obovata Brenan | Commelinaceae | Magnoliopsida |
| Commelina cameroonensis J.K.Morton | Commelinaceae | Magnoliopsida |
| Commelina capitata Benth. | Commelinaceae | Magnoliopsida |
| Palisota ambigua (P.Beauv.) C.B.Clarke | Commelinaceae | Magnoliopsida |
| Palisota barteri Hook.f. | Commelinaceae | Magnoliopsida |
| Palisota bogneri Brenan | Commelinaceae | Magnoliopsida |
| Palisota brachythyrsa Mildbr. | Commelinaceae | Magnoliopsida |
| Palisota hirsuta (Thunb.) K.Schum. | Commelinaceae | Magnoliopsida |
| Palisota satabiei Brenan | Commelinaceae | Magnoliopsida |
| Pollia condensata C.B.Clarke | Commelinaceae | Magnoliopsida |
| Polyspatha paniculata Benth. | Commelinaceae | Magnoliopsida |
| Stanfieldiella imperforata (C.B.Clarke) Brenan | Commelinaceae | Magnoliopsida |
| Tricarpelema africanum Faden | Commelinaceae | Magnoliopsida |
| Aedesia glabra O.Hoffm. | Compositae | Magnoliopsida |
| Aspilia rudis Oliv. & Hiern | Compositae | Magnoliopsida |
| Bothriocline longipes (Oliv. & Hiern) N.E.Br. | Compositae | Magnoliopsida |
| Chromolaena odorata (L.) R.M.King & H.Rob. | Compositae | Magnoliopsida |
| Conyza pyrrhopappa Sch.Bip. ex A.Rich. | Compositae | Magnoliopsida |
| Crassocephalum montuosum (S.Moore) Milne-Redh. | Compositae | Magnoliopsida |
| Crassocephalum vitellinum (Benth.) S.Moore | Compositae | Magnoliopsida |
| Emilia lisowskiana C.Jeffrey | Compositae | Magnoliopsida |
| Emilia longiramea (S.Moore) C.Jeffrey | Compositae | Magnoliopsida |
| Emilia praetermissa Milne-Redh. | Compositae | Magnoliopsida |
| Erlangea plumosa Sch.Bip. | Compositae | Magnoliopsida |
| Helichrysum globosum Sch.Bip. | Compositae | Magnoliopsida |
| Helichrysum keilii Moeser | Compositae | Magnoliopsida |
| Lactuca inermis Forssk. | Compositae | Magnoliopsida |
| Microglossa densiflora Hook.f. | Compositae | Magnoliopsida |
| Nothovernonia purpurea (Sch.Bip. ex Walp.) H.Rob. & V.A.Funk | Compositae | Magnoliopsida |
| Stomatanthes africanus (Oliv. & Hiern) R.M.King & H.Rob. | Compositae | Magnoliopsida |
| Vernonia frondosa Oliv. & Hiern | Compositae | Magnoliopsida |
| Vernonia hochstetteri Sch.Bip. ex Walp. | Compositae | Magnoliopsida |
| Vernonia hymenolepis A.Rich. | Compositae | Magnoliopsida |
| Vernonia migeodii S.Moore | Compositae | Magnoliopsida |
| Vernonia nestor S.Moore | Compositae | Magnoliopsida |
| Vernonia potamophila Klatt | Compositae | Magnoliopsida |
| Vernonia procera O.Hoffm. | Compositae | Magnoliopsida |
| Agelaea gabonensis Jongkind | Connaraceae | Magnoliopsida |
| Agelaea palmata Jongkind | Connaraceae | Magnoliopsida |
| Agelaea paradoxa Gilg | Connaraceae | Magnoliopsida |
| Agelaea poggeana Gilg | Connaraceae | Magnoliopsida |
| Agelaea rubiginosa Gilg | Connaraceae | Magnoliopsida |
| Cnestis corniculata Lam. | Connaraceae | Magnoliopsida |
| Cnestis ferruginea Vahl ex DC. | Connaraceae | Magnoliopsida |
| Cnestis macrantha Baill. | Connaraceae | Magnoliopsida |
| Cnestis mannii (Baker) Schellenb. | Connaraceae | Magnoliopsida |
| Cnestis uncata Lemmens | Connaraceae | Magnoliopsida |
| Cnestis urens Gilg | Connaraceae | Magnoliopsida |
| Connarus africanus Lam. | Connaraceae | Magnoliopsida |
| Connarus griffonianus Baill. | Connaraceae | Magnoliopsida |
| Connarus longistipitatus Gilg | Connaraceae | Magnoliopsida |
| Hemandradenia mannii Stapf | Connaraceae | Magnoliopsida |
| Jollydora duparquetiana (Baill.) Pierre | Connaraceae | Magnoliopsida |
| Manotes expansa Sol. ex Planch. | Connaraceae | Magnoliopsida |
| Manotes macrantha (Gilg) Schellenb. | Connaraceae | Magnoliopsida |
| Rourea calophylla (Gilg ex Schellenb.) Jongkind | Connaraceae | Magnoliopsida |
| Rourea calophylloides (Schellenb.) Jongkind | Connaraceae | Magnoliopsida |
| Rourea myriantha Baill. | Connaraceae | Magnoliopsida |
| Rourea obliquifoliolata Gilg | Connaraceae | Magnoliopsida |
| Bonamia thunbergiana (Roem. & Schult.) F.N.Williams | Convolvulaceae | Magnoliopsida |
| Calycobolus acuminatus (Pilg.) Heine | Convolvulaceae | Magnoliopsida |
| Calycobolus africanus (G.Don) Heine | Convolvulaceae | Magnoliopsida |
| Calycobolus cabrae (De Wild. & T.Durand) Heine | Convolvulaceae | Magnoliopsida |
| Cuscuta campestris Yunck. | Convolvulaceae | Magnoliopsida |
| Dipteropeltis poranoides Hallier f. | Convolvulaceae | Magnoliopsida |
| Ipomoea asarifolia (Desr.) Roem. & Schult. | Convolvulaceae | Magnoliopsida |
| Ipomoea imperati (Vahl) Griseb. | Convolvulaceae | Magnoliopsida |
| Ipomoea pes-caprae (L.) R.Br. | Convolvulaceae | Magnoliopsida |
| Ipomoea triloba L. | Convolvulaceae | Magnoliopsida |
| Jacquemontia tamnifolia (L.) Griseb. | Convolvulaceae | Magnoliopsida |
| Merremia dissecta (Jacq.) Hallier f. | Convolvulaceae | Magnoliopsida |
| Neuropeltis acuminata (P.Beauv.) Benth. | Convolvulaceae | Magnoliopsida |
| Neuropeltis eladii Breteler | Convolvulaceae | Magnoliopsida |
| Cardamine trichocarpa Hochst. ex A.Rich. | Cruciferae | Magnoliopsida |
| Coccinia heterophylla (Hook.f.) Holstein | Cucurbitaceae | Magnoliopsida |
| Coccinia racemiflora Keraudren | Cucurbitaceae | Magnoliopsida |
| Cogniauxia podolaena Baill. | Cucurbitaceae | Magnoliopsida |
| Peponium vogelii (Hook.f.) Engl. | Cucurbitaceae | Magnoliopsida |
| Pilogyne keayana (R.Fern. & A.Fern.) W.J.de Wilde & Duyfjes | Cucurbitaceae | Magnoliopsida |
| Pilogyne minutiflora (Cogn.) W.J.de Wilde & Duyfjes | Cucurbitaceae | Magnoliopsida |
| Raphidiocystis mannii Hook.f. | Cucurbitaceae | Magnoliopsida |
| Actinoschoenus filiformis Benth. | Cyperaceae | Magnoliopsida |
| Afrotrilepis pilosa (Boeckeler) J.Raynal | Cyperaceae | Magnoliopsida |
| Bulbostylis barbata (Rottb.) C.B.Clarke | Cyperaceae | Magnoliopsida |
| Bulbostylis cardiocarpoides Cherm. | Cyperaceae | Magnoliopsida |
| Bulbostylis coleotricha (Hochst. ex A.Rich.) C.B.Clarke | Cyperaceae | Magnoliopsida |
| Bulbostylis laniceps C.B.Clarke ex T.Durand & Schinz | Cyperaceae | Magnoliopsida |
| Bulbostylis pilosa (Willd.) Cherm. | Cyperaceae | Magnoliopsida |
| Carex echinochloe Kunze | Cyperaceae | Magnoliopsida |
| Cyperus afrosylvestris Lye | Cyperaceae | Magnoliopsida |
| Cyperus crassipes Vahl | Cyperaceae | Magnoliopsida |
| Cyperus dilatatus Schumach. | Cyperaceae | Magnoliopsida |
| Cyperus fertilis Boeckeler | Cyperaceae | Magnoliopsida |
| Cyperus fibrillosus Kük. | Cyperaceae | Magnoliopsida |
| Cyperus iria L. | Cyperaceae | Magnoliopsida |
| Cyperus ligularis L. | Cyperaceae | Magnoliopsida |
| Cyperus peruvianus (Lam.) F.N.Williams | Cyperaceae | Magnoliopsida |
| Cyperus pustulatus Vahl | Cyperaceae | Magnoliopsida |
| Cyperus tenax Boeckeler | Cyperaceae | Magnoliopsida |
| Cyperus tenuis Sw. | Cyperaceae | Magnoliopsida |
| Eleocharis geniculata (L.) Roem. & Schult. | Cyperaceae | Magnoliopsida |
| Eleocharis mutata (L.) Roem. & Schult. | Cyperaceae | Magnoliopsida |
| Eleocharis naumanniana Boeckeler | Cyperaceae | Magnoliopsida |
| Fimbristylis bisumbellata (Forssk.) Bubani | Cyperaceae | Magnoliopsida |
| Fimbristylis cymosa R.Br. | Cyperaceae | Magnoliopsida |
| Fimbristylis miliacea (L.) Vahl | Cyperaceae | Magnoliopsida |
| Fimbristylis pilosa Vahl | Cyperaceae | Magnoliopsida |
| Lipocarpha filiformis (Vahl) Kunth | Cyperaceae | Magnoliopsida |
| Mapania africana Boeckeler | Cyperaceae | Magnoliopsida |
| Mapania amplivaginata K.Schum. | Cyperaceae | Magnoliopsida |
| Mapania heteromorpha (Nelmes) Lye | Cyperaceae | Magnoliopsida |
| Mapania heterophylla (Boeckeler) Lye | Cyperaceae | Magnoliopsida |
| Mapania mannii C.B.Clarke | Cyperaceae | Magnoliopsida |
| Mapania pubisquama Cherm. | Cyperaceae | Magnoliopsida |
| Mapania purpuriceps (C.B.Clarke) J.Raynal | Cyperaceae | Magnoliopsida |
| Mapania scaberrima (Boeckeler) C.B.Clarke | Cyperaceae | Magnoliopsida |
| Mapania sylvatica Aubl. | Cyperaceae | Magnoliopsida |
| Mapania testui Cherm. | Cyperaceae | Magnoliopsida |
| Nemum spadiceum (Lam.) Desv. ex Ham. | Cyperaceae | Magnoliopsida |
| Pycreus polystachyos (Rottb.) P.Beauv. | Cyperaceae | Magnoliopsida |
| Remirea maritima Aubl. | Cyperaceae | Magnoliopsida |
| Rhynchospora holoschoenoides (Rich.) Herter | Cyperaceae | Magnoliopsida |
| Rhynchospora rubra (Lour.) Makino | Cyperaceae | Magnoliopsida |
| Scleria boivinii Steud. | Cyperaceae | Magnoliopsida |
| Scleria naumanniana Boeckeler | Cyperaceae | Magnoliopsida |
| Scleria spiciformis Benth. | Cyperaceae | Magnoliopsida |
| Scleria vogelii C.B.Clarke | Cyperaceae | Magnoliopsida |
| Dichapetalum acuminatum De Wild. | Dichapetalaceae | Magnoliopsida |
| Dichapetalum affine (Planch. ex Benth.) Breteler | Dichapetalaceae | Magnoliopsida |
| Dichapetalum angolense Chodat | Dichapetalaceae | Magnoliopsida |
| Dichapetalum bangii (Didr.) Engl. | Dichapetalaceae | Magnoliopsida |
| Dichapetalum barbatum Breteler | Dichapetalaceae | Magnoliopsida |
| Dichapetalum beilschmiedioides Breteler | Dichapetalaceae | Magnoliopsida |
| Dichapetalum bellum Breteler | Dichapetalaceae | Magnoliopsida |
| Dichapetalum berendinae Breteler | Dichapetalaceae | Magnoliopsida |
| Dichapetalum bodyi De Wild. | Dichapetalaceae | Magnoliopsida |
| Dichapetalum chalotii Pellegr. | Dichapetalaceae | Magnoliopsida |
| Dichapetalum congoense Engl. & Ruhland | Dichapetalaceae | Magnoliopsida |
| Dichapetalum cymulosum Engl. | Dichapetalaceae | Magnoliopsida |
| Dichapetalum dewevrei De Wild. & T.Durand | Dichapetalaceae | Magnoliopsida |
| Dichapetalum gabonense Engl. | Dichapetalaceae | Magnoliopsida |
| Dichapetalum geminostellatum Breteler | Dichapetalaceae | Magnoliopsida |
| Dichapetalum glomeratum Engl. | Dichapetalaceae | Magnoliopsida |
| Dichapetalum heudelotii (Planch. ex Oliv.) Baill. | Dichapetalaceae | Magnoliopsida |
| Dichapetalum insigne Engl. | Dichapetalaceae | Magnoliopsida |
| Dichapetalum leucocarpum Breteler | Dichapetalaceae | Magnoliopsida |
| Dichapetalum librevillense Pellegr. | Dichapetalaceae | Magnoliopsida |
| Dichapetalum lujae De Wild. & T.Durand | Dichapetalaceae | Magnoliopsida |
| Dichapetalum madagascariense Poir. | Dichapetalaceae | Magnoliopsida |
| Dichapetalum mathisii Breteler | Dichapetalaceae | Magnoliopsida |
| Dichapetalum mombuttense Engl. | Dichapetalaceae | Magnoliopsida |
| Dichapetalum mundense Engl. | Dichapetalaceae | Magnoliopsida |
| Dichapetalum oblongum (Hook.f. ex Benth.) Engl. | Dichapetalaceae | Magnoliopsida |
| Dichapetalum parvifolium Engl. | Dichapetalaceae | Magnoliopsida |
| Dichapetalum pulchrum Breteler | Dichapetalaceae | Magnoliopsida |
| Dichapetalum rudatisii Engl. | Dichapetalaceae | Magnoliopsida |
| Dichapetalum staudtii Engl. | Dichapetalaceae | Magnoliopsida |
| Dichapetalum thollonii Pellegr. | Dichapetalaceae | Magnoliopsida |
| Dichapetalum zenkeri Engl. | Dichapetalaceae | Magnoliopsida |
| Tapura africana Oliv. | Dichapetalaceae | Magnoliopsida |
| Tapura arachnoidea Breteler | Dichapetalaceae | Magnoliopsida |
| Tapura bouquetiana N.Hallé & Heine | Dichapetalaceae | Magnoliopsida |
| Tapura carinata Breteler | Dichapetalaceae | Magnoliopsida |
| Tetracera alnifolia Willd. | Dilleniaceae | Magnoliopsida |
| Dioncophyllum thollonii Baill. | Dioncophyllaceae | Magnoliopsida |
| Dioscorea minutiflora Engl. | Dioscoreaceae | Magnoliopsida |
| Dioscorea semperflorens Uline | Dioscoreaceae | Magnoliopsida |
| Trillesanthus excelsus Pierre | Dipterocarpaceae | Magnoliopsida |
| Drosera indica L. | Droseraceae | Magnoliopsida |
| Diospyros bipindensis Gürke | Ebenaceae | Magnoliopsida |
| Diospyros canaliculata De Wild. | Ebenaceae | Magnoliopsida |
| Diospyros cinnabarina (Gürke) F.White | Ebenaceae | Magnoliopsida |
| Diospyros conocarpa Gürke & K.Schum. | Ebenaceae | Magnoliopsida |
| Diospyros crassiflora Hiern | Ebenaceae | Magnoliopsida |
| Diospyros dendo Welw. ex Hiern | Ebenaceae | Magnoliopsida |
| Diospyros fragrans Gürke | Ebenaceae | Magnoliopsida |
| Diospyros gabunensis Gürke | Ebenaceae | Magnoliopsida |
| Diospyros gilletii De Wild. | Ebenaceae | Magnoliopsida |
| Diospyros hoyleana F.White | Ebenaceae | Magnoliopsida |
| Diospyros iturensis (Gürke) Letouzey & F.White | Ebenaceae | Magnoliopsida |
| Diospyros mannii Hiern | Ebenaceae | Magnoliopsida |
| Diospyros monbuttensis Gürke | Ebenaceae | Magnoliopsida |
| Diospyros obliquifolia (Hiern ex Gürke) F.White | Ebenaceae | Magnoliopsida |
| Diospyros physocalycina Gürke | Ebenaceae | Magnoliopsida |
| Diospyros piscatoria Gürke | Ebenaceae | Magnoliopsida |
| Diospyros preussii Gürke | Ebenaceae | Magnoliopsida |
| Diospyros rabiensis Breteler | Ebenaceae | Magnoliopsida |
| Diospyros rubicunda Gürke | Ebenaceae | Magnoliopsida |
| Diospyros simulans F.White | Ebenaceae | Magnoliopsida |
| Diospyros spec.nov. nr. cinnabarina Dauby | Ebenaceae | Magnoliopsida |
| Diospyros suaveolens Gürke | Ebenaceae | Magnoliopsida |
| Diospyros zenkeri (Gürke) F.White | Ebenaceae | Magnoliopsida |
| Eriocaulon latifolium Sm. | Eriocaulaceae | Magnoliopsida |
| Mesanthemum prescottianum (Bong.) Körn. | Eriocaulaceae | Magnoliopsida |
| Mesanthemum radicans (Benth.) Körn. | Eriocaulaceae | Magnoliopsida |
| Aneulophus africanus Benth. | Erythroxylaceae | Magnoliopsida |
| Afrotrewia kamerunica Pax & K.Hoffm. | Euphorbiaceae | Magnoliopsida |
| Alchornea floribunda Müll.Arg. | Euphorbiaceae | Magnoliopsida |
| Alchornea glabrata (Müll.Arg.) Prain | Euphorbiaceae | Magnoliopsida |
| Alchornea sp.nov. near hirtella O.Lachenaud | Euphorbiaceae | Magnoliopsida |
| Amanoa bracteosa Planch. | Euphorbiaceae | Magnoliopsida |
| Anthostema aubryanum Baill. | Euphorbiaceae | Magnoliopsida |
| Antidesma vogelianum Müll.Arg. | Euphorbiaceae | Magnoliopsida |
| Argomuellera macrophylla Pax | Euphorbiaceae | Magnoliopsida |
| Aubletiana macrostachys (Breteler) J.Murillo | Euphorbiaceae | Magnoliopsida |
| Bridelia ferruginea Benth. | Euphorbiaceae | Magnoliopsida |
| Chaetocarpus africanus Pax | Euphorbiaceae | Magnoliopsida |
| Chaetocarpus gabonensis Breteler | Euphorbiaceae | Magnoliopsida |
| Cleistanthus caudatus Pax | Euphorbiaceae | Magnoliopsida |
| Cleistanthus gabonensis Hutch. | Euphorbiaceae | Magnoliopsida |
| Cleistanthus itsoghensis Pellegr. | Euphorbiaceae | Magnoliopsida |
| Cleistanthus ngounyensis Pellegr. | Euphorbiaceae | Magnoliopsida |
| Croton dybowskii Hutch. | Euphorbiaceae | Magnoliopsida |
| Croton macrostachyus Hochst. ex Delile | Euphorbiaceae | Magnoliopsida |
| Crotonogyne gabunensis Pax | Euphorbiaceae | Magnoliopsida |
| Crotonogyne parvifolia Prain | Euphorbiaceae | Magnoliopsida |
| Crotonogyne poggei Pax | Euphorbiaceae | Magnoliopsida |
| Crotonogyne strigosa Prain | Euphorbiaceae | Magnoliopsida |
| Crotonogyne zenkeri Pax | Euphorbiaceae | Magnoliopsida |
| Cyrtogonone argentea (Pax) Prain | Euphorbiaceae | Magnoliopsida |
| Dichostemma glaucescens Pierre | Euphorbiaceae | Magnoliopsida |
| Discoglypremna caloneura (Pax) Prain | Euphorbiaceae | Magnoliopsida |
| Erythrococca africana (Baill.) Prain | Euphorbiaceae | Magnoliopsida |
| Erythrococca anomala (Juss. ex Poir.) Prain | Euphorbiaceae | Magnoliopsida |
| Erythrococca welwitschiana (Müll.Arg.) Prain | Euphorbiaceae | Magnoliopsida |
| Euphorbia glaucophylla Poir. | Euphorbiaceae | Magnoliopsida |
| Euphorbia letestui J.Raynal | Euphorbiaceae | Magnoliopsida |
| Grossera macrantha Pax | Euphorbiaceae | Magnoliopsida |
| Grossera paniculata Pax | Euphorbiaceae | Magnoliopsida |
| Grossera vignei Hoyle | Euphorbiaceae | Magnoliopsida |
| Gymnanthes inopinata (Prain) Esser | Euphorbiaceae | Magnoliopsida |
| Keayodendron bridelioides Leandri | Euphorbiaceae | Magnoliopsida |
| Klaineanthus gabonii Pierre | Euphorbiaceae | Magnoliopsida |
| Macaranga assas Amougou | Euphorbiaceae | Magnoliopsida |
| Macaranga barteri Müll.Arg. | Euphorbiaceae | Magnoliopsida |
| Macaranga gabunica Prain | Euphorbiaceae | Magnoliopsida |
| Macaranga heudelotii Baill. | Euphorbiaceae | Magnoliopsida |
| Macaranga hurifolia Beille | Euphorbiaceae | Magnoliopsida |
| Macaranga klaineana Pierre | Euphorbiaceae | Magnoliopsida |
| Macaranga monandra Müll.Arg. | Euphorbiaceae | Magnoliopsida |
| Macaranga poggei Pax | Euphorbiaceae | Magnoliopsida |
| Macaranga saccifera Pax | Euphorbiaceae | Magnoliopsida |
| Maesobotrya barteri (Baill.) Hutch. | Euphorbiaceae | Magnoliopsida |
| Maesobotrya bertramiana Büttner | Euphorbiaceae | Magnoliopsida |
| Maesobotrya cordulata J.Léonard | Euphorbiaceae | Magnoliopsida |
| Maesobotrya griffoniana (Baill.) Pierre ex Hutch. | Euphorbiaceae | Magnoliopsida |
| Maesobotrya klaineana (Pierre) J.Léonard | Euphorbiaceae | Magnoliopsida |
| Maesobotrya longipes Hutch. | Euphorbiaceae | Magnoliopsida |
| Maesobotrya oligantha O.Lachenaud & Breteler | Euphorbiaceae | Magnoliopsida |
| Maesobotrya pauciflora Pax | Euphorbiaceae | Magnoliopsida |
| Manniophyton fulvum Müll.Arg. | Euphorbiaceae | Magnoliopsida |
| Maprounea membranacea Pax & K.Hoffm. | Euphorbiaceae | Magnoliopsida |
| Mareya brevipes Pax | Euphorbiaceae | Magnoliopsida |
| Martretia quadricornis Beille | Euphorbiaceae | Magnoliopsida |
| Necepsia afzelii Prain | Euphorbiaceae | Magnoliopsida |
| Neoboutonia mannii Benth. & Hook.f. | Euphorbiaceae | Magnoliopsida |
| Pentabrachion reticulatum Müll.Arg. | Euphorbiaceae | Magnoliopsida |
| Phyllanthus delpyanus Hutch. | Euphorbiaceae | Magnoliopsida |
| Phyllanthus gabonensis Jean F.Brunel | Euphorbiaceae | Magnoliopsida |
| Phyllanthus polyanthus Pax | Euphorbiaceae | Magnoliopsida |
| Plagiocladus diandrus (Pax) Jean F.Brunel | Euphorbiaceae | Magnoliopsida |
| Plesiatropha klaineana Pierre | Euphorbiaceae | Magnoliopsida |
| Plukenetia conophora Müll.Arg. | Euphorbiaceae | Magnoliopsida |
| Pogonophora letouzeyi Feuillet | Euphorbiaceae | Magnoliopsida |
| Protomegabaria macrophylla Hutch. | Euphorbiaceae | Magnoliopsida |
| Protomegabaria stapfiana (Beille) Hutch. | Euphorbiaceae | Magnoliopsida |
| Pseudagrostistachys africana (Müll.Arg.) Pax & K.Hoffm. | Euphorbiaceae | Magnoliopsida |
| Pycnocoma macrophylla Benth. | Euphorbiaceae | Magnoliopsida |
| Sclerocroton cornutus (Pax) Kruijt & Roebers | Euphorbiaceae | Magnoliopsida |
| Spondianthus preussii Engl. | Euphorbiaceae | Magnoliopsida |
| Tetrorchidium didymostemon (Baill.) Pax & K.Hoffm. | Euphorbiaceae | Magnoliopsida |
| Tetrorchidium gabonense Breteler | Euphorbiaceae | Magnoliopsida |
| Tetrorchidium oppositifolium (Pax) Pax & K.Hoffm. | Euphorbiaceae | Magnoliopsida |
| Thecacoris grandifolia (Pax & K.Hoffm.) Govaerts | Euphorbiaceae | Magnoliopsida |
| Thecacoris leptobotrya (Müll.Arg.) Brenan | Euphorbiaceae | Magnoliopsida |
| Thecacoris stenopetala (Müll.Arg.) Müll.Arg. | Euphorbiaceae | Magnoliopsida |
| Thecacoris viridis (Müll.Arg.) Leandri ex G.L.Webster | Euphorbiaceae | Magnoliopsida |
| Uapaca guineensis Müll.Arg. | Euphorbiaceae | Magnoliopsida |
| Uapaca mole Pax | Euphorbiaceae | Magnoliopsida |
| Uapaca nitida Müll.Arg. | Euphorbiaceae | Magnoliopsida |
| Uapaca pynaertii De Wild. | Euphorbiaceae | Magnoliopsida |
| Uapaca sansibarica Pax | Euphorbiaceae | Magnoliopsida |
| Uapaca staudtii Pax | Euphorbiaceae | Magnoliopsida |
| Uapaca togoensis Pax | Euphorbiaceae | Magnoliopsida |
| Uapaca vanhouttei De Wild. | Euphorbiaceae | Magnoliopsida |
| Casearia barteri Mast. | Flacourtiaceae | Magnoliopsida |
| Casearia prismatocarpa Mast. | Flacourtiaceae | Magnoliopsida |
| Dasylepis racemosa Oliv. | Flacourtiaceae | Magnoliopsida |
| Homalium africanum (Hook.f.) Benth. | Flacourtiaceae | Magnoliopsida |
| Homalium letestui Pellegr. | Flacourtiaceae | Magnoliopsida |
| Mocquerysia multiflora Hua | Flacourtiaceae | Magnoliopsida |
| Oncoba brachyanthera Oliv. | Flacourtiaceae | Magnoliopsida |
| Oncoba breteleri Hul | Flacourtiaceae | Magnoliopsida |
| Oncoba crepiniana De Wild. & T.Durand | Flacourtiaceae | Magnoliopsida |
| Oncoba dentata Oliv. | Flacourtiaceae | Magnoliopsida |
| Oncoba flagelliflora (Mildbr.) Hul | Flacourtiaceae | Magnoliopsida |
| Oncoba glauca (P.Beauv.) Planch. | Flacourtiaceae | Magnoliopsida |
| Oncoba mannii Oliv. | Flacourtiaceae | Magnoliopsida |
| Oncoba ngounyensis (Pellegr.) Hul | Flacourtiaceae | Magnoliopsida |
| Phyllobotryon spathulatum Müll.Arg. | Flacourtiaceae | Magnoliopsida |
| Poggea gossweileri Exell | Flacourtiaceae | Magnoliopsida |
| Scottellia klaineana Pierre | Flacourtiaceae | Magnoliopsida |
| Trichostephanus gabonensis Breteler | Flacourtiaceae | Magnoliopsida |
| Flagellaria guineensis Schumach. | Flagellariaceae | Magnoliopsida |
| Anthocleista laxiflora Baker | Gentianaceae | Magnoliopsida |
| Anthocleista microphylla Wernham | Gentianaceae | Magnoliopsida |
| Anthocleista nobilis G.Don | Gentianaceae | Magnoliopsida |
| Congolanthus longidens (N.E.Br.) A.Raynal | Gentianaceae | Magnoliopsida |
| Neurotheca corymbosa Hua | Gentianaceae | Magnoliopsida |
| Neurotheca loeselioides (Spruce ex Progel) Baill. | Gentianaceae | Magnoliopsida |
| Voyria primuloides Baker | Gentianaceae | Magnoliopsida |
| Acanthonema strigosum Hook.f. | Gesneriaceae | Magnoliopsida |
| Epithema tenue C.B.Clarke | Gesneriaceae | Magnoliopsida |
| Trachystigma mannii C.B.Clarke | Gesneriaceae | Magnoliopsida |
| Scaevola plumieri (L.) Vahl | Goodeniaceae | Magnoliopsida |
| Andropogon africanus Franch. | Gramineae | Magnoliopsida |
| Andropogon gayanus Kunth | Gramineae | Magnoliopsida |
| Andropogon pseudapricus Stapf | Gramineae | Magnoliopsida |
| Anthephora cristata (Döll) Hack. ex De Wild. & T.Durand | Gramineae | Magnoliopsida |
| Centotheca lappacea (L.) Desv. | Gramineae | Magnoliopsida |
| Digitaria abyssinica (Hochst. ex A.Rich.) Stapf | Gramineae | Magnoliopsida |
| Digitaria horizontalis Willd. | Gramineae | Magnoliopsida |
| Elymandra androphila (Stapf) Stapf | Gramineae | Magnoliopsida |
| Eragrostis tremula (Lam.) Hochst. ex Steud. | Gramineae | Magnoliopsida |
| Euclasta condylotricha (Steud.) Stapf | Gramineae | Magnoliopsida |
| Guaduella macrostachys (K.Schum.) Pilg. | Gramineae | Magnoliopsida |
| Guaduella marantifolia Franch. | Gramineae | Magnoliopsida |
| Hyparrhenia subplumosa Stapf | Gramineae | Magnoliopsida |
| Isachne buettneri Hack. | Gramineae | Magnoliopsida |
| Leptaspis zeylanica Nees ex Steud. | Gramineae | Magnoliopsida |
| Megastachya mucronata (Poir.) P.Beauv. | Gramineae | Magnoliopsida |
| Melinis nerviglumis (Franch.) Zizka | Gramineae | Magnoliopsida |
| Microcalamus barbinodis Franch. | Gramineae | Magnoliopsida |
| Microcalamus convallarioides Stapf | Gramineae | Magnoliopsida |
| Panicum calvum Stapf | Gramineae | Magnoliopsida |
| Panicum congoense Franch. | Gramineae | Magnoliopsida |
| Panicum littorale Sosef [1] | Gramineae | Magnoliopsida |
| Panicum nervatum (Franch.) Stapf | Gramineae | Magnoliopsida |
| Panicum phragmitoides Stapf | Gramineae | Magnoliopsida |
| Paspalum paniculatum L. | Gramineae | Magnoliopsida |
| Pennisetum trachyphyllum Pilg. | Gramineae | Magnoliopsida |
| Perotis indica (L.) Kuntze | Gramineae | Magnoliopsida |
| Puelia ciliata Franch. | Gramineae | Magnoliopsida |
| Puelia olyriformis (Franch.) Clayton | Gramineae | Magnoliopsida |
| Puelia schumanniana Pilg. | Gramineae | Magnoliopsida |
| Schizachyrium pulchellum (Benth.) Stapf | Gramineae | Magnoliopsida |
| Schizachyrium sanguineum (Retz.) Alston | Gramineae | Magnoliopsida |
| Schizachyrium thollonii (Franch.) Stapf | Gramineae | Magnoliopsida |
| Setaria restioidea (Franch.) Stapf | Gramineae | Magnoliopsida |
| Sporobolus virginicus (L.) Kunth | Gramineae | Magnoliopsida |
| Stenotaphrum secundatum (Walter) Kuntze | Gramineae | Magnoliopsida |
| Allanblackia gabonensis (Pellegr.) Bamps | Guttiferae | Magnoliopsida |
| Garcinia conrauana Engl. | Guttiferae | Magnoliopsida |
| Garcinia epunctata Stapf | Guttiferae | Magnoliopsida |
| Garcinia gabonensis Sosef & Dauby | Guttiferae | Magnoliopsida |
| Garcinia kola Heckel | Guttiferae | Magnoliopsida |
| Garcinia le-testui Pellegr. | Guttiferae | Magnoliopsida |
| Garcinia lucida Vesque | Guttiferae | Magnoliopsida |
| Garcinia mannii Oliv. | Guttiferae | Magnoliopsida |
| Garcinia obliqua Sosef & Dauby | Guttiferae | Magnoliopsida |
| Garcinia punctata Oliv. | Guttiferae | Magnoliopsida |
| Garcinia quadrifaria (Oliv.) Pierre | Guttiferae | Magnoliopsida |
| Garcinia smeathmannii (Planch. & Triana) Oliv. | Guttiferae | Magnoliopsida |
| Pentadesma butyracea Sabine | Guttiferae | Magnoliopsida |
| Pentadesma grandifolia Baker f. | Guttiferae | Magnoliopsida |
| Symphonia globulifera L.f. | Guttiferae | Magnoliopsida |
| Illigera pentaphylla Welw. | Hernandiaceae | Magnoliopsida |
| Hoplestigma klaineanum Pierre | Hoplestigmataceae | Magnoliopsida |
| Afrostyrax kamerunensis Perkins & Gilg | Huaceae | Magnoliopsida |
| Afrostyrax lepidophyllus Mildbr. | Huaceae | Magnoliopsida |
| Hua gabonii Pierre | Huaceae | Magnoliopsida |
| Sacoglottis gabonensis (Baill.) Urb. | Humiriaceae | Magnoliopsida |
| Calophyllum inophyllum L. | Hypericaceae | Magnoliopsida |
| Endodesmia calophylloides Benth. | Hypericaceae | Magnoliopsida |
| Harungana madagascariensis Lam. ex Poir. | Hypericaceae | Magnoliopsida |
| Mammea africana Sabine | Hypericaceae | Magnoliopsida |
| Psorospermum membranaceum C.H.Wright | Hypericaceae | Magnoliopsida |
| Psorospermum staudtii Engl. | Hypericaceae | Magnoliopsida |
| Psorospermum tenuifolium Hook.f. | Hypericaceae | Magnoliopsida |
| Vismia rubescens Oliv. | Hypericaceae | Magnoliopsida |
| Curculigo pilosa (Schumach. & Thonn.) Engl. | Hypoxidaceae | Magnoliopsida |
| Alsodeiopsis mannii Oliv. | Icacinaceae | Magnoliopsida |
| Alsodeiopsis rubra Engl. | Icacinaceae | Magnoliopsida |
| Alsodeiopsis weissenborniana J.Braun & K.Schum. | Icacinaceae | Magnoliopsida |
| Alsodeiopsis zenkeri Engl. | Icacinaceae | Magnoliopsida |
| Desmostachys tenuifolius Oliv. | Icacinaceae | Magnoliopsida |
| Desmostachys vogelii (Miers) Stapf | Icacinaceae | Magnoliopsida |
| Icacina mannii Oliv. | Icacinaceae | Magnoliopsida |
| Iodes africana Welw. ex Oliv. | Icacinaceae | Magnoliopsida |
| Lasianthera africana P.Beauv. | Icacinaceae | Magnoliopsida |
| Lavigeria macrocarpa (Oliv.) Pierre | Icacinaceae | Magnoliopsida |
| Pyrenacantha acuminata Engl. | Icacinaceae | Magnoliopsida |
| Pyrenacantha glabrescens (Engl.) Engl. | Icacinaceae | Magnoliopsida |
| Rhaphiostylis ferruginea Engl. | Icacinaceae | Magnoliopsida |
| Rhaphiostylis preussii Engl. | Icacinaceae | Magnoliopsida |
| Gladiolus unguiculatus Baker | Iridaceae | Magnoliopsida |
| Desbordesia insignis Pierre | Irvingiaceae | Magnoliopsida |
| Irvingia gabonensis (Aubry-Lecomte ex O'Rorke) Baill. | Irvingiaceae | Magnoliopsida |
| Irvingia smithii Hook.f. | Irvingiaceae | Magnoliopsida |
| Irvingia wombolu Vermoesen | Irvingiaceae | Magnoliopsida |
| Klainedoxa gabonensis Pierre | Irvingiaceae | Magnoliopsida |
| Klainedoxa trillesii Pierre ex Tiegh. | Irvingiaceae | Magnoliopsida |
| Basilicum polystachyon (L.) Moench | Labiatae | Magnoliopsida |
| Clerodendrum bipindense Gürke | Labiatae | Magnoliopsida |
| Clerodendrum buettneri Gürke | Labiatae | Magnoliopsida |
| Clerodendrum fuscum Gürke | Labiatae | Magnoliopsida |
| Clerodendrum silvanum Henriq. | Labiatae | Magnoliopsida |
| Clerodendrum umbellatum Poir. | Labiatae | Magnoliopsida |
| Clerodendrum volubile P.Beauv. | Labiatae | Magnoliopsida |
| Plectranthus epilithicus B.J.Pollard | Labiatae | Magnoliopsida |
| Plectranthus melleri Baker | Labiatae | Magnoliopsida |
| Plectranthus monostachyus (P.Beauv.) B.J.Pollard | Labiatae | Magnoliopsida |
| Premna lucens A.Chev. | Labiatae | Magnoliopsida |
| Premna serratifolia L. | Labiatae | Magnoliopsida |
| Pycnostachys eminii Gürke | Labiatae | Magnoliopsida |
| Rotheca violacea (Gürke) Verdc. | Labiatae | Magnoliopsida |
| Vitex gabunensis Gürke | Labiatae | Magnoliopsida |
| Vitex grandifolia Gürke | Labiatae | Magnoliopsida |
| Vitex micrantha Gürke | Labiatae | Magnoliopsida |
| Vitex milnei W.Piep. | Labiatae | Magnoliopsida |
| Vitex phaeotricha Mildbr. ex W.Piep. | Labiatae | Magnoliopsida |
| Vitex rivularis Gürke | Labiatae | Magnoliopsida |
| Beilschmiedia fulva Robyns & R.Wilczek | Lauraceae | Magnoliopsida |
| Beilschmiedia mannii (Meisn.) Robyns & R.Wilczek | Lauraceae | Magnoliopsida |
| Hypodaphnis zenkeri (Engl.) Stapf | Lauraceae | Magnoliopsida |
| Ocotea gabonensis Fouilloy | Lauraceae | Magnoliopsida |
| Crateranthus talbotii Baker f. | Lecythidaceae | Magnoliopsida |
| Napoleonaea angolensis Welw. | Lecythidaceae | Magnoliopsida |
| Napoleonaea cuneata Jongkind | Lecythidaceae | Magnoliopsida |
| Napoleonaea egertonii Baker f. | Lecythidaceae | Magnoliopsida |
| Napoleonaea gabonensis Liben | Lecythidaceae | Magnoliopsida |
| Napoleonaea talbotii Baker f. | Lecythidaceae | Magnoliopsida |
| Napoleonaea vogelii Hook. & Planch. | Lecythidaceae | Magnoliopsida |
| Petersianthus macrocarpus (P.Beauv.) Liben | Lecythidaceae | Magnoliopsida |
| Afzelia africana Sm. ex Pers. | Leguminosae-Caes. | Magnoliopsida |
| Afzelia bella Harms | Leguminosae-Caes. | Magnoliopsida |
| Afzelia bipindensis Harms | Leguminosae-Caes. | Magnoliopsida |
| Annea laxiflora (Benth.) Mackinder & Wieringa | Leguminosae-Caes. | Magnoliopsida |
| Anthonotha acuminata (De Wild.) J.Léonard | Leguminosae-Caes. | Magnoliopsida |
| Anthonotha crassifolia (Baill.) J.Léonard | Leguminosae-Caes. | Magnoliopsida |
| Anthonotha ferruginea (Harms) J.Léonard | Leguminosae-Caes. | Magnoliopsida |
| Anthonotha fragrans (Baker f.) Exell & Hillc. | Leguminosae-Caes. | Magnoliopsida |
| Anthonotha gilletii (De Wild.) J.Léonard | Leguminosae-Caes. | Magnoliopsida |
| Anthonotha lamprophylla (Harms) J.Léonard | Leguminosae-Caes. | Magnoliopsida |
| Anthonotha macrophylla P.Beauv. | Leguminosae-Caes. | Magnoliopsida |
| Anthonotha mouandzae Breteler | Leguminosae-Caes. | Magnoliopsida |
| Anthonotha pynaertii (De Wild.) Exell & Hillc. | Leguminosae-Caes. | Magnoliopsida |
| Anthonotha stipulacea (Benth.) J.Léonard | Leguminosae-Caes. | Magnoliopsida |
| Anthonotha trunciflora (Harms) J.Léonard | Leguminosae-Caes. | Magnoliopsida |
| Anthonotha xanderi Breteler | Leguminosae-Caes. | Magnoliopsida |
| Aphanocalyx djumaensis (De Wild.) J.Léonard | Leguminosae-Caes. | Magnoliopsida |
| Aphanocalyx heitzii (Pellegr.) Wieringa | Leguminosae-Caes. | Magnoliopsida |
| Aphanocalyx ledermannii (Harms) Wieringa | Leguminosae-Caes. | Magnoliopsida |
| Aphanocalyx microphyllus (Harms) Wieringa | Leguminosae-Caes. | Magnoliopsida |
| Augouardia le-testui Pellegr. | Leguminosae-Caes. | Magnoliopsida |
| Baikiaea insignis Benth. | Leguminosae-Caes. | Magnoliopsida |
| Baikiaea robynsii Ghesq. ex Laing | Leguminosae-Caes. | Magnoliopsida |
| Berlinia auriculata Benth. | Leguminosae-Caes. | Magnoliopsida |
| Berlinia bracteosa Benth. | Leguminosae-Caes. | Magnoliopsida |
| Berlinia confusa Hoyle | Leguminosae-Caes. | Magnoliopsida |
| Berlinia congolensis (Baker f.) Keay | Leguminosae-Caes. | Magnoliopsida |
| Berlinia immaculata Mackinder & Wieringa | Leguminosae-Caes. | Magnoliopsida |
| Berlinia rabiensis Mackinder | Leguminosae-Caes. | Magnoliopsida |
| Berlinia razzifera Mackinder & Wieringa | Leguminosae-Caes. | Magnoliopsida |
| Bikinia aciculifera Wieringa | Leguminosae-Caes. | Magnoliopsida |
| Bikinia breynei (Bamps) Wieringa | Leguminosae-Caes. | Magnoliopsida |
| Bikinia congensis Wieringa | Leguminosae-Caes. | Magnoliopsida |
| Bikinia durandii (F.Hallé & Normand) Wieringa | Leguminosae-Caes. | Magnoliopsida |
| Bikinia evrardii (Bamps) Wieringa | Leguminosae-Caes. | Magnoliopsida |
| Bikinia grisea Wieringa | Leguminosae-Caes. | Magnoliopsida |
| Bikinia le-testui (Pellegr.) Wieringa | Leguminosae-Caes. | Magnoliopsida |
| Bikinia media Wieringa | Leguminosae-Caes. | Magnoliopsida |
| Bikinia pellegrinii (A.Chev.) Wieringa | Leguminosae-Caes. | Magnoliopsida |
| Bikinia spec.nov. Crystal Mts | Leguminosae-Caes. | Magnoliopsida |
| Brachystegia cynometroides Harms | Leguminosae-Caes. | Magnoliopsida |
| Brachystegia laurentii (De Wild.) Louis ex Hoyle | Leguminosae-Caes. | Magnoliopsida |
| Brachystegia mildbraedii Harms | Leguminosae-Caes. | Magnoliopsida |
| Cassia fikifiki Aubrév. & Pellegr. | Leguminosae-Caes. | Magnoliopsida |
| Copaifera mildbraedii Harms | Leguminosae-Caes. | Magnoliopsida |
| Copaifera religiosa J.Léonard | Leguminosae-Caes. | Magnoliopsida |
| Crudia harmsiana De Wild. | Leguminosae-Caes. | Magnoliopsida |
| Crudia klainei Pierre ex De Wild. | Leguminosae-Caes. | Magnoliopsida |
| Crudia zenkeri Harms ex De Wild. | Leguminosae-Caes. | Magnoliopsida |
| Cryptosepalum staudtii Harms | Leguminosae-Caes. | Magnoliopsida |
| Cynometra lujae De Wild. | Leguminosae-Caes. | Magnoliopsida |
| Cynometra mannii Oliv. | Leguminosae-Caes. | Magnoliopsida |
| Cynometra schlechteri Harms | Leguminosae-Caes. | Magnoliopsida |
| Cynometra sessiliflora Harms | Leguminosae-Caes. | Magnoliopsida |
| Dialium angolense Welw. ex Oliv. | Leguminosae-Caes. | Magnoliopsida |
| Dialium bipindense Harms | Leguminosae-Caes. | Magnoliopsida |
| Dialium englerianum Henriq. | Leguminosae-Caes. | Magnoliopsida |
| Dialium guineense Willd. | Leguminosae-Caes. | Magnoliopsida |
| Dialium lopense Breteler | Leguminosae-Caes. | Magnoliopsida |
| Didelotia africana Baill. | Leguminosae-Caes. | Magnoliopsida |
| Didelotia brevipaniculata J.Léonard | Leguminosae-Caes. | Magnoliopsida |
| Didelotia letouzeyi Pellegr. | Leguminosae-Caes. | Magnoliopsida |
| Didelotia minutiflora (A.Chev.) J.Léonard | Leguminosae-Caes. | Magnoliopsida |
| Didelotia pauli-sitae Letouzey | Leguminosae-Caes. | Magnoliopsida |
| Distemonanthus benthamianus Baill. | Leguminosae-Caes. | Magnoliopsida |
| Duparquetia orchidacea Baill. | Leguminosae-Caes. | Magnoliopsida |
| Erythrophleum ivorense A.Chev. | Leguminosae-Caes. | Magnoliopsida |
| Eurypetalum tessmannii Harms | Leguminosae-Caes. | Magnoliopsida |
| Gabonius ngouniensis (Pellegr.) Wieringa & Mackinder | Leguminosae-Caes. | Magnoliopsida |
| Gigasiphon gossweileri (Baker f.) Torre & Hillc. | Leguminosae-Caes. | Magnoliopsida |
| Gilbertiodendron breteleri Burgt | Leguminosae-Caes. | Magnoliopsida |
| Gilbertiodendron demonstrans (Baill.) J.Léonard | Leguminosae-Caes. | Magnoliopsida |
| Gilbertiodendron dewevrei (De Wild.) J.Léonard | Leguminosae-Caes. | Magnoliopsida |
| Gilbertiodendron diphyllum (Harms) Estrella & Devesa | Leguminosae-Caes. | Magnoliopsida |
| Gilbertiodendron klainei (Pierre ex Pellegr.) J.Léonard | Leguminosae-Caes. | Magnoliopsida |
| Gilbertiodendron ngouniense (Pellegr.) J.Léonard | Leguminosae-Caes. | Magnoliopsida |
| Gilbertiodendron ogoouense (Pellegr.) J.Léonard | Leguminosae-Caes. | Magnoliopsida |
| Gilbertiodendron preussii (Harms) J.Léonard | Leguminosae-Caes. | Magnoliopsida |
| Gilbertiodendron stipulaceum (Benth.) J.Léonard | Leguminosae-Caes. | Magnoliopsida |
| Gilbertiodendron unijugum (Pellegr.) J.Léonard | Leguminosae-Caes. | Magnoliopsida |
| Gilletiodendron kisantuense (Vermoesen ex De Wild.) J.Léonard | Leguminosae-Caes. | Magnoliopsida |
| Gilletiodendron pierreanum (Harms) J.Léonard | Leguminosae-Caes. | Magnoliopsida |
| Griffonia physocarpa Baill. | Leguminosae-Caes. | Magnoliopsida |
| Guibourtia demeusei (Harms) J.Léonard | Leguminosae-Caes. | Magnoliopsida |
| Guibourtia ehie (A.Chev.) J.Léonard | Leguminosae-Caes. | Magnoliopsida |
| Guibourtia pellegriniana J.Léonard | Leguminosae-Caes. | Magnoliopsida |
| Hymenostegia bakeriana Hutch. & Dalziel | Leguminosae-Caes. | Magnoliopsida |
| Hymenostegia elegans Wieringa & Mackinder | Leguminosae-Caes. | Magnoliopsida |
| Hymenostegia floribunda (Benth.) Harms | Leguminosae-Caes. | Magnoliopsida |
| Hymenostegia klainei Pierre ex Pellegr. | Leguminosae-Caes. | Magnoliopsida |
| Hymenostegia neoaubrevillei J.Léonard | Leguminosae-Caes. | Magnoliopsida |
| Hymenostegia normandii Pellegr. | Leguminosae-Caes. | Magnoliopsida |
| Hymenostegia pellegrinii (A.Chev.) J.Léonard | Leguminosae-Caes. | Magnoliopsida |
| Hymenostegia talbotii Baker f. | Leguminosae-Caes. | Magnoliopsida |
| Hymenostegia viridiflora Mackinder & Wieringa | Leguminosae-Caes. | Magnoliopsida |
| Isomacrolobium hallei Aubrév. | Leguminosae-Caes. | Magnoliopsida |
| Isomacrolobium leptorrhachis (Harms) Aubrév. & Pellegr. | Leguminosae-Caes. | Magnoliopsida |
| Isomacrolobium triplisomere (Pellegr.) Breteler | Leguminosae-Caes. | Magnoliopsida |
| Julbernardia brieyi (De Wild.) Troupin | Leguminosae-Caes. | Magnoliopsida |
| Julbernardia hochreutineri Pellegr. | Leguminosae-Caes. | Magnoliopsida |
| Julbernardia pellegriniana Troupin | Leguminosae-Caes. | Magnoliopsida |
| Julbernardia seretii (De Wild.) Troupin | Leguminosae-Caes. | Magnoliopsida |
| Leonardoxa africana (Baill.) Aubrév. | Leguminosae-Caes. | Magnoliopsida |
| Librevillea klainei (Pierre ex Harms) Hoyle | Leguminosae-Caes. | Magnoliopsida |
| Loesenera gabonensis Pellegr. | Leguminosae-Caes. | Magnoliopsida |
| Loesenera walkeri (A.Chev.) J.Léonard | Leguminosae-Caes. | Magnoliopsida |
| Microberlinia brazzavillensis A.Chev. | Leguminosae-Caes. | Magnoliopsida |
| Neochevalierodendron stephanii (A.Chev.) J.Léonard | Leguminosae-Caes. | Magnoliopsida |
| Oddoniodendron gambanum Ngok & Breteler | Leguminosae-Caes. | Magnoliopsida |
| Oddoniodendron micranthum (Harms) Baker f. | Leguminosae-Caes. | Magnoliopsida |
| Plagiosiphon emarginatus (Hutch. & Dalziel) J.Léonard | Leguminosae-Caes. | Magnoliopsida |
| Plagiosiphon gabonensis (A.Chev.) J.Léonard | Leguminosae-Caes. | Magnoliopsida |
| Plagiosiphon longitubus (Harms) J.Léonard | Leguminosae-Caes. | Magnoliopsida |
| Plagiosiphon multijugus (Harms) J.Léonard | Leguminosae-Caes. | Magnoliopsida |
| Plagiosiphon spec.nov. (inselbergs) Wieringa | Leguminosae-Caes. | Magnoliopsida |
| Prioria balsamifera (Vermoesen) Breteler | Leguminosae-Caes. | Magnoliopsida |
| Prioria mannii (Baill.) Breteler | Leguminosae-Caes. | Magnoliopsida |
| Prioria oxyphylla (Harms) Breteler | Leguminosae-Caes. | Magnoliopsida |
| Scorodophloeus zenkeri Harms | Leguminosae-Caes. | Magnoliopsida |
| Sindora klaineana Pierre ex Pellegr. | Leguminosae-Caes. | Magnoliopsida |
| Sindoropsis le-testui (Pellegr.) J.Léonard | Leguminosae-Caes. | Magnoliopsida |
| Talbotiella bakossiensis Cheek ex Mackinder, Wieringa & Burgt | Leguminosae-Caes. | Magnoliopsida |
| Tamarindus indica L. | Leguminosae-Caes. | Magnoliopsida |
| Tessmannia africana Harms | Leguminosae-Caes. | Magnoliopsida |
| Tessmannia anomala (Micheli) Harms | Leguminosae-Caes. | Magnoliopsida |
| Tessmannia lescrauwaetii (De Wild.) Harms | Leguminosae-Caes. | Magnoliopsida |
| Tessmannia macrantha Breteler | Leguminosae-Caes. | Magnoliopsida |
| Tetraberlinia apiphila Wieringa | Leguminosae-Caes. | Magnoliopsida |
| Tetraberlinia bifoliolata (Harms) Hauman | Leguminosae-Caes. | Magnoliopsida |
| Tetraberlinia longiracemosa (A.Chev.) Wieringa | Leguminosae-Caes. | Magnoliopsida |
| Tetraberlinia moreliana Aubrév. | Leguminosae-Caes. | Magnoliopsida |
| Tetraberlinia polyphylla (Harms) J.Léonard ex Voorh. | Leguminosae-Caes. | Magnoliopsida |
| Zenkerella citrina Taub. | Leguminosae-Caes. | Magnoliopsida |
| Albizia laurentii De Wild. | Leguminosae-Mim. | Magnoliopsida |
| Calpocalyx dinklagei (Taub.) Harms | Leguminosae-Mim. | Magnoliopsida |
| Calpocalyx heitzii Pellegr. | Leguminosae-Mim. | Magnoliopsida |
| Calpocalyx klainei Pierre ex Harms | Leguminosae-Mim. | Magnoliopsida |
| Cathormion altissimum (Hook.f.) Hutch. & Dandy | Leguminosae-Mim. | Magnoliopsida |
| Entada africana Guill. & Perr. | Leguminosae-Mim. | Magnoliopsida |
| Entada gigas (L.) Fawc. & Rendle | Leguminosae-Mim. | Magnoliopsida |
| Fillaeopsis discophora Harms | Leguminosae-Mim. | Magnoliopsida |
| Neptunia oleracea Lour. | Leguminosae-Mim. | Magnoliopsida |
| Newtonia duncanthomasii Mackinder & Cheek | Leguminosae-Mim. | Magnoliopsida |
| Newtonia duparquetiana (Baill.) Keay | Leguminosae-Mim. | Magnoliopsida |
| Newtonia grandifolia Villiers | Leguminosae-Mim. | Magnoliopsida |
| Parkia bicolor A.Chev. | Leguminosae-Mim. | Magnoliopsida |
| Pentaclethra eetveldeana De Wild. & T.Durand | Leguminosae-Mim. | Magnoliopsida |
| Pentaclethra macrophylla Benth. | Leguminosae-Mim. | Magnoliopsida |
| Piptadeniastrum africanum (Hook.f.) Brenan | Leguminosae-Mim. | Magnoliopsida |
| Pseudoprosopis gilletii (De Wild.) Villiers | Leguminosae-Mim. | Magnoliopsida |
| Samanea dinklagei (Harms) Keay | Leguminosae-Mim. | Magnoliopsida |
| Tetrapleura tetraptera (Schumach. & Thonn.) Taub. | Leguminosae-Mim. | Magnoliopsida |
| Aganope lucida (Welw. ex Baker) Polhill | Leguminosae-Pap. | Magnoliopsida |
| Airyantha schweinfurthii (Taub.) Brummitt | Leguminosae-Pap. | Magnoliopsida |
| Alysicarpus ovalifolius (Schumach.) J.Léonard | Leguminosae-Pap. | Magnoliopsida |
| Angylocalyx oligophyllus (Baker) Baker f. | Leguminosae-Pap. | Magnoliopsida |
| Angylocalyx pynaertii De Wild. | Leguminosae-Pap. | Magnoliopsida |
| Baphia cuspidata Taub. | Leguminosae-Pap. | Magnoliopsida |
| Baphia laurentii De Wild. | Leguminosae-Pap. | Magnoliopsida |
| Baphia laurifolia Baill. | Leguminosae-Pap. | Magnoliopsida |
| Baphia leptostemma Baill. | Leguminosae-Pap. | Magnoliopsida |
| Baphia letestui Pellegr. | Leguminosae-Pap. | Magnoliopsida |
| Baphia megaphylla Breteler | Leguminosae-Pap. | Magnoliopsida |
| Baphia nitida Lodd. | Leguminosae-Pap. | Magnoliopsida |
| Baphia pilosa Baill. | Leguminosae-Pap. | Magnoliopsida |
| Baphia pubescens Hook.f. | Leguminosae-Pap. | Magnoliopsida |
| Camoensia brevicalyx Benth. | Leguminosae-Pap. | Magnoliopsida |
| Canavalia rosea (Sw.) DC. | Leguminosae-Pap. | Magnoliopsida |
| Craibia simplex Dunn | Leguminosae-Pap. | Magnoliopsida |
| Crotalaria leprieurii Guill. & Perr. | Leguminosae-Pap. | Magnoliopsida |
| Dalbergia bakeri Welw. ex Baker | Leguminosae-Pap. | Magnoliopsida |
| Dalbergia ecastaphyllum (L.) Taub. | Leguminosae-Pap. | Magnoliopsida |
| Dalbergia lactea Vatke | Leguminosae-Pap. | Magnoliopsida |
| Dalbergia louisii Cronquist | Leguminosae-Pap. | Magnoliopsida |
| Dalbergia oblongifolia G.Don | Leguminosae-Pap. | Magnoliopsida |
| Dalbergia saxatilis Hook.f. | Leguminosae-Pap. | Magnoliopsida |
| Dalhousiea africana S.Moore | Leguminosae-Pap. | Magnoliopsida |
| Desmodium gangeticum (L.) DC. | Leguminosae-Pap. | Magnoliopsida |
| Dewevrea bilabiata Micheli | Leguminosae-Pap. | Magnoliopsida |
| Dioclea hexandra (Ralph) Mabb. | Leguminosae-Pap. | Magnoliopsida |
| Eriosema batekense Maesen & G.M.Walters | Leguminosae-Pap. | Magnoliopsida |
| Eriosema griseum Baker | Leguminosae-Pap. | Magnoliopsida |
| Eriosema parviflorum E.Mey. | Leguminosae-Pap. | Magnoliopsida |
| Eriosema pellegrinii Tisser. | Leguminosae-Pap. | Magnoliopsida |
| Erythrina senegalensis DC. | Leguminosae-Pap. | Magnoliopsida |
| Haplormosia monophylla (Harms) Harms | Leguminosae-Pap. | Magnoliopsida |
| Indigofera atriceps Hook.f. | Leguminosae-Pap. | Magnoliopsida |
| Indigofera conjugata Baker | Leguminosae-Pap. | Magnoliopsida |
| Indigofera drepanocarpa Taub. | Leguminosae-Pap. | Magnoliopsida |
| Indigofera geminata Baker | Leguminosae-Pap. | Magnoliopsida |
| Indigofera mimosoides Baker | Leguminosae-Pap. | Magnoliopsida |
| Leptoderris laurentii De Wild. | Leguminosae-Pap. | Magnoliopsida |
| Leucomphalos brachycarpus (Harms) Breteler | Leguminosae-Pap. | Magnoliopsida |
| Lonchocarpus sericeus (Poir.) Kunth ex DC. | Leguminosae-Pap. | Magnoliopsida |
| Lotus discolor E.Mey. | Leguminosae-Pap. | Magnoliopsida |
| Machaerium lunatum (L.f.) Ducke | Leguminosae-Pap. | Magnoliopsida |
| Macrotyloma biflorum (Schumach. & Thonn.) Hepper | Leguminosae-Pap. | Magnoliopsida |
| Macrotyloma stenophyllum (Harms) Verdc. | Leguminosae-Pap. | Magnoliopsida |
| Millettia barteri (Benth.) Dunn | Leguminosae-Pap. | Magnoliopsida |
| Millettia bicolor Dunn | Leguminosae-Pap. | Magnoliopsida |
| Millettia cabrae De Wild. | Leguminosae-Pap. | Magnoliopsida |
| Millettia chrysophylla Dunn | Leguminosae-Pap. | Magnoliopsida |
| Millettia comosa (Micheli) Hauman | Leguminosae-Pap. | Magnoliopsida |
| Millettia lastoursvillensis Pellegr. | Leguminosae-Pap. | Magnoliopsida |
| Millettia laurentii De Wild. | Leguminosae-Pap. | Magnoliopsida |
| Millettia lecomtei Dunn | Leguminosae-Pap. | Magnoliopsida |
| Millettia macrophylla Benth. | Leguminosae-Pap. | Magnoliopsida |
| Millettia macroura Harms | Leguminosae-Pap. | Magnoliopsida |
| Millettia mannii Baker | Leguminosae-Pap. | Magnoliopsida |
| Millettia sanagana Harms | Leguminosae-Pap. | Magnoliopsida |
| Millettia sapinii De Wild. | Leguminosae-Pap. | Magnoliopsida |
| Millettia theuszii (Büttner) De Wild. | Leguminosae-Pap. | Magnoliopsida |
| Millettia thonningii (Schumach. & Thonn.) Baker | Leguminosae-Pap. | Magnoliopsida |
| Millettia versicolor Welw. ex Baker | Leguminosae-Pap. | Magnoliopsida |
| Millettia warneckei Harms | Leguminosae-Pap. | Magnoliopsida |
| Millettia wellensii De Wild. | Leguminosae-Pap. | Magnoliopsida |
| Neonotonia wightii (Graham ex Wight & Arn.) J.A.Lackey | Leguminosae-Pap. | Magnoliopsida |
| Ormocarpum verrucosum P.Beauv. | Leguminosae-Pap. | Magnoliopsida |
| Ostryocarpus riparius Hook.f. | Leguminosae-Pap. | Magnoliopsida |
| Pericopsis laxiflora (Benth. ex Baker) Meeuwen | Leguminosae-Pap. | Magnoliopsida |
| Platysepalum violaceum Welw. ex Baker | Leguminosae-Pap. | Magnoliopsida |
| Pterocarpus santalinoides L'Hér. ex DC. | Leguminosae-Pap. | Magnoliopsida |
| Pterocarpus soyauxii Taub. | Leguminosae-Pap. | Magnoliopsida |
| Pterocarpus tessmannii Harms | Leguminosae-Pap. | Magnoliopsida |
| Rhynchosia minima (L.) DC. | Leguminosae-Pap. | Magnoliopsida |
| Rhynchosia preussii (Harms) Taub. ex Harms | Leguminosae-Pap. | Magnoliopsida |
| Stylosanthes erecta P.Beauv. | Leguminosae-Pap. | Magnoliopsida |
| Tephrosia flexuosa G.Don | Leguminosae-Pap. | Magnoliopsida |
| Tephrosia lupinifolia DC. | Leguminosae-Pap. | Magnoliopsida |
| Uraria picta (Jacq.) DC. | Leguminosae-Pap. | Magnoliopsida |
| Vigna gracilis (Guill. & Perr.) Hook.f. | Leguminosae-Pap. | Magnoliopsida |
| Vigna pubigera Baker | Leguminosae-Pap. | Magnoliopsida |
| Zornia latifolia Sm. | Leguminosae-Pap. | Magnoliopsida |
| Genlisea africana Oliv. | Lentibulariaceae | Magnoliopsida |
| Utricularia spiralis Sm. | Lentibulariaceae | Magnoliopsida |
| Utricularia subulata L. | Lentibulariaceae | Magnoliopsida |
| Gloriosa carsonii Baker | Liliaceae | Magnoliopsida |
| Ctenolophon englerianus Mildbr. | Linaceae | Magnoliopsida |
| Hugonia gabunensis Engl. | Linaceae | Magnoliopsida |
| Hugonia micans Engl. | Linaceae | Magnoliopsida |
| Hugonia planchonii Hook.f. | Linaceae | Magnoliopsida |
| Hugonia platysepala Welw. ex Oliv. | Linaceae | Magnoliopsida |
| Phyllocosmus congolensis (De Wild. & T.Durand) T.Durand & H.Durand | Linaceae | Magnoliopsida |
| Phyllocosmus sessiliflorus Oliv. | Linaceae | Magnoliopsida |
| Pinacopodium congolense (S.Moore) Exell & Mendonça | Linaceae | Magnoliopsida |
| Mostuea brunonis Didr. | Loganiaceae | Magnoliopsida |
| Mostuea hirsuta (T.Anderson ex Benth. & Hook.f.) Baill. ex Baker | Loganiaceae | Magnoliopsida |
| Mostuea megaphylla R.D.Good | Loganiaceae | Magnoliopsida |
| Spigelia anthelmia L. | Loganiaceae | Magnoliopsida |
| Strychnos aculeata Soler. | Loganiaceae | Magnoliopsida |
| Strychnos boonei De Wild. | Loganiaceae | Magnoliopsida |
| Strychnos chrysophylla Gilg | Loganiaceae | Magnoliopsida |
| Strychnos cuminodora Leeuwenb. | Loganiaceae | Magnoliopsida |
| Strychnos dale De Wild. | Loganiaceae | Magnoliopsida |
| Strychnos elaeocarpa Gilg ex Leeuwenb. | Loganiaceae | Magnoliopsida |
| Strychnos icaja Baill. | Loganiaceae | Magnoliopsida |
| Strychnos longicaudata Gilg | Loganiaceae | Magnoliopsida |
| Strychnos mimfiensis Gilg ex Leeuwenb. | Loganiaceae | Magnoliopsida |
| Strychnos phaeotricha Gilg | Loganiaceae | Magnoliopsida |
| Strychnos splendens Gilg | Loganiaceae | Magnoliopsida |
| Strychnos staudtii Gilg | Loganiaceae | Magnoliopsida |
| Strychnos tricalysioides Hutch. & M.B.Moss | Loganiaceae | Magnoliopsida |
| Strychnos urceolata Leeuwenb. | Loganiaceae | Magnoliopsida |
| Strychnos variabilis De Wild. | Loganiaceae | Magnoliopsida |
| Englerina gabonensis (Engl.) Balle | Loranthaceae | Magnoliopsida |
| Globimetula cornutibracteata Balle ex Wiens & Polhill | Loranthaceae | Magnoliopsida |
| Helixanthera mannii (Oliv.) Danser | Loranthaceae | Magnoliopsida |
| Helixanthera subalata (De Wild.) Wiens & Polhill | Loranthaceae | Magnoliopsida |
| Phragmanthera polycrypta (Didr.) Balle | Loranthaceae | Magnoliopsida |
| Tapinanthus bangwensis (Engl. & K.Krause) Danser | Loranthaceae | Magnoliopsida |
| Acridocarpus longifolius (G.Don) Hook.f. | Malpighiaceae | Magnoliopsida |
| Acridocarpus macrocalyx Engl. | Malpighiaceae | Magnoliopsida |
| Acridocarpus smeathmannii (DC.) Guill. & Perr. | Malpighiaceae | Magnoliopsida |
| Heteropterys leona (Cav.) Exell | Malpighiaceae | Magnoliopsida |
| Hibiscus ludwigii Eckl. & Zeyh. | Malvaceae | Magnoliopsida |
| Hibiscus noldeae Baker f. | Malvaceae | Magnoliopsida |
| Hibiscus squamosus Hochr. | Malvaceae | Magnoliopsida |
| Hibiscus tiliaceus L. | Malvaceae | Magnoliopsida |
| Sida collina Schltdl. | Malvaceae | Magnoliopsida |
| Ataenidia conferta (Benth.) Milne-Redh. | Marantaceae | Magnoliopsida |
| Halopegia azurea (K.Schum.) K.Schum. | Marantaceae | Magnoliopsida |
| Haumania danckelmaniana (J.Braun & K.Schum.) Milne-Redh. | Marantaceae | Magnoliopsida |
| Haumania liebrechtsiana (De Wild. & T.Durand) J.Léonard | Marantaceae | Magnoliopsida |
| Hypselodelphys poggeana (K.Schum.) Milne-Redh. | Marantaceae | Magnoliopsida |
| Hypselodelphys violacea (Ridl.) Milne-Redh. | Marantaceae | Magnoliopsida |
| Marantochloa alba A.C.Ley | Marantaceae | Magnoliopsida |
| Marantochloa congensis (K.Schum.) J.Léonard & Mullend. | Marantaceae | Magnoliopsida |
| Marantochloa filipes (Benth.) Hutch. | Marantaceae | Magnoliopsida |
| Marantochloa leucantha (K.Schum.) Milne-Redh. | Marantaceae | Magnoliopsida |
| Megaphrynium gabonense Koechlin | Marantaceae | Magnoliopsida |
| Megaphrynium macrostachyum (Benth.) Milne-Redh. | Marantaceae | Magnoliopsida |
| Sarcophrynium brachystachyum (Benth.) K.Schum. | Marantaceae | Magnoliopsida |
| Sarcophrynium prionogonium (K.Schum.) K.Schum. | Marantaceae | Magnoliopsida |
| Sarcophrynium schweinfurthianum (Kuntze) Milne-Redh. | Marantaceae | Magnoliopsida |
| Trachyphrynium braunianum (K.Schum.) Baker | Marantaceae | Magnoliopsida |
| Medusandra mpomiana Letouzey & Satabié | Medusandraceae | Magnoliopsida |
| Soyauxia gabonensis Oliv. | Medusandraceae | Magnoliopsida |
| Soyauxia glabrescens Engl. | Medusandraceae | Magnoliopsida |
| Amphiblemma hallei Jacq.-Fél. | Melastomataceae | Magnoliopsida |
| Amphiblemma molle Hook.f. | Melastomataceae | Magnoliopsida |
| Amphiblemma setosum Hook.f. | Melastomataceae | Magnoliopsida |
| Amphiblemma soyauxii Cogn. | Melastomataceae | Magnoliopsida |
| Antherotoma naudinii Hook.f. | Melastomataceae | Magnoliopsida |
| Calvoa hirsuta Hook.f. | Melastomataceae | Magnoliopsida |
| Calvoa monticola A.Chev. ex Hutch. & Dalziel | Melastomataceae | Magnoliopsida |
| Calvoa orientalis Taub. | Melastomataceae | Magnoliopsida |
| Calvoa pulcherrima Gilg ex Engl. | Melastomataceae | Magnoliopsida |
| Calvoa seretii De Wild. | Melastomataceae | Magnoliopsida |
| Dicellandra barteri Hook.f. | Melastomataceae | Magnoliopsida |
| Dicellandra descoingsii Jacq.-Fél. | Melastomataceae | Magnoliopsida |
| Dicellandra glanduligera (Pellegr.) Jacq.-Fél. | Melastomataceae | Magnoliopsida |
| Dichaetanthera strigosa (Cogn.) Jacq.-Fél. | Melastomataceae | Magnoliopsida |
| Dinophora spenneroides Benth. | Melastomataceae | Magnoliopsida |
| Dissotis barteri Hook.f. | Melastomataceae | Magnoliopsida |
| Dissotis brazzae Cogn. | Melastomataceae | Magnoliopsida |
| Dissotis congolensis (Cogn. ex Büttner) Jacq.-Fél. | Melastomataceae | Magnoliopsida |
| Dissotis thollonii Cogn. ex Büttner | Melastomataceae | Magnoliopsida |
| Heterotis decumbens (P.Beauv.) Jacq.-Fél. | Melastomataceae | Magnoliopsida |
| Heterotis rupicola (Gilg ex Engl.) Jacq.-Fél. | Melastomataceae | Magnoliopsida |
| Medinilla mirabilis (Gilg) Jacq.-Fél. | Melastomataceae | Magnoliopsida |
| Melastomastrum segregatum (Benth.) A.Fern. & R.Fern. | Melastomataceae | Magnoliopsida |
| Memecylon aequidianum Jacq.-Fél. | Melastomataceae | Magnoliopsida |
| Memecylon afzelii G.Don | Melastomataceae | Magnoliopsida |
| Memecylon arcuato-marginatum Gilg ex Engl. | Melastomataceae | Magnoliopsida |
| Memecylon candidum Gilg | Melastomataceae | Magnoliopsida |
| Memecylon myrianthum Gilg | Melastomataceae | Magnoliopsida |
| Memecylon virescens Hook.f. | Melastomataceae | Magnoliopsida |
| Memecylon viride Hutch. & Dalziel | Melastomataceae | Magnoliopsida |
| Memecylon zenkeri Gilg | Melastomataceae | Magnoliopsida |
| Ochthocharis dicellandroides (Gilg) C.Hansen & Wickens | Melastomataceae | Magnoliopsida |
| Osbeckia tubulosa Sm. | Melastomataceae | Magnoliopsida |
| Spathandra blakeoides (G.Don) Jacq.-Fél. | Melastomataceae | Magnoliopsida |
| Tristemma hirtum P.Beauv. | Melastomataceae | Magnoliopsida |
| Tristemma littorale Benth. | Melastomataceae | Magnoliopsida |
| Warneckea cauliflora Jacq.-Fél. | Melastomataceae | Magnoliopsida |
| Warneckea floribunda Jacq.-Fél. | Melastomataceae | Magnoliopsida |
| Warneckea fosteri (Hutch. & Dalziel) Jacq.-Fél. | Melastomataceae | Magnoliopsida |
| Warneckea macrantha Jacq.-Fél. | Melastomataceae | Magnoliopsida |
| Warneckea membranifolia (Hook.f.) Jacq.-Fél. | Melastomataceae | Magnoliopsida |
| Warneckea pulcherrima (Gilg) Jacq.-Fél. | Melastomataceae | Magnoliopsida |
| Carapa dinklagei Harms | Meliaceae | Magnoliopsida |
| Carapa parviflora Harms | Meliaceae | Magnoliopsida |
| Ekebergia capensis Sparrm. | Meliaceae | Magnoliopsida |
| Entandrophragma candollei Harms | Meliaceae | Magnoliopsida |
| Heckeldora ledermannii (Harms) J.J.de Wilde | Meliaceae | Magnoliopsida |
| Heckeldora staudtii (Harms) Staner | Meliaceae | Magnoliopsida |
| Heckeldora trifoliolata J.J.de Wilde | Meliaceae | Magnoliopsida |
| Heckeldora zenkeri (Harms) Staner | Meliaceae | Magnoliopsida |
| Leplaea cauliflora E.J.M.Koenen & J.J.de Wilde | Meliaceae | Magnoliopsida |
| Leplaea cedrata (A.Chev.) E.J.M.Koenen & J.J.de Wilde | Meliaceae | Magnoliopsida |
| Leplaea laurentii (De Wild.) E.J.M.Koenen & J.J.de Wilde | Meliaceae | Magnoliopsida |
| Leplaea mayombensis (Pellegr.) Staner | Meliaceae | Magnoliopsida |
| Leplaea thompsonii (Sprague & Hutch.) E.J.M.Koenen & J.J.de Wilde | Meliaceae | Magnoliopsida |
| Lovoa trichilioides Harms | Meliaceae | Magnoliopsida |
| Neoguarea glomerulata (Harms) E.J.M.Koenen & J.J.de Wilde | Meliaceae | Magnoliopsida |
| Pterorhachis le-testui Pellegr. | Meliaceae | Magnoliopsida |
| Pterorhachis zenkeri Harms | Meliaceae | Magnoliopsida |
| Trichilia gilletii De Wild. | Meliaceae | Magnoliopsida |
| Trichilia monadelpha (Thonn.) J.J.de Wilde | Meliaceae | Magnoliopsida |
| Trichilia rubescens Oliv. | Meliaceae | Magnoliopsida |
| Trichilia welwitschii C.DC. | Meliaceae | Magnoliopsida |
| Turraea cabrae De Wild. & T.Durand | Meliaceae | Magnoliopsida |
| Turraea heterophylla Sm. | Meliaceae | Magnoliopsida |
| Turraea vogelii Hook.f. ex Benth. | Meliaceae | Magnoliopsida |
| Turraeanthus longipes Baill. | Meliaceae | Magnoliopsida |
| Kolobopetalum auriculatum Engl. | Menispermaceae | Magnoliopsida |
| Kolobopetalum ovatum Stapf | Menispermaceae | Magnoliopsida |
| Penianthus camerounensis A.Dekker | Menispermaceae | Magnoliopsida |
| Penianthus longifolius Miers | Menispermaceae | Magnoliopsida |
| Rhigiocarya racemifera Miers | Menispermaceae | Magnoliopsida |
| Synclisia scabrida Miers | Menispermaceae | Magnoliopsida |
| Triclisia patens Oliv. | Menispermaceae | Magnoliopsida |
| Glossocalyx brevipes Benth. | Monimiaceae | Magnoliopsida |
| Dorstenia africana (Baill.) C.C.Berg | Moraceae | Magnoliopsida |
| Dorstenia barteri Bureau | Moraceae | Magnoliopsida |
| Dorstenia ciliata Engl. | Moraceae | Magnoliopsida |
| Dorstenia cuspidata Hochst. ex A.Rich. | Moraceae | Magnoliopsida |
| Dorstenia elliptica Bureau | Moraceae | Magnoliopsida |
| Dorstenia kameruniana Engl. | Moraceae | Magnoliopsida |
| Dorstenia lujae De Wild. | Moraceae | Magnoliopsida |
| Dorstenia mannii Hook.f. | Moraceae | Magnoliopsida |
| Dorstenia oligogyna (Pellegr.) C.C.Berg | Moraceae | Magnoliopsida |
| Dorstenia picta Bureau | Moraceae | Magnoliopsida |
| Dorstenia poinsettiifolia Engl. | Moraceae | Magnoliopsida |
| Dorstenia turbinata Engl. | Moraceae | Magnoliopsida |
| Dorstenia yambuyaensis De Wild. | Moraceae | Magnoliopsida |
| Ficus ardisioides Warb. | Moraceae | Magnoliopsida |
| Ficus burretiana Mildbr. & Hutch. | Moraceae | Magnoliopsida |
| Ficus trichopoda Baker | Moraceae | Magnoliopsida |
| Ficus vogeliana (Miq.) Miq. | Moraceae | Magnoliopsida |
| Musanga cecropioides R.Br. ex Tedlie | Moraceae | Magnoliopsida |
| Myrianthus cuneifolius (Engl.) Engl. | Moraceae | Magnoliopsida |
| Myrianthus preussii Engl. | Moraceae | Magnoliopsida |
| Myrianthus serratus (Trécul) Benth. & Hook.f. | Moraceae | Magnoliopsida |
| Scyphosyce manniana Baill. | Moraceae | Magnoliopsida |
| Streblus usambarensis (Engl.) C.C.Berg | Moraceae | Magnoliopsida |
| Treculia acuminata Baill. | Moraceae | Magnoliopsida |
| Treculia africana Decne. | Moraceae | Magnoliopsida |
| Treculia obovoidea N.E.Br. | Moraceae | Magnoliopsida |
| Utsetela gabonensis Pellegr. | Moraceae | Magnoliopsida |
| Utsetela neglecta Jongkind | Moraceae | Magnoliopsida |
| Coelocaryon preussii Warb. | Myristicaceae | Magnoliopsida |
| Pycnanthus angolensis (Welw.) Warb. | Myristicaceae | Magnoliopsida |
| Scyphocephalium mannii (Benth.) Warb. | Myristicaceae | Magnoliopsida |
| Staudtia kamerunensis Warb. | Myristicaceae | Magnoliopsida |
| Ardisia buesgenii (Gilg & Schellenb.) Taton | Myrsinaceae | Magnoliopsida |
| Ardisia sadebeckiana Gilg | Myrsinaceae | Magnoliopsida |
| Ardisia staudtii Gilg | Myrsinaceae | Magnoliopsida |
| Ardisia zenkeri Gilg | Myrsinaceae | Magnoliopsida |
| Embelia schimperi Vatke | Myrsinaceae | Magnoliopsida |
| Maesa lanceolata Forssk. | Myrsinaceae | Magnoliopsida |
| Eugenia fernandopoana Engl. & Brehmer | Myrtaceae | Magnoliopsida |
| Eugenia klaineana (Pierre) Engl. | Myrtaceae | Magnoliopsida |
| Eugenia ogoouensis Amshoff | Myrtaceae | Magnoliopsida |
| Syzygium staudtii (Engl.) Mildbr. | Myrtaceae | Magnoliopsida |
| Campylospermum auriculatum Biss. | Ochnaceae | Magnoliopsida |
| Campylospermum bukobense (Gilg) Farron | Ochnaceae | Magnoliopsida |
| Campylospermum cabrae (Gilg) Farron | Ochnaceae | Magnoliopsida |
| Campylospermum calanthum (Gilg) Farron | Ochnaceae | Magnoliopsida |
| Campylospermum densiflorum (De Wild. & T.Durand) Farron | Ochnaceae | Magnoliopsida |
| Campylospermum descoingsii Farron | Ochnaceae | Magnoliopsida |
| Campylospermum duparquetianum (Baill.) Tiegh. | Ochnaceae | Magnoliopsida |
| Campylospermum dybowskii Tiegh. | Ochnaceae | Magnoliopsida |
| Campylospermum elongatum (Oliv.) Tiegh. | Ochnaceae | Magnoliopsida |
| Campylospermum excavatum (Tiegh.) Farron | Ochnaceae | Magnoliopsida |
| Campylospermum flavum (Schumach. & Thonn. ex Stapf) Farron | Ochnaceae | Magnoliopsida |
| Campylospermum gabonensis Biss. | Ochnaceae | Magnoliopsida |
| Campylospermum glaberrimum (P.Beauv.) Farron | Ochnaceae | Magnoliopsida |
| Campylospermum glaucifolium Biss. | Ochnaceae | Magnoliopsida |
| Campylospermum glaucum (Tiegh.) Farron | Ochnaceae | Magnoliopsida |
| Campylospermum laeve (De Wild. & T.Durand) Farron | Ochnaceae | Magnoliopsida |
| Campylospermum laxiflorum (De Wild. & T.Durand) Tiegh. | Ochnaceae | Magnoliopsida |
| Campylospermum lecomtei (Tiegh.) Farron | Ochnaceae | Magnoliopsida |
| Campylospermum longestipulatum (De Wild.) Biss. | Ochnaceae | Magnoliopsida |
| Campylospermum louisii Biss. & Sosef | Ochnaceae | Magnoliopsida |
| Campylospermum occidentalis Biss. | Ochnaceae | Magnoliopsida |
| Campylospermum oliveri (Tiegh.) Farron | Ochnaceae | Magnoliopsida |
| Campylospermum paucinervatum Sosef | Ochnaceae | Magnoliopsida |
| Campylospermum plicatum (Tiegh.) Biss. | Ochnaceae | Magnoliopsida |
| Campylospermum reticulatum (P.Beauv.) Farron | Ochnaceae | Magnoliopsida |
| Campylospermum sulcatum (Tiegh.) Farron | Ochnaceae | Magnoliopsida |
| Campylospermum umbricola (Tiegh.) Farron | Ochnaceae | Magnoliopsida |
| Campylospermum vogelii (Hook.f.) Farron | Ochnaceae | Magnoliopsida |
| Idertia axillaris (Oliv.) Farron | Ochnaceae | Magnoliopsida |
| Lophira alata Banks ex C.F.Gaertn. | Ochnaceae | Magnoliopsida |
| Ochna latisepala (Tiegh.) Bamps | Ochnaceae | Magnoliopsida |
| Ochna multiflora DC. | Ochnaceae | Magnoliopsida |
| Rhabdophyllum affine (Hook.f.) Tiegh. | Ochnaceae | Magnoliopsida |
| Rhabdophyllum arnoldianum (De Wild. & T.Durand) Tiegh. | Ochnaceae | Magnoliopsida |
| Rhabdophyllum calophyllum (Hook.f.) Tiegh. | Ochnaceae | Magnoliopsida |
| Rhabdophyllum letestui Farron | Ochnaceae | Magnoliopsida |
| Sauvagesia erecta L. | Ochnaceae | Magnoliopsida |
| Aptandra zenkeri Engl. | Olacaceae | Magnoliopsida |
| Coula edulis Baill. | Olacaceae | Magnoliopsida |
| Diogoa zenkeri (Engl.) Exell & Mendonça | Olacaceae | Magnoliopsida |
| Engomegoma gordonii Breteler | Olacaceae | Magnoliopsida |
| Heisteria trillesiana Pierre | Olacaceae | Magnoliopsida |
| Octoknema affinis Pierre | Olacaceae | Magnoliopsida |
| Olax gambecola Baill. | Olacaceae | Magnoliopsida |
| Olax latifolia Engl. | Olacaceae | Magnoliopsida |
| Olax mannii Oliv. | Olacaceae | Magnoliopsida |
| Olax staudtii Engl. | Olacaceae | Magnoliopsida |
| Ongokea gore (Hua) Pierre | Olacaceae | Magnoliopsida |
| Ptychopetalum petiolatum Oliv. | Olacaceae | Magnoliopsida |
| Strombosia grandifolia Hook.f. | Olacaceae | Magnoliopsida |
| Strombosia pustulata Oliv. | Olacaceae | Magnoliopsida |
| Strombosiopsis sereinii Breteler | Olacaceae | Magnoliopsida |
| Chionanthus mannii (Soler.) Stearn | Oleaceae | Magnoliopsida |
| Jasminum bakeri Scott-Elliot | Oleaceae | Magnoliopsida |
| Jasminum preussii Engl. & Knobl. | Oleaceae | Magnoliopsida |
| Aerangis arachnopus (Rchb.f.) Schltr. | Orchidaceae | Magnoliopsida |
| Ancistrorhynchus capitatus (Lindl.) Summerh. | Orchidaceae | Magnoliopsida |
| Ancistrorhynchus crystalensis P.J.Cribb & Laan | Orchidaceae | Magnoliopsida |
| Ancistrorhynchus recurvus Finet | Orchidaceae | Magnoliopsida |
| Ancistrorhynchus schumannii (Kraenzl.) Summerh. | Orchidaceae | Magnoliopsida |
| Ancistrorhynchus tenuicaulis Summerh. | Orchidaceae | Magnoliopsida |
| Angraecum affine Schltr. | Orchidaceae | Magnoliopsida |
| Angraecum bancoense Burg | Orchidaceae | Magnoliopsida |
| Angraecum eichlerianum Kraenzl. | Orchidaceae | Magnoliopsida |
| Angraecum gabonense Summerh. | Orchidaceae | Magnoliopsida |
| Bulbophyllum acutibracteatum De Wild. | Orchidaceae | Magnoliopsida |
| Bulbophyllum barbigerum Lindl. | Orchidaceae | Magnoliopsida |
| Bulbophyllum capituliflorum Rolfe | Orchidaceae | Magnoliopsida |
| Bulbophyllum cochleatum Lindl. | Orchidaceae | Magnoliopsida |
| Bulbophyllum colubrinum (Rchb.f.) Rchb.f. | Orchidaceae | Magnoliopsida |
| Bulbophyllum comatum Lindl. | Orchidaceae | Magnoliopsida |
| Bulbophyllum falcatum (Lindl.) Rchb.f. | Orchidaceae | Magnoliopsida |
| Bulbophyllum fuscum Lindl. | Orchidaceae | Magnoliopsida |
| Bulbophyllum imbricatum Lindl. | Orchidaceae | Magnoliopsida |
| Bulbophyllum intertextum Lindl. | Orchidaceae | Magnoliopsida |
| Bulbophyllum magnibracteatum Summerh. | Orchidaceae | Magnoliopsida |
| Bulbophyllum nigritianum Rendle | Orchidaceae | Magnoliopsida |
| Bulbophyllum oreonastes Rchb.f. | Orchidaceae | Magnoliopsida |
| Bulbophyllum pumilum (Sw.) Lindl. | Orchidaceae | Magnoliopsida |
| Bulbophyllum resupinatum Ridl. | Orchidaceae | Magnoliopsida |
| Bulbophyllum saltatorium Lindl. | Orchidaceae | Magnoliopsida |
| Calanthe sylvatica (Thouars) Lindl. | Orchidaceae | Magnoliopsida |
| Calyptrochilum emarginatum (Afzel. ex Sw.) Schltr. | Orchidaceae | Magnoliopsida |
| Chamaeangis ichneumonea (Lindl.) Schltr. | Orchidaceae | Magnoliopsida |
| Chamaeangis lecomtei (Finet) Schltr. | Orchidaceae | Magnoliopsida |
| Chamaeangis vesicata (Lindl.) Schltr. | Orchidaceae | Magnoliopsida |
| Chauliodon deflexicalcaratum (De Wild.) L.Jonss. | Orchidaceae | Magnoliopsida |
| Cribbia brachyceras (Summerh.) Senghas | Orchidaceae | Magnoliopsida |
| Cynorkis gabonensis Summerh. | Orchidaceae | Magnoliopsida |
| Cyrtorchis injoloensis (De Wild.) Schltr. | Orchidaceae | Magnoliopsida |
| Cyrtorchis ringens (Rchb.f.) Summerh. | Orchidaceae | Magnoliopsida |
| Diaphananthe bidens (Afzel. ex Sw.) Schltr. | Orchidaceae | Magnoliopsida |
| Diaphananthe sarcorhynchoides J.B.Hall | Orchidaceae | Magnoliopsida |
| Eulophia bouliawongo (Rchb.f.) J.Raynal | Orchidaceae | Magnoliopsida |
| Eulophia caricifolia (Rchb.f.) Summerh. | Orchidaceae | Magnoliopsida |
| Eulophia euglossa (Rchb.f.) Rchb.f. ex Bateman | Orchidaceae | Magnoliopsida |
| Graphorkis lurida (Sw.) Kuntze | Orchidaceae | Magnoliopsida |
| Habenaria physuriformis Kraenzl. | Orchidaceae | Magnoliopsida |
| Habenaria procera (Afzel. ex Sw.) Lindl. | Orchidaceae | Magnoliopsida |
| Habenaria weileriana Schltr. | Orchidaceae | Magnoliopsida |
| Hetaeria heterosepala (Rchb.f.) Summerh. | Orchidaceae | Magnoliopsida |
| Listrostachys pertusa (Lindl.) Rchb.f. | Orchidaceae | Magnoliopsida |
| Phaius mannii Rchb.f. | Orchidaceae | Magnoliopsida |
| Platycoryne buchananiana (Kraenzl.) Rolfe | Orchidaceae | Magnoliopsida |
| Polystachya albescens Ridl. | Orchidaceae | Magnoliopsida |
| Polystachya bifida Lindl. | Orchidaceae | Magnoliopsida |
| Polystachya calluniflora Kraenzl. | Orchidaceae | Magnoliopsida |
| Polystachya dolichophylla Schltr. | Orchidaceae | Magnoliopsida |
| Polystachya pyramidalis Lindl. | Orchidaceae | Magnoliopsida |
| Polystachya ramulosa Lindl. | Orchidaceae | Magnoliopsida |
| Polystachya rhodoptera Rchb.f. | Orchidaceae | Magnoliopsida |
| Polystachya seticaulis Rendle | Orchidaceae | Magnoliopsida |
| Polystachya tessellata Lindl. | Orchidaceae | Magnoliopsida |
| Polystachya testuana Summerh. | Orchidaceae | Magnoliopsida |
| Polystachya victoriae Kraenzl. | Orchidaceae | Magnoliopsida |
| Rangaeris trilobata Summerh. | Orchidaceae | Magnoliopsida |
| Stolzia elaidum (Lindl.) Summerh. | Orchidaceae | Magnoliopsida |
| Tridactyle anthomaniaca (Rchb.f.) Summerh. | Orchidaceae | Magnoliopsida |
| Tridactyle bicaudata (Lindl.) Schltr. | Orchidaceae | Magnoliopsida |
| Tridactyle brevicalcarata Summerh. | Orchidaceae | Magnoliopsida |
| Tridactyle truncatiloba Summerh. | Orchidaceae | Magnoliopsida |
| Vanilla crenulata Rolfe | Orchidaceae | Magnoliopsida |
| Veyretella hetaerioides (Summerh.) Szlach. & Olszewski | Orchidaceae | Magnoliopsida |
| Averrhoa carambola L. | Oxalidaceae | Magnoliopsida |
| Biophytum zenkeri Guillaumin | Oxalidaceae | Magnoliopsida |
| Eremospatha cuspidata (G.Mann & H.Wendl.) H.Wendl. | Palmae | Magnoliopsida |
| Eremospatha laurentii De Wild. | Palmae | Magnoliopsida |
| Hyphaene guineensis Schumach. & Thonn. | Palmae | Magnoliopsida |
| Laccosperma secundiflorum (P.Beauv.) Kuntze | Palmae | Magnoliopsida |
| Oncocalamus mannii (H.Wendl.) H.Wendl. | Palmae | Magnoliopsida |
| Podococcus acaulis Hua | Palmae | Magnoliopsida |
| Podococcus barteri G.Mann & H.Wendl. | Palmae | Magnoliopsida |
| Sclerosperma mannii H.Wendl. | Palmae | Magnoliopsida |
| Microdesmis afrodecandra Floret, A.M.Louis & J.M.Reitsma | Pandaceae | Magnoliopsida |
| Microdesmis klainei J.Léonard | Pandaceae | Magnoliopsida |
| Microdesmis pierlotiana J.Léonard | Pandaceae | Magnoliopsida |
| Microdesmis puberula Hook.f. ex Planch. | Pandaceae | Magnoliopsida |
| Panda oleosa Pierre | Pandaceae | Magnoliopsida |
| Pandanus gabonensis Huynh | Pandanaceae | Magnoliopsida |
| Adenia cissampeloides (Planch. ex Hook.) Harms | Passifloraceae | Magnoliopsida |
| Adenia gracilis Harms | Passifloraceae | Magnoliopsida |
| Adenia reticulata (De Wild. & T.Durand) Engl. | Passifloraceae | Magnoliopsida |
| Barteria fistulosa Mast. | Passifloraceae | Magnoliopsida |
| Barteria nigritana Hook.f. | Passifloraceae | Magnoliopsida |
| Barteria solida Breteler | Passifloraceae | Magnoliopsida |
| Efulensia clematoides C.H.Wright | Passifloraceae | Magnoliopsida |
| Paropsia grewioides Welw. ex Mast. | Passifloraceae | Magnoliopsida |
| Paropsia guineensis Oliv. | Passifloraceae | Magnoliopsida |
| Paropsiopsis decandra (Baill.) Sleumer | Passifloraceae | Magnoliopsida |
| Smeathmannia pubescens Sol. ex R.Br. | Passifloraceae | Magnoliopsida |
| Pentadiplandra brazzeana Baill. | Pentadiplandraceae | Magnoliopsida |
| Peperomia fernandopoiana C.DC. | Piperaceae | Magnoliopsida |
| Peperomia molleri C.DC. | Piperaceae | Magnoliopsida |
| Piper capense L.f. | Piperaceae | Magnoliopsida |
| Piper guineense Schumach. & Thonn. | Piperaceae | Magnoliopsida |
| Piper umbellatum L. | Piperaceae | Magnoliopsida |
| Pittosporum viridiflorum Sims | Pittosporaceae | Magnoliopsida |
| Carpolobia alba G.Don | Polygalaceae | Magnoliopsida |
| Carpolobia gabonica Breteler | Polygalaceae | Magnoliopsida |
| Carpolobia lutea G.Don | Polygalaceae | Magnoliopsida |
| Heterosamara cabrae (Chodat) Paiva | Polygalaceae | Magnoliopsida |
| Heterosamara mannii (Oliv.) Paiva | Polygalaceae | Magnoliopsida |
| Polygala albida Schinz | Polygalaceae | Magnoliopsida |
| Polygala arenaria Willd. | Polygalaceae | Magnoliopsida |
| Polygala ganguelensis Exell & Mendonça | Polygalaceae | Magnoliopsida |
| Polygala lecardii Chodat | Polygalaceae | Magnoliopsida |
| Afrobrunnichia erecta (Asch.) Hutch. & Dalziel | Polygonaceae | Magnoliopsida |
| Drypetes bakembei D.J.Harris & Wortley | Putranjivaceae | Magnoliopsida |
| Drypetes capillipes (Pax) Pax & K.Hoffm. | Putranjivaceae | Magnoliopsida |
| Drypetes chevalieri Beille | Putranjivaceae | Magnoliopsida |
| Drypetes cinnabarina Pax & K.Hoffm. | Putranjivaceae | Magnoliopsida |
| Drypetes gilgiana (Pax) Pax & K.Hoffm. | Putranjivaceae | Magnoliopsida |
| Drypetes gossweileri S.Moore | Putranjivaceae | Magnoliopsida |
| Drypetes inaequalis Hutch. | Putranjivaceae | Magnoliopsida |
| Drypetes magnistipula (Pax) Hutch. | Putranjivaceae | Magnoliopsida |
| Drypetes preussii (Pax) Hutch. | Putranjivaceae | Magnoliopsida |
| Drypetes principum (Müll.Arg.) Hutch. | Putranjivaceae | Magnoliopsida |
| Drypetes verrucosa Hutch. | Putranjivaceae | Magnoliopsida |
| Sibangea arborescens Oliv. | Putranjivaceae | Magnoliopsida |
| Sibangea similis (Hutch.) Radcl.-Sm. | Putranjivaceae | Magnoliopsida |
| Gouania longipetala Hemsl. | Rhamnaceae | Magnoliopsida |
| Lasiodiscus fasciculiflorus Engl. | Rhamnaceae | Magnoliopsida |
| Lasiodiscus mildbraedii Engl. | Rhamnaceae | Magnoliopsida |
| Maesopsis eminii Engl. | Rhamnaceae | Magnoliopsida |
| Cassipourea barteri (Hook.f. ex Oliv.) N.E.Br. | Rhizophoraceae | Magnoliopsida |
| Cassipourea carringtoniana Mendes | Rhizophoraceae | Magnoliopsida |
| Cassipourea ruwensorensis (Engl.) Alston | Rhizophoraceae | Magnoliopsida |
| Cassipourea schizocalyx C.H.Wright | Rhizophoraceae | Magnoliopsida |
| Rhizophora harrisonii Leechm. | Rhizophoraceae | Magnoliopsida |
| Rhizophora racemosa G.Mey. | Rhizophoraceae | Magnoliopsida |
| Rubus pinnatus Willd. | Rosaceae | Magnoliopsida |
| Rubus rosifolius Sm. | Rosaceae | Magnoliopsida |
| Aidia genipiflora (DC.) Dandy | Rubiaceae | Magnoliopsida |
| Aidia micrantha (K.Schum.) Bullock ex F.White | Rubiaceae | Magnoliopsida |
| Aidia ochroleuca (K.Schum.) E.M.A.Petit | Rubiaceae | Magnoliopsida |
| Aidia rhacodosepala (K.Schum.) E.M.A.Petit | Rubiaceae | Magnoliopsida |
| Aidia rubens (Hiern) G.Taylor | Rubiaceae | Magnoliopsida |
| Aoranthe annulata (K.Schum.) Somers | Rubiaceae | Magnoliopsida |
| Aoranthe nalaensis (De Wild.) Somers | Rubiaceae | Magnoliopsida |
| Argocoffeopsis rupestris (Hiern) Robbr. | Rubiaceae | Magnoliopsida |
| Argostemma africanum K.Schum. | Rubiaceae | Magnoliopsida |
| Atractogyne gabonii Pierre | Rubiaceae | Magnoliopsida |
| Aulacocalyx jasminiflora Hook.f. | Rubiaceae | Magnoliopsida |
| Aulacocalyx mapiana Sonké & Bridson | Rubiaceae | Magnoliopsida |
| Aulacocalyx subulata (N.Hallé) Figueiredo | Rubiaceae | Magnoliopsida |
| Aulacocalyx talbotii (Wernham) Keay | Rubiaceae | Magnoliopsida |
| Belonophora coriacea Hoyle | Rubiaceae | Magnoliopsida |
| Belonophora wernhamii Hutch. & Dalziel | Rubiaceae | Magnoliopsida |
| Bertiera aequatorialis N.Hallé | Rubiaceae | Magnoliopsida |
| Bertiera aethiopica Hiern | Rubiaceae | Magnoliopsida |
| Bertiera arctistipula N.Hallé | Rubiaceae | Magnoliopsida |
| Bertiera batesii Wernham | Rubiaceae | Magnoliopsida |
| Bertiera bicarpellata (K.Schum.) N.Hallé | Rubiaceae | Magnoliopsida |
| Bertiera breviflora Hiern | Rubiaceae | Magnoliopsida |
| Bertiera congolana De Wild. & T.Durand | Rubiaceae | Magnoliopsida |
| Bertiera elabensis K.Krause | Rubiaceae | Magnoliopsida |
| Bertiera globiceps K.Schum. | Rubiaceae | Magnoliopsida |
| Bertiera heterophylla Nguembou & Sonké | Rubiaceae | Magnoliopsida |
| Bertiera iturensis K.Krause | Rubiaceae | Magnoliopsida |
| Bertiera laurentii De Wild. | Rubiaceae | Magnoliopsida |
| Bertiera laxa Benth. | Rubiaceae | Magnoliopsida |
| Bertiera lejolyana Nguembou & Sonké | Rubiaceae | Magnoliopsida |
| Bertiera letouzeyi N.Hallé | Rubiaceae | Magnoliopsida |
| Bertiera loraria N.Hallé | Rubiaceae | Magnoliopsida |
| Bertiera lujae De Wild. | Rubiaceae | Magnoliopsida |
| Bertiera naucleoides (S.Moore) Bridson | Rubiaceae | Magnoliopsida |
| Bertiera racemosa (G.Don) K.Schum. | Rubiaceae | Magnoliopsida |
| Bertiera retrofracta K.Schum. | Rubiaceae | Magnoliopsida |
| Bertiera sphaerica N.Hallé | Rubiaceae | Magnoliopsida |
| Bertiera subsessilis Hiern | Rubiaceae | Magnoliopsida |
| Calochone acuminata Keay | Rubiaceae | Magnoliopsida |
| Calycosiphonia spathicalyx (K.Schum.) Robbr. | Rubiaceae | Magnoliopsida |
| Chassalia carvalhoi O.Lachenaud | Rubiaceae | Magnoliopsida |
| Chassalia corallifera (A.Chev. ex De Wild.) Hepper | Rubiaceae | Magnoliopsida |
| Chassalia cristata (Hiern) Bremek. | Rubiaceae | Magnoliopsida |
| Chassalia dictyophylla O.Lachenaud | Rubiaceae | Magnoliopsida |
| Chassalia inflata O.Lachenaud | Rubiaceae | Magnoliopsida |
| Chassalia ischnophylla (K.Schum.) Hepper | Rubiaceae | Magnoliopsida |
| Chassalia lutescens O.Lachenaud & D.J.Harris | Rubiaceae | Magnoliopsida |
| Chassalia macrodiscus K.Schum. | Rubiaceae | Magnoliopsida |
| Chassalia petitiana Piessch. | Rubiaceae | Magnoliopsida |
| Chassalia pteropetala (K.Schum.) Cheek | Rubiaceae | Magnoliopsida |
| Chassalia sosefii O.Lachenaud | Rubiaceae | Magnoliopsida |
| Chassalia subnuda (Hiern) Hepper | Rubiaceae | Magnoliopsida |
| Chassalia tchibangensis Pellegr. | Rubiaceae | Magnoliopsida |
| Chassalia vanderystii (De Wild.) Verdc. | Rubiaceae | Magnoliopsida |
| Chassalia zenkeri K.Schum. & K.Krause | Rubiaceae | Magnoliopsida |
| Chazaliella amplexicaulis O.Lachenaud | Rubiaceae | Magnoliopsida |
| Chazaliella andeliae O.Lachenaud | Rubiaceae | Magnoliopsida |
| Chazaliella coffeosperma (K.Schum.) Verdc. | Rubiaceae | Magnoliopsida |
| Chazaliella domatiicola (De Wild.) E.M.A.Petit & Verdc. | Rubiaceae | Magnoliopsida |
| Chazaliella eriocephala O.Lachenaud | Rubiaceae | Magnoliopsida |
| Chazaliella longistylis (Hiern) E.M.A.Petit & Verdc. | Rubiaceae | Magnoliopsida |
| Chazaliella macrocarpa Verdc. | Rubiaceae | Magnoliopsida |
| Chazaliella obovoidea Verdc. | Rubiaceae | Magnoliopsida |
| Chazaliella oddonii (De Wild.) E.M.A.Petit & Verdc. | Rubiaceae | Magnoliopsida |
| Chazaliella sciadephora (Hiern) E.M.A.Petit & Verdc. | Rubiaceae | Magnoliopsida |
| Chazaliella sclerophylla O.Lachenaud | Rubiaceae | Magnoliopsida |
| Chazaliella subsessilifolia (K.Schum.) O.Lachenaud | Rubiaceae | Magnoliopsida |
| Coffea brevipes Hiern | Rubiaceae | Magnoliopsida |
| Coffea congensis A.Froehner | Rubiaceae | Magnoliopsida |
| Coffea ebracteolata (Hiern) Brenan | Rubiaceae | Magnoliopsida |
| Coffea lebruniana R.Germ. & Kesler | Rubiaceae | Magnoliopsida |
| Coffea magnistipula Stoff. & Robbr. | Rubiaceae | Magnoliopsida |
| Coffea mannii (Hook.f.) A.P.Davis | Rubiaceae | Magnoliopsida |
| Coffea mayombensis A.Chev. | Rubiaceae | Magnoliopsida |
| Colletoecema dewevrei (De Wild.) E.M.A.Petit | Rubiaceae | Magnoliopsida |
| Corynanthe mayumbensis (R.D.Good) Raym.-Hamet ex N.Hallé | Rubiaceae | Magnoliopsida |
| Corynanthe pachyceras K.Schum. | Rubiaceae | Magnoliopsida |
| Corynanthe paniculata Welw. | Rubiaceae | Magnoliopsida |
| Craterispermum aristatum Wernham | Rubiaceae | Magnoliopsida |
| Craterispermum caudatum Hutch. | Rubiaceae | Magnoliopsida |
| Craterispermum cerinanthum Hiern | Rubiaceae | Magnoliopsida |
| Craterispermum gabonicum Taedoumg | Rubiaceae | Magnoliopsida |
| Craterispermum ledermannii K.Krause | Rubiaceae | Magnoliopsida |
| Craterispermum robbrechtianum Taedoumg & Sonké | Rubiaceae | Magnoliopsida |
| Cremaspora thomsonii Hiern | Rubiaceae | Magnoliopsida |
| Cuviera longiflora Hiern | Rubiaceae | Magnoliopsida |
| Cuviera pierrei N.Hallé | Rubiaceae | Magnoliopsida |
| Diodia serrulata (P.Beauv.) G.Taylor | Rubiaceae | Magnoliopsida |
| Diodia vaginalis Benth. | Rubiaceae | Magnoliopsida |
| Ecpoma gigantostipulum (K.Schum.) N.Hallé | Rubiaceae | Magnoliopsida |
| Ecpoma hiernianum (Wernham) N.Hallé & F.Hallé | Rubiaceae | Magnoliopsida |
| Empogona breteleri (Robbr.) Tosh & Robbr. | Rubiaceae | Magnoliopsida |
| Empogona crepiniana (De Wild. & T.Durand) Tosh & Robbr. | Rubiaceae | Magnoliopsida |
| Empogona gossweileri (S.Moore) Tosh & Robbr. | Rubiaceae | Magnoliopsida |
| Euclinia longiflora Salisb. | Rubiaceae | Magnoliopsida |
| Gaertnera bieleri (De Wild.) E.M.A.Petit | Rubiaceae | Magnoliopsida |
| Gaertnera paniculata Benth. | Rubiaceae | Magnoliopsida |
| Gaertnera spicata K.Schum. | Rubiaceae | Magnoliopsida |
| Gaertnera trachystyla (Hiern) E.M.A.Petit | Rubiaceae | Magnoliopsida |
| Gardenia epiphytica Jongkind | Rubiaceae | Magnoliopsida |
| Gardenia nitida Hook. | Rubiaceae | Magnoliopsida |
| Geophila afzelii Hiern | Rubiaceae | Magnoliopsida |
| Geophila lancistipula Hiern | Rubiaceae | Magnoliopsida |
| Geophila obvallata (Schumach.) Didr. | Rubiaceae | Magnoliopsida |
| Globulostylis talbotii Wernham | Rubiaceae | Magnoliopsida |
| Heinsia crinita (Afzel.) G.Taylor | Rubiaceae | Magnoliopsida |
| Heinsia myrmoecia (K.Schum.) N.Hallé | Rubiaceae | Magnoliopsida |
| Heinsia pilosa | Rubiaceae | Magnoliopsida |
| Hekistocarpa minutiflora Hook.f. | Rubiaceae | Magnoliopsida |
| Hymenocoleus barbatus Robbr. | Rubiaceae | Magnoliopsida |
| Hymenocoleus globulifer Robbr. | Rubiaceae | Magnoliopsida |
| Hymenocoleus hirsutus (Benth.) Robbr. | Rubiaceae | Magnoliopsida |
| Hymenocoleus neurodictyon (K.Schum.) Robbr. | Rubiaceae | Magnoliopsida |
| Hymenocoleus physostipula O.Lachenaud | Rubiaceae | Magnoliopsida |
| Hymenodictyon biafranum Hiern | Rubiaceae | Magnoliopsida |
| Ixora aneimenodesma K.Schum. | Rubiaceae | Magnoliopsida |
| Ixora bauchiensis Hutch. & Dalziel | Rubiaceae | Magnoliopsida |
| Ixora brachypoda DC. | Rubiaceae | Magnoliopsida |
| Ixora euosmia K.Schum. | Rubiaceae | Magnoliopsida |
| Ixora guineensis Benth. | Rubiaceae | Magnoliopsida |
| Ixora hiernii Scott-Elliot | Rubiaceae | Magnoliopsida |
| Ixora hippoperifera Bremek. | Rubiaceae | Magnoliopsida |
| Ixora inundata Hiern | Rubiaceae | Magnoliopsida |
| Ixora laurentii De Wild. | Rubiaceae | Magnoliopsida |
| Ixora macilenta De Block | Rubiaceae | Magnoliopsida |
| Ixora minutiflora Hiern | Rubiaceae | Magnoliopsida |
| Ixora nematopoda K.Schum. | Rubiaceae | Magnoliopsida |
| Ixora praetermissa De Block | Rubiaceae | Magnoliopsida |
| Keetia multiflora (Schumach. & Thonn.) Bridson | Rubiaceae | Magnoliopsida |
| Kohautia tenuis (Bowdich) Mabb. | Rubiaceae | Magnoliopsida |
| Lasianthus africanus Hiern | Rubiaceae | Magnoliopsida |
| Lasianthus batangensis K.Schum. | Rubiaceae | Magnoliopsida |
| Lasianthus mayumbensis R.D.Good | Rubiaceae | Magnoliopsida |
| Lasianthus repens Hepper | Rubiaceae | Magnoliopsida |
| Lasianthus urophylloides R.D.Good | Rubiaceae | Magnoliopsida |
| Leptactina involucrata Hook.f. | Rubiaceae | Magnoliopsida |
| Leptactina latifolia K.Schum. | Rubiaceae | Magnoliopsida |
| Leptactina leopoldi-secundi Büttner | Rubiaceae | Magnoliopsida |
| Leptactina mannii Hook.f. | Rubiaceae | Magnoliopsida |
| Leptactina pynaertii De Wild. | Rubiaceae | Magnoliopsida |
| Massularia acuminata (G.Don) Bullock ex Hoyle | Rubiaceae | Magnoliopsida |
| Morinda longiflora G.Don | Rubiaceae | Magnoliopsida |
| Morinda morindoides (Baker) Milne-Redh. | Rubiaceae | Magnoliopsida |
| Morinda titanophylla E.M.A.Petit | Rubiaceae | Magnoliopsida |
| Mussaenda debeauxii Wernham | Rubiaceae | Magnoliopsida |
| Mussaenda elegans Schumach. & Thonn. | Rubiaceae | Magnoliopsida |
| Mussaenda erythrophylla Schumach. & Thonn. | Rubiaceae | Magnoliopsida |
| Mussaenda isertiana DC. | Rubiaceae | Magnoliopsida |
| Mussaenda nannanii Wernham | Rubiaceae | Magnoliopsida |
| Mussaenda polita Hiern | Rubiaceae | Magnoliopsida |
| Mussaenda soyauxii Büttner | Rubiaceae | Magnoliopsida |
| Nauclea diderrichii (De Wild.) Merr. | Rubiaceae | Magnoliopsida |
| Nauclea gilletii (De Wild.) Merr. | Rubiaceae | Magnoliopsida |
| Nichallea soyauxii (Hiern) Bridson | Rubiaceae | Magnoliopsida |
| Otiophora scabra Zucc. | Rubiaceae | Magnoliopsida |
| Otomeria cameronica (Bremek.) Hepper | Rubiaceae | Magnoliopsida |
| Otomeria guineensis Benth. | Rubiaceae | Magnoliopsida |
| Otomeria micrantha K.Schum. | Rubiaceae | Magnoliopsida |
| Oxyanthus dubius De Wild. | Rubiaceae | Magnoliopsida |
| Oxyanthus formosus Hook.f. ex Planch. | Rubiaceae | Magnoliopsida |
| Oxyanthus laxiflorus K.Schum. ex Hutch. & Dalziel | Rubiaceae | Magnoliopsida |
| Oxyanthus pallidus Hiern | Rubiaceae | Magnoliopsida |
| Oxyanthus schumannianus De Wild. & T.Durand | Rubiaceae | Magnoliopsida |
| Oxyanthus setosus Keay | Rubiaceae | Magnoliopsida |
| Oxyanthus subpunctatus (Hiern) Keay | Rubiaceae | Magnoliopsida |
| Parapentas setigera (Hiern) Verdc. | Rubiaceae | Magnoliopsida |
| Pauridiantha callicarpoides (Hiern) Bremek. | Rubiaceae | Magnoliopsida |
| Pauridiantha crystallina (N.Hallé) Smedmark & B.Bremer | Rubiaceae | Magnoliopsida |
| Pauridiantha dewevrei (De Wild. & T.Durand) Bremek. | Rubiaceae | Magnoliopsida |
| Pauridiantha floribunda (K.Schum. & K.Krause) Bremek. | Rubiaceae | Magnoliopsida |
| Pauridiantha le-testuana (N.Hallé) Ntore & Dessein | Rubiaceae | Magnoliopsida |
| Pauridiantha liebrechtsiana (De Wild. & T.Durand) Ntore & Dessein | Rubiaceae | Magnoliopsida |
| Pauridiantha mayumbensis (R.D.Good) Bremek. | Rubiaceae | Magnoliopsida |
| Pauridiantha micrantha (Hiern) Bremek. | Rubiaceae | Magnoliopsida |
| Pauridiantha multiflora K.Schum. | Rubiaceae | Magnoliopsida |
| Pauridiantha pleiantha Ntore & Dessein | Rubiaceae | Magnoliopsida |
| Pauridiantha pyramidata (K.Krause) Bremek. | Rubiaceae | Magnoliopsida |
| Pauridiantha smetsiana Ntore & Dessein | Rubiaceae | Magnoliopsida |
| Pauridiantha talbotii (Wernham) Ntore & Dessein | Rubiaceae | Magnoliopsida |
| Pauridiantha triflora Ntore & Dessein | Rubiaceae | Magnoliopsida |
| Pausinystalia macroceras (K.Schum.) Pierre | Rubiaceae | Magnoliopsida |
| Pausinystalia talbotii Wernham | Rubiaceae | Magnoliopsida |
| Pavetta batesiana Bremek. | Rubiaceae | Magnoliopsida |
| Pavetta bidentata Hiern | Rubiaceae | Magnoliopsida |
| Pavetta camerounensis S.D.Manning | Rubiaceae | Magnoliopsida |
| Pavetta corymbosa (DC.) F.N.Williams | Rubiaceae | Magnoliopsida |
| Pavetta gabonica Bremek. | Rubiaceae | Magnoliopsida |
| Pavetta hispida Hiern | Rubiaceae | Magnoliopsida |
| Pavetta kribiensis S.D.Manning | Rubiaceae | Magnoliopsida |
| Pavetta laurentii De Wild. | Rubiaceae | Magnoliopsida |
| Pavetta muiriana S.D.Manning | Rubiaceae | Magnoliopsida |
| Pavetta neurocarpa Benth. | Rubiaceae | Magnoliopsida |
| Pavetta owariensis P.Beauv. | Rubiaceae | Magnoliopsida |
| Pavetta rigida Hiern | Rubiaceae | Magnoliopsida |
| Pavetta spathulata Bremek. | Rubiaceae | Magnoliopsida |
| Pavetta tetramera (Hiern) Bremek. | Rubiaceae | Magnoliopsida |
| Pavetta viridiloba K.Krause | Rubiaceae | Magnoliopsida |
| Pentaloncha humilis Hook.f. | Rubiaceae | Magnoliopsida |
| Petitiocodon parviflorum (Keay) Robbr. | Rubiaceae | Magnoliopsida |
| Pleiocoryne fernandensis (Hiern) Rauschert | Rubiaceae | Magnoliopsida |
| Poecilocalyx crystallinus N.Hallé | Rubiaceae | Magnoliopsida |
| Poecilocalyx schumannii Bremek. | Rubiaceae | Magnoliopsida |
| Poecilocalyx stipulosa (Hutch. & Dalziel) N.Hallé | Rubiaceae | Magnoliopsida |
| Polysphaeria macrophylla K.Schum. | Rubiaceae | Magnoliopsida |
| Pouchetia baumanniana Büttner | Rubiaceae | Magnoliopsida |
| Pseudosabicea aurifodinae N.Hallé | Rubiaceae | Magnoliopsida |
| Pseudosabicea batesii (Wernham) N.Hallé | Rubiaceae | Magnoliopsida |
| Pseudosabicea floribunda (K.Schum.) N.Hallé | Rubiaceae | Magnoliopsida |
| Pseudosabicea medusula (K.Schum. ex Wernham) N.Hallé | Rubiaceae | Magnoliopsida |
| Pseudosabicea mildbraedii (Wernham) N.Hallé | Rubiaceae | Magnoliopsida |
| Pseudosabicea nobilis (R.D.Good) N.Hallé | Rubiaceae | Magnoliopsida |
| Pseudosabicea proselyta N.Hallé | Rubiaceae | Magnoliopsida |
| Pseudosabicea sanguinosa N.Hallé | Rubiaceae | Magnoliopsida |
| Pseudosabicea segregata (Hiern) N.Hallé | Rubiaceae | Magnoliopsida |
| Pseudosabicea sthenula N.Hallé | Rubiaceae | Magnoliopsida |
| Psychotria acutigemma O.Lachenaud | Rubiaceae | Magnoliopsida |
| Psychotria alatipes Wernham | Rubiaceae | Magnoliopsida |
| Psychotria anthocleistifolia O.Lachenaud | Rubiaceae | Magnoliopsida |
| Psychotria arborea Hiern | Rubiaceae | Magnoliopsida |
| Psychotria arnoldiana De Wild. | Rubiaceae | Magnoliopsida |
| Psychotria avakubiensis De Wild. | Rubiaceae | Magnoliopsida |
| Psychotria bifaria Hiern | Rubiaceae | Magnoliopsida |
| Psychotria brachyantha Hiern | Rubiaceae | Magnoliopsida |
| Psychotria brachypus (K.Schum. & K.Krause) O.Lachenaud | Rubiaceae | Magnoliopsida |
| Psychotria bracteosa Hiern [1] | Rubiaceae | Magnoliopsida |
| Psychotria breteleri O.Lachenaud | Rubiaceae | Magnoliopsida |
| Psychotria brevifissa O.Lachenaud | Rubiaceae | Magnoliopsida |
| Psychotria brieyi De Wild. | Rubiaceae | Magnoliopsida |
| Psychotria butayei De Wild. | Rubiaceae | Magnoliopsida |
| Psychotria calceata E.M.A.Petit | Rubiaceae | Magnoliopsida |
| Psychotria camptopus Verdc. | Rubiaceae | Magnoliopsida |
| Psychotria chalconeura (K.Schum.) E.M.A.Petit | Rubiaceae | Magnoliopsida |
| Psychotria clausa (Hiern) O.Lachenaud | Rubiaceae | Magnoliopsida |
| Psychotria cyanopharynx K.Schum. | Rubiaceae | Magnoliopsida |
| Psychotria dermatophylla (K.Schum.) E.M.A.Petit | Rubiaceae | Magnoliopsida |
| Psychotria desseinii O.Lachenaud | Rubiaceae | Magnoliopsida |
| Psychotria dewildei O.Lachenaud | Rubiaceae | Magnoliopsida |
| Psychotria dorotheae Wernham | Rubiaceae | Magnoliopsida |
| Psychotria ebensis K.Schum. | Rubiaceae | Magnoliopsida |
| Psychotria eladii O.Lachenaud | Rubiaceae | Magnoliopsida |
| Psychotria fimbriatifolia R.D.Good | Rubiaceae | Magnoliopsida |
| Psychotria flagelliflora O.Lachenaud | Rubiaceae | Magnoliopsida |
| Psychotria foliosa Hiern | Rubiaceae | Magnoliopsida |
| Psychotria gabonica Hiern | Rubiaceae | Magnoliopsida |
| Psychotria gilletii De Wild. | Rubiaceae | Magnoliopsida |
| Psychotria globiceps K.Schum. | Rubiaceae | Magnoliopsida |
| Psychotria globosa Hiern | Rubiaceae | Magnoliopsida |
| Psychotria guineensis E.M.A.Petit | Rubiaceae | Magnoliopsida |
| Psychotria hedraeocephala (Bremek.) O.Lachenaud | Rubiaceae | Magnoliopsida |
| Psychotria hemistegia O.Lachenaud | Rubiaceae | Magnoliopsida |
| Psychotria hexamera (K.Schum.) O.Lachenaud | Rubiaceae | Magnoliopsida |
| Psychotria humifera O.Lachenaud | Rubiaceae | Magnoliopsida |
| Psychotria hypsophila K.Schum. & K.Krause | Rubiaceae | Magnoliopsida |
| Psychotria ingentifolia E.M.A.Petit | Rubiaceae | Magnoliopsida |
| Psychotria issembei O.Lachenaud | Rubiaceae | Magnoliopsida |
| Psychotria ituriensis De Wild. ex E.M.A.Petit | Rubiaceae | Magnoliopsida |
| Psychotria konguensis Hiern | Rubiaceae | Magnoliopsida |
| Psychotria lanceifolia K.Schum. | Rubiaceae | Magnoliopsida |
| Psychotria laticalyx O.Lachenaud | Rubiaceae | Magnoliopsida |
| Psychotria latistipula Benth. | Rubiaceae | Magnoliopsida |
| Psychotria laurentii De Wild. | Rubiaceae | Magnoliopsida |
| Psychotria laxithyrsa O.Lachenaud | Rubiaceae | Magnoliopsida |
| Psychotria le-testui (De Wild.) N.Hallé ex O.Lachenaud | Rubiaceae | Magnoliopsida |
| Psychotria ledermannii (K.Krause) Figueiredo | Rubiaceae | Magnoliopsida |
| Psychotria letouzeyi E.M.A.Petit | Rubiaceae | Magnoliopsida |
| Psychotria mannii Hiern | Rubiaceae | Magnoliopsida |
| Psychotria oblanceolata (R.D.Good) Ruhsam | Rubiaceae | Magnoliopsida |
| Psychotria peduncularis (Salisb.) Steyerm. | Rubiaceae | Magnoliopsida |
| Psychotria pendulothyrsa O.Lachenaud | Rubiaceae | Magnoliopsida |
| Psychotria podocarpa E.M.A.Petit | Rubiaceae | Magnoliopsida |
| Psychotria potanthera Wernham | Rubiaceae | Magnoliopsida |
| Psychotria radicifera O.Lachenaud | Rubiaceae | Magnoliopsida |
| Psychotria rambouensis De Wild. | Rubiaceae | Magnoliopsida |
| Psychotria raynaliorum O.Lachenaud | Rubiaceae | Magnoliopsida |
| Psychotria reitsmarum O.Lachenaud | Rubiaceae | Magnoliopsida |
| Psychotria rhynchodiscus O.Lachenaud | Rubiaceae | Magnoliopsida |
| Psychotria rubescens (Hiern) O.Lachenaud | Rubiaceae | Magnoliopsida |
| Psychotria rubristipulata R.D.Good | Rubiaceae | Magnoliopsida |
| Psychotria rufipilis A.Chev. ex De Wild. | Rubiaceae | Magnoliopsida |
| Psychotria sadebeckiana K.Schum. | Rubiaceae | Magnoliopsida |
| Psychotria senterrei O.Lachenaud | Rubiaceae | Magnoliopsida |
| Psychotria sitae O.Lachenaud | Rubiaceae | Magnoliopsida |
| Psychotria solfiana K.Krause | Rubiaceae | Magnoliopsida |
| Psychotria stenostegia O.Lachenaud | Rubiaceae | Magnoliopsida |
| Psychotria subobliqua Hiern | Rubiaceae | Magnoliopsida |
| Psychotria succulenta (Schweinf. ex Hiern) E.M.A.Petit | Rubiaceae | Magnoliopsida |
| Psychotria synactica O.Lachenaud | Rubiaceae | Magnoliopsida |
| Psychotria thonneri (De Wild. & T.Durand) O.Lachenaud | Rubiaceae | Magnoliopsida |
| Psychotria uapacifolia O.Lachenaud | Rubiaceae | Magnoliopsida |
| Psychotria varians O.Lachenaud | Rubiaceae | Magnoliopsida |
| Psychotria villicarpa O.Lachenaud | Rubiaceae | Magnoliopsida |
| Psychotria vogeliana Benth. | Rubiaceae | Magnoliopsida |
| Psychotria wieringae O.Lachenaud | Rubiaceae | Magnoliopsida |
| Psydrax horizontalis (Schumach. & Thonn.) Bridson | Rubiaceae | Magnoliopsida |
| Psydrax moandensis Bridson | Rubiaceae | Magnoliopsida |
| Rothmannia hispida (K.Schum.) Fagerl. | Rubiaceae | Magnoliopsida |
| Rothmannia lateriflora (K.Schum.) Keay | Rubiaceae | Magnoliopsida |
| Rothmannia libisa N.Hallé | Rubiaceae | Magnoliopsida |
| Rothmannia liebrechtsiana (De Wild. & T.Durand) Keay | Rubiaceae | Magnoliopsida |
| Rothmannia lujae (De Wild.) Keay | Rubiaceae | Magnoliopsida |
| Rutidea decorticata Hiern | Rubiaceae | Magnoliopsida |
| Rutidea ferruginea Hiern | Rubiaceae | Magnoliopsida |
| Rutidea glabra Hiern | Rubiaceae | Magnoliopsida |
| Rutidea hispida Hiern | Rubiaceae | Magnoliopsida |
| Rutidea membranacea Hiern | Rubiaceae | Magnoliopsida |
| Rutidea parviflora DC. | Rubiaceae | Magnoliopsida |
| Rutidea rufipilis Hiern | Rubiaceae | Magnoliopsida |
| Rytigynia canthioides (Benth.) Robyns | Rubiaceae | Magnoliopsida |
| Rytigynia robusta O.Lachenaud | Rubiaceae | Magnoliopsida |
| Sabicea bigerrica N.Hallé | Rubiaceae | Magnoliopsida |
| Sabicea calycina Benth. | Rubiaceae | Magnoliopsida |
| Sabicea caminata N.Hallé | Rubiaceae | Magnoliopsida |
| Sabicea dinklagei K.Schum. | Rubiaceae | Magnoliopsida |
| Sabicea duparquetiana Baill. ex Wernham | Rubiaceae | Magnoliopsida |
| Sabicea fulva Wernham | Rubiaceae | Magnoliopsida |
| Sabicea fulvilipis Zemagho | Rubiaceae | Magnoliopsida |
| Sabicea pilosa Hiern | Rubiaceae | Magnoliopsida |
| Sabicea rufa Wernham | Rubiaceae | Magnoliopsida |
| Sabicea speciosa K.Schum. | Rubiaceae | Magnoliopsida |
| Schizocolea ochreata E.M.A.Petit | Rubiaceae | Magnoliopsida |
| Sericanthe auriculata (Keay) Robbr. | Rubiaceae | Magnoliopsida |
| Sericanthe gabonensis Sonké & Robbr. | Rubiaceae | Magnoliopsida |
| Sericanthe jacfelicis (N.Hallé) Robbr. | Rubiaceae | Magnoliopsida |
| Sericanthe pellegrinii (N.Hallé) Robbr. | Rubiaceae | Magnoliopsida |
| Sericanthe testui (N.Hallé) Robbr. | Rubiaceae | Magnoliopsida |
| Sherbournia ailarama N.Hallé | Rubiaceae | Magnoliopsida |
| Sherbournia batesii (Wernham) Hepper | Rubiaceae | Magnoliopsida |
| Sherbournia streptocaulon (K.Schum.) Hepper | Rubiaceae | Magnoliopsida |
| Sherbournia zenkeri Hua | Rubiaceae | Magnoliopsida |
| Spermacoce hepperana Verdc. | Rubiaceae | Magnoliopsida |
| Stelechantha cauliflora (R.D.Good) Bremek. | Rubiaceae | Magnoliopsida |
| Stelechantha makakana N.Hallé | Rubiaceae | Magnoliopsida |
| Stipularia africana P.Beauv. | Rubiaceae | Magnoliopsida |
| Stipularia elliptica Schweinf. ex Hiern | Rubiaceae | Magnoliopsida |
| Tarenna bipindensis (K.Schum.) Bremek. | Rubiaceae | Magnoliopsida |
| Tarenna eketensis Wernham | Rubiaceae | Magnoliopsida |
| Tarenna fusco-flava (K.Schum.) S.Moore | Rubiaceae | Magnoliopsida |
| Tarenna grandiflora (Benth.) Hiern | Rubiaceae | Magnoliopsida |
| Tarenna jolinonii N.Hallé | Rubiaceae | Magnoliopsida |
| Tarenna precidantenna N.Hallé | Rubiaceae | Magnoliopsida |
| Temnopteryx sericea Hook.f. | Rubiaceae | Magnoliopsida |
| Tricalysia elliotii (K.Schum.) Hutch. & Dalziel | Rubiaceae | Magnoliopsida |
| Tricalysia ferorum Robbr. | Rubiaceae | Magnoliopsida |
| Tricalysia hensii De Wild. | Rubiaceae | Magnoliopsida |
| Tricalysia obstetrix N.Hallé | Rubiaceae | Magnoliopsida |
| Tricalysia okelensis Hiern | Rubiaceae | Magnoliopsida |
| Tricalysia pallens Hiern | Rubiaceae | Magnoliopsida |
| Tricalysia pedunculosa (N.Hallé) Robbr. | Rubiaceae | Magnoliopsida |
| Tricalysia sylvae Robbr. | Rubiaceae | Magnoliopsida |
| Trichostachys aurea Hiern | Rubiaceae | Magnoliopsida |
| Trichostachys longifolia Hiern | Rubiaceae | Magnoliopsida |
| Trichostachys microcarpa K.Schum. | Rubiaceae | Magnoliopsida |
| Uncaria africana G.Don | Rubiaceae | Magnoliopsida |
| Vangueriella chlorantha (K.Schum.) Verdc. | Rubiaceae | Magnoliopsida |
| Vangueriella laxiflora (K.Schum.) Verdc. | Rubiaceae | Magnoliopsida |
| Vangueriella olacifolia (Robyns) Verdc. | Rubiaceae | Magnoliopsida |
| Vangueriella orthacantha (Mildbr.) Bridson & Verdc. | Rubiaceae | Magnoliopsida |
| Vangueriella soyauxii (K.Schum.) Verdc. | Rubiaceae | Magnoliopsida |
| Virectaria angustifolia (Hiern) Bremek. | Rubiaceae | Magnoliopsida |
| Virectaria belingana N.Hallé | Rubiaceae | Magnoliopsida |
| Virectaria major (K.Schum.) Verdc. | Rubiaceae | Magnoliopsida |
| Virectaria procumbens (Sm.) Bremek. | Rubiaceae | Magnoliopsida |
| Afraegle paniculata (Schumach. & Thonn.) Engl. | Rutaceae | Magnoliopsida |
| Citropsis gabunensis (Engl.) Swingle & M.Kellerm. | Rutaceae | Magnoliopsida |
| Vepris glaberrima (Engl.) J.B.Hall ex D.J.Harris | Rutaceae | Magnoliopsida |
| Zanthoxylum buesgenii (Engl.) P.G.Waterman | Rutaceae | Magnoliopsida |
| Viscum congolense De Wild. | Santalaceae | Magnoliopsida |
| Allophylus ferrugineus Taub. | Sapindaceae | Magnoliopsida |
| Allophylus grandifolius (Baker) Radlk. | Sapindaceae | Magnoliopsida |
| Allophylus hallei Fouilloy | Sapindaceae | Magnoliopsida |
| Allophylus hirtellus (Hook.f.) Radlk. | Sapindaceae | Magnoliopsida |
| Allophylus megaphyllus Hutch. & Dalziel | Sapindaceae | Magnoliopsida |
| Allophylus spicatus (Poir.) Radlk. | Sapindaceae | Magnoliopsida |
| Aporrhiza talbotii Baker f. | Sapindaceae | Magnoliopsida |
| Aporrhiza urophylla Gilg | Sapindaceae | Magnoliopsida |
| Blighia sapida K.D.Koenig | Sapindaceae | Magnoliopsida |
| Chytranthus angustifolius Exell | Sapindaceae | Magnoliopsida |
| Chytranthus cauliflorus (Hutch. & Dalziel) Wickens | Sapindaceae | Magnoliopsida |
| Chytranthus gilletii De Wild. | Sapindaceae | Magnoliopsida |
| Deinbollia mezilii D.W.Thomas & D.J.Harris | Sapindaceae | Magnoliopsida |
| Deinbollia pinnata (Poir.) Schumach. & Thonn. | Sapindaceae | Magnoliopsida |
| Deinbollia unijuga D.W.Thomas | Sapindaceae | Magnoliopsida |
| Eriocoelum petiolare Radlk. | Sapindaceae | Magnoliopsida |
| Ganophyllum giganteum (A.Chev.) Hauman | Sapindaceae | Magnoliopsida |
| Laccodiscus ferrugineus (Baker) Radlk. | Sapindaceae | Magnoliopsida |
| Laccodiscus klaineanus Pierre ex Engl. | Sapindaceae | Magnoliopsida |
| Pancovia floribunda Pellegr. | Sapindaceae | Magnoliopsida |
| Placodiscus caudatus Pierre ex Radlk. | Sapindaceae | Magnoliopsida |
| Breviea sericea Aubrév. & Pellegr. | Sapotaceae | Magnoliopsida |
| Delpydora macrophylla Pierre | Sapotaceae | Magnoliopsida |
| Donella ogoouensis (A.Chev.) Aubrév. & Pellegr. | Sapotaceae | Magnoliopsida |
| Englerophytum stelechantha K.Krause | Sapotaceae | Magnoliopsida |
| Gambeya africana (A.DC.) Pierre | Sapotaceae | Magnoliopsida |
| Gambeya lacourtiana (De Wild.) Aubrév. & Pellegr. | Sapotaceae | Magnoliopsida |
| Gambeya perpulchra (Mildbr. ex Hutch. & Dalziel) Aubrév. & Pellegr. | Sapotaceae | Magnoliopsida |
| Ituridendron bequaertii De Wild. | Sapotaceae | Magnoliopsida |
| Lecomtedoxa biraudii Aubrév. & Pellegr. | Sapotaceae | Magnoliopsida |
| Lecomtedoxa klaineana (Pierre ex Engl.) Pierre ex Dubard | Sapotaceae | Magnoliopsida |
| Lecomtedoxa nogo (A.Chev.) Aubrév. | Sapotaceae | Magnoliopsida |
| Manilkara cuneifolia (Baker) Dubard | Sapotaceae | Magnoliopsida |
| Manilkara fouilloyana Aubrév. & Pellegr. | Sapotaceae | Magnoliopsida |
| Manilkara welwitschii (Engl.) Dubard | Sapotaceae | Magnoliopsida |
| Omphalocarpum elatum Miers | Sapotaceae | Magnoliopsida |
| Omphalocarpum procerum P.Beauv. | Sapotaceae | Magnoliopsida |
| Synsepalum fleuryanum A.Chev. | Sapotaceae | Magnoliopsida |
| Synsepalum msolo (Engl.) T.D.Penn. | Sapotaceae | Magnoliopsida |
| Synsepalum passargei (Engl.) T.D.Penn. | Sapotaceae | Magnoliopsida |
| Tieghemella africana Pierre | Sapotaceae | Magnoliopsida |
| Zeyherella mayumbensis (Greves) Aubrév. & Pellegr. | Sapotaceae | Magnoliopsida |
| Alectra glandulosa Philcox | Scrophulariaceae | Magnoliopsida |
| Bacopa floribunda (R.Br.) Wettst. | Scrophulariaceae | Magnoliopsida |
| Limnophila barteri Skan | Scrophulariaceae | Magnoliopsida |
| Striga bilabiata (Thunb.) Kuntze | Scrophulariaceae | Magnoliopsida |
| Brazzeia congoensis Baill. | Scytopetalaceae | Magnoliopsida |
| Brazzeia soyauxii (Oliv.) Tiegh. | Scytopetalaceae | Magnoliopsida |
| Oubanguia africana Baill. | Scytopetalaceae | Magnoliopsida |
| Oubanguia alata Baker f. | Scytopetalaceae | Magnoliopsida |
| Pierrina zenkeri Engl. | Scytopetalaceae | Magnoliopsida |
| Scytopetalum klaineanum Pierre | Scytopetalaceae | Magnoliopsida |
| Odyendyea gabonensis (Pierre) Engl. | Simaroubaceae | Magnoliopsida |
| Quassia africana (Baill.) Baill. | Simaroubaceae | Magnoliopsida |
| Physalis peruviana L. | Solanaceae | Magnoliopsida |
| Solanum aculeastrum Dunal | Solanaceae | Magnoliopsida |
| Solanum anguivi species group | Solanaceae | Magnoliopsida |
| Solanum torvum Sw. | Solanaceae | Magnoliopsida |
| Sphenoclea zeylanica Gaertn. | Sphenocleaceae | Magnoliopsida |
| Chlamydocola chlamydantha (K.Schum.) M.Bodard | Sterculiaceae | Magnoliopsida |
| Chlamydocola lastoursvillensis (M.Bodard & Pellegr.) N.Hallé | Sterculiaceae | Magnoliopsida |
| Cola acuminata (P.Beauv.) Schott & Endl. | Sterculiaceae | Magnoliopsida |
| Cola argentea Mast. | Sterculiaceae | Magnoliopsida |
| Cola ballayi Cornu ex Heckel | Sterculiaceae | Magnoliopsida |
| Cola cauliflora Mast. | Sterculiaceae | Magnoliopsida |
| Cola digitata Mast. | Sterculiaceae | Magnoliopsida |
| Cola ficifolia Mast. | Sterculiaceae | Magnoliopsida |
| Cola flavovelutina K.Schum. | Sterculiaceae | Magnoliopsida |
| Cola gabonensis Mast. | Sterculiaceae | Magnoliopsida |
| Cola heterophylla (P.Beauv.) Schott & Endl. | Sterculiaceae | Magnoliopsida |
| Cola lateritia K.Schum. | Sterculiaceae | Magnoliopsida |
| Cola marsupium K.Schum. | Sterculiaceae | Magnoliopsida |
| Cola mayimbensis Pellegr. | Sterculiaceae | Magnoliopsida |
| Cola micrantha K.Schum. | Sterculiaceae | Magnoliopsida |
| Cola pachycarpa K.Schum. | Sterculiaceae | Magnoliopsida |
| Cola semecarpophylla K.Schum. | Sterculiaceae | Magnoliopsida |
| Leptonychia batangensis (C.H.Wright) Burret | Sterculiaceae | Magnoliopsida |
| Leptonychia echinocarpa K.Schum. | Sterculiaceae | Magnoliopsida |
| Leptonychia lasiogyne K.Schum. | Sterculiaceae | Magnoliopsida |
| Leptonychia multiflora K.Schum. | Sterculiaceae | Magnoliopsida |
| Megatritheca grossedenticulata (M.Bodard & Pellegr.) Cristóbal | Sterculiaceae | Magnoliopsida |
| Nesogordonia kabingaensis (K.Schum.) Capuron ex R.Germ. | Sterculiaceae | Magnoliopsida |
| Octolobus spectabilis Welw. | Sterculiaceae | Magnoliopsida |
| Scaphopetalum acuminatum Engl. & K.Krause | Sterculiaceae | Magnoliopsida |
| Scaphopetalum blackii Mast. | Sterculiaceae | Magnoliopsida |
| Scaphopetalum macranthum K.Schum. | Sterculiaceae | Magnoliopsida |
| Scaphopetalum mannii Mast. | Sterculiaceae | Magnoliopsida |
| Scaphopetalum riparium Engl. & K.Krause | Sterculiaceae | Magnoliopsida |
| Scaphopetalum thonneri De Wild. & T.Durand | Sterculiaceae | Magnoliopsida |
| Scaphopetalum zenkeri K.Schum. | Sterculiaceae | Magnoliopsida |
| Sterculia oblonga Mast. | Sterculiaceae | Magnoliopsida |
| Sterculia tragacantha Lindl. | Sterculiaceae | Magnoliopsida |
| Triplochiton scleroxylon K.Schum. | Sterculiaceae | Magnoliopsida |
| Ternstroemia africana Melch. | Theaceae | Magnoliopsida |
| Thomandersia butayei De Wild. | Thomandersiaceae | Magnoliopsida |
| Thomandersia congolana De Wild. & T.Durand | Thomandersiaceae | Magnoliopsida |
| Thomandersia hensii De Wild. & T.Durand | Thomandersiaceae | Magnoliopsida |
| Thomandersia laurifolia (T.Anderson ex Benth.) Baill. | Thomandersiaceae | Magnoliopsida |
| Dicranolepis baertsiana De Wild. & T.Durand | Thymelaeaceae | Magnoliopsida |
| Dicranolepis buchholzii Engl. & Gilg | Thymelaeaceae | Magnoliopsida |
| Dicranolepis disticha Planch. | Thymelaeaceae | Magnoliopsida |
| Dicranolepis glandulosa H.Pearson | Thymelaeaceae | Magnoliopsida |
| Dicranolepis grandiflora Engl. | Thymelaeaceae | Magnoliopsida |
| Dicranolepis pulcherrima Gilg | Thymelaeaceae | Magnoliopsida |
| Dicranolepis soyauxii Engl. | Thymelaeaceae | Magnoliopsida |
| Dicranolepis vestita Engl. | Thymelaeaceae | Magnoliopsida |
| Octolepis casearia Oliv. | Thymelaeaceae | Magnoliopsida |
| Peddiea africana Harv. | Thymelaeaceae | Magnoliopsida |
| Clappertonia ficifolia (Willd.) Decne. | Tiliaceae | Magnoliopsida |
| Clappertonia polyandra (K.Schum. ex Sprague) Bech. | Tiliaceae | Magnoliopsida |
| Desplatsia chrysochlamys (Mildbr. & Burret) Mildbr. & Burret | Tiliaceae | Magnoliopsida |
| Desplatsia subericarpa Bocq. | Tiliaceae | Magnoliopsida |
| Glyphaea brevis (Spreng.) Monach. | Tiliaceae | Magnoliopsida |
| Grewia carpinifolia Juss. | Tiliaceae | Magnoliopsida |
| Grewia malacocarpa Mast. | Tiliaceae | Magnoliopsida |
| Microcos coriacea (Mast.) Burret | Tiliaceae | Magnoliopsida |
| Microcos oligoneura (Sprague) Burret | Tiliaceae | Magnoliopsida |
| Microcos pinnatifida (Mast.) Burret | Tiliaceae | Magnoliopsida |
| Sciaphila ledermannii Engl. | Triuridaceae | Magnoliopsida |
| Celtis adolfi-friderici Engl. | Ulmaceae | Magnoliopsida |
| Celtis africana Burm.f. | Ulmaceae | Magnoliopsida |
| Celtis tessmannii Rendle | Ulmaceae | Magnoliopsida |
| Celtis toka (Forssk.) Hepper & J.R.I.Wood | Ulmaceae | Magnoliopsida |
| Diplolophium africanum Turcz. | Umbelliferae | Magnoliopsida |
| Eryngium foetidum L. | Umbelliferae | Magnoliopsida |
| Hydrocotyle bonariensis Lam. | Umbelliferae | Magnoliopsida |
| Boehmeria macrophylla Hornem. | Urticaceae | Magnoliopsida |
| Elatostema monticola Hook.f. | Urticaceae | Magnoliopsida |
| Elatostema paivaeanum Wedd. | Urticaceae | Magnoliopsida |
| Laportea ovalifolia (Schumach. & Thonn.) Chew | Urticaceae | Magnoliopsida |
| Pilea sublucens Wedd. | Urticaceae | Magnoliopsida |
| Procris crenata C.B.Rob. | Urticaceae | Magnoliopsida |
| Urera gabonensis Pierre | Urticaceae | Magnoliopsida |
| Urera trinervis (Hochst.) Friis & Immelman | Urticaceae | Magnoliopsida |
| Stachytarpheta cayennensis (Rich.) Vahl | Verbenaceae | Magnoliopsida |
| Stachytarpheta indica (L.) Vahl | Verbenaceae | Magnoliopsida |
| Allexis cauliflora (Oliv.) Pierre | Violaceae | Magnoliopsida |
| Rinorea angustifolia (Thouars) Baill. | Violaceae | Magnoliopsida |
| Rinorea apertior Achound. & Bos | Violaceae | Magnoliopsida |
| Rinorea breteleri Achound. | Violaceae | Magnoliopsida |
| Rinorea caudata (Oliv.) Kuntze | Violaceae | Magnoliopsida |
| Rinorea cerasifolia M.Brandt | Violaceae | Magnoliopsida |
| Rinorea dentata (P.Beauv.) Kuntze | Violaceae | Magnoliopsida |
| Rinorea exappendiculata Engl. | Violaceae | Magnoliopsida |
| Rinorea gabunensis Engl. | Violaceae | Magnoliopsida |
| Rinorea gilletii De Wild. | Violaceae | Magnoliopsida |
| Rinorea johnstonii (Stapf) M.Brandt | Violaceae | Magnoliopsida |
| Rinorea kamerunensis Engl. | Violaceae | Magnoliopsida |
| Rinorea ledermannii Engl. | Violaceae | Magnoliopsida |
| Rinorea leiophylla M.Brandt | Violaceae | Magnoliopsida |
| Rinorea longisepala Engl. | Violaceae | Magnoliopsida |
| Rinorea oblongifolia (C.H.Wright) C.Marquand ex Chipp | Violaceae | Magnoliopsida |
| Rinorea preussii Engl. | Violaceae | Magnoliopsida |
| Rinorea spec.1 Chaillu Aubl. | Violaceae | Magnoliopsida |
| Rinorea subintegrifolia (P.Beauv.) Kuntze | Violaceae | Magnoliopsida |
| Rinorea subsessilis M.Brandt | Violaceae | Magnoliopsida |
| Rinorea umbricola Engl. | Violaceae | Magnoliopsida |
| Rinorea verrucosa Chipp | Violaceae | Magnoliopsida |
| Rinorea welwitschii (Oliv.) Kuntze | Violaceae | Magnoliopsida |
| Rinorea woermanniana (Büttner) Engl. | Violaceae | Magnoliopsida |
| Rinorea zenkeri Engl. | Violaceae | Magnoliopsida |
| Cissus barbeyana De Wild. & T.Durand | Vitaceae | Magnoliopsida |
| Cissus leonardii Dewit | Vitaceae | Magnoliopsida |
| Cissus producta Afzel. | Vitaceae | Magnoliopsida |
| Cissus prunifera Desc. | Vitaceae | Magnoliopsida |
| Leea guineensis G.Don | Vitaceae | Magnoliopsida |
| Korupodendron songweanum Litt & Cheek | Vochysiaceae | Magnoliopsida |
| Xyris capensis Thunb. | Xyridaceae | Magnoliopsida |
| Xyris filiformis Lam. | Xyridaceae | Magnoliopsida |
| Xyris imitatrix Malme | Xyridaceae | Magnoliopsida |
| Aframomum cereum (Hook.f.) K.Schum. | Zingiberaceae | Magnoliopsida |
| Aframomum pilosum (Oliv. & T.Hanb.) K.Schum. | Zingiberaceae | Magnoliopsida |
| Costus dinklagei K.Schum. | Zingiberaceae | Magnoliopsida |
| Costus fenestralis Maas & H.Maas | Zingiberaceae | Magnoliopsida |
| Costus gabonensis Koechlin | Zingiberaceae | Magnoliopsida |
| Costus lateriflorus Baker | Zingiberaceae | Magnoliopsida |
| Costus ligularis Baker | Zingiberaceae | Magnoliopsida |
| Costus lilaceus Maas & H.Maas | Zingiberaceae | Magnoliopsida |
| Costus maboumiensis Pellegr. | Zingiberaceae | Magnoliopsida |
| Costus phyllocephalus K.Schum. | Zingiberaceae | Magnoliopsida |
| Costus tappenbeckianus J.Braun & K.Schum. | Zingiberaceae | Magnoliopsida |
| Renealmia africana Benth. | Zingiberaceae | Magnoliopsida |
| Renealmia congoensis Gagnep. | Zingiberaceae | Magnoliopsida |
| Renealmia densispica Koechlin | Zingiberaceae | Magnoliopsida |
